# Designing three-level cluster randomized trials to assess treatment effect heterogeneity

**DOI:** 10.1093/biostatistics/kxac026

**Published:** 2022-07-21

**Authors:** Fan Li, Xinyuan Chen, Zizhong Tian, Denise Esserman, Patrick J Heagerty, Rui Wang

**Affiliations:** Department of Biostatistics, Yale University School of Public Health, New Haven, CT 06510, USA; Department of Mathematics and Statistics, Mississippi State University, MS 39762, USA; Division of Biostatistics and Bioinformatics, Department of Public Health Sciences, Pennsylvania State University, Hershey, PA 17033, USA; Department of Biostatistics, Yale University School of Public Health, New Haven, CT 06510, USA; Department of Biostatistics, University of Washington, Seattle, WA 98195, USA; Department of Biostatistics, Harvard T. H. Chan School of Public Health, Boston, MA 02115, USA and Department of Population Medicine, Harvard Pilgrim Health Care Institute and Harvard Medical School, Boston, MA 02215, USA

**Keywords:** Design effect, Effect modification, Heterogeneous treatment effect, Intraclass correlation coefficient, Nested exchangeable correlation structure, Power calculation

## Abstract

Cluster randomized trials often exhibit a three-level structure with participants nested in subclusters such as health care providers, and subclusters nested in clusters such as clinics. While the average treatment effect has been the primary focus in planning three-level randomized trials, interest is growing in understanding whether the treatment effect varies among prespecified patient subpopulations, such as those defined by demographics or baseline clinical characteristics. In this article, we derive novel analytical design formulas based on the asymptotic covariance matrix for powering confirmatory analyses of treatment effect heterogeneity in three-level trials, that are broadly applicable to the evaluation of cluster-level, subcluster-level, and participant-level effect modifiers and to designs where randomization can be carried out at any level. We characterize a nested exchangeable correlation structure for both the effect modifier and the outcome conditional on the effect modifier, and generate new insights from a study design perspective for conducting analyses of treatment effect heterogeneity based on a linear mixed analysis of covariance model. A simulation study is conducted to validate our new methods and two real-world trial examples are used for illustrations.

## 1. Introduction

Pragmatic cluster randomized trials (CRTs) are increasingly popular for comparative effectiveness research within health care delivery systems based on real-world interventions and broadly representative patient populations. While the average treatment effect (ATE) has typically been the primary focus in planning pragmatic trials, interest is growing in understanding whether the treatment effect varies among prespecified patient subpopulations, such as those defined by demographics or baseline clinical characteristics. By design, the flexible inclusion of a range of clusters and patients to mimic real-world practice in pragmatic trials naturally induces more heterogeneity, an aspect that should be reflected at the design stage and which invites the study of the associated factors that may lead to variation in treatment effects.

While data-driven *exploratory* heterogeneity of treatment effect (HTE) analysis can be *ad hoc* and used to generate hypotheses for future studies, *confirmatory* HTE analysis is often hypothesis driven, and carried out based on prespecified effect modifiers identified from either prior data or subject-matter knowledge (Wang and Ware, 2013). A recent systematic review reported that 16 out of 64 health-related CRTs published between 2010 and 2016 examined HTE among prespecified demographic patient subgroups but also noted a lack of methods for designing CRTs to enable the assessment of confirmatory HTE (Starks *and others*, 2019). To this end, Yang *and others* (2020) proposed an analytical power calculation procedure for HTE analysis in CRTs with two-level data where patients are nested in clusters. Although planning CRTs for assessing the ATE only requires accounting for the outcome intraclass correlation coefficient (ICC), planning CRTs for assessing the HTE additionally requires accounting for the covariate-ICC (or equivalently, ICC of the effect modifier). The covariate-ICC captures the fraction of between-cluster covariate variation relative to the total of between- and within-cluster covariate variation and plays an important role in sample size determination for HTE analysis.

Although these two distinct types of ICCs were identified as essential ingredients for estimating the power of the HTE test in two-level CRTs, analytical design formulas are currently unavailable for pragmatic trials exhibiting a three-level structure. As a first example, the Health and Literacy Intervention (HALI) trial in Kenya is a three-level CRT studying a literacy intervention to improve early literacy outcomes (Jukes *and others*, 2017). In the HALI trial, continuous literacy outcomes are collected for children (participants), who are nested within schools (subclusters), which are further nested in Teacher Advisory Center (TAC) tutor zone (cluster); randomization is carried out at the TAC tutor zone level. Beyond studying the ATE on the literacy outcomes, one may be interested in detecting effect modification by different levels of the baseline literacy measurements to see whether the intervention is more effective in improving spelling or reading test scores among those with lower baseline scores. Power analysis for such an interaction test requires accounting for the covariate-ICCs and outcome-ICCs within each school and across different schools. In a second example, the Strategies to Reduce Injuries and Develop Confidence in Elders (STRIDE) trial (Gill *and others*, 2021) aims to study the effect of a multifactorial fall prevention intervention program and recruited patients (participants) nested within clinics (subclusters), which were nested in health care systems (clusters); the randomization was at the clinic level and therefore the study can be considered as a subcluster randomized trial. The study included prespecified HTE analysis with patient-level effect modifiers such as age and gender, among others. Suppose the interest lies in detecting potential intervention effect modification by gender on the continuous outcome, concern score for falling, the power analysis for such an interaction test also required accounting for the covariate-ICCs and outcome-ICCs within each clinic and between different clinics. In both examples, as we discuss in Section 2.1, a direct application of the design formula developed in two-level CRTs can result in either conservative or anticonservative sample size estimates; therefore, the three-level hierarchical structure clearly requires new methods that enable a precise understanding of how both participant- and subcluster-level characteristics moderate the impact of new care innovations. Addressing this gap in three-level designs is further complicated by the fact that not only can the randomization be carried out at either the subcluster or cluster level but also the effect modifier can be measured at each level. To date, existing methods for power analysis in three-level trials were mainly developed for studying the ATE, with a closed-form *design effect* jointly determined by the between-subcluster outcome-ICC and the within-subcluster outcome-ICC (Heo and Leon, 2008; Teerenstra *and others*, 2010). Dong *and others* (2018) have developed sample size formulas for HTE analysis with a univariate effect modifier under cluster-level randomization. However, the essential role of covariate-ICCs was neglected in their design formulas, which lead to under-powered trials as we demonstrate in Section 3. Beyond Dong *and others* (2018), computationally efficient and yet accurate power analysis procedures are lacking to ensure sufficient power for HTE analyses in three-level trials.

In this article, we contribute novel design formulas to address multilevel effect modification in trials with a three-level structure, without restrictions on the level of randomization or the level at which the effect modifier is measured. We characterize a *nested exchangeable correlation structure* for both the effect modifier and the outcome conditional on the effect modifier and derive new sample size expressions for HTE analysis based on the asymptotic covariance matrix corresponding to a linear mixed analysis of covariance (LM-ANCOVA) model. With a small set of design assumptions, our closed-form expressions can adequately capture key aspects of the data generating processes that contribute to the variance of the interaction effect parameters are a good approximation to the true Monte Carlo variance for the sample size of interest (as we demonstrate in Section 3) and therefore obviate the need for exhaustive power calculations by simulations. Based on either a univariate effect modifier or multivariate effect modifiers, we prove in Section 2 that both the between-subcluster and within-subcluster covariate-ICCs are key elements in the asymptotic variance expression of the HTE estimator. Beyond power analysis for the HTE, in Section 2.3, we point out that the LM-ANCOVA framework can be used for more efficient analyses of the ATE, thus allowing the use of familiar sample size equations for testing the ATE. We report a simulation study to validate our new methods in Section 3 and provide two concrete examples in Section 4. Section 5 concludes.

## 2. Power analyses for confirmatory HTE analysis in three-level trials

We consider a parallel-arm design with a three-level data structure, including the common scenario where participants (level 1) are nested in subclusters (level 2), which are nested in clusters (level 3). Let $Y_{ijk}$ be the outcome from participant $k$ ($k=1,\ldots,m$) from subcluster $j$ ($j=1,\ldots,n_s$) in cluster $i$ ($i=1,\ldots,n_c$). For design purposes, we assume each cluster includes an equal number of subclusters ($n_s$), and each subcluster includes an equal number of participants ($m$). We denote $W_{ijk}=1$ if participant $k$ in subcluster $j$ of cluster $i$ receives treatment and $W_{ijk}=0$ if the participant receives usual care. While randomization is more frequently carried out at the cluster or subcluster level in three-level designs, our framework also allows participant-level randomization as we elaborate on below.

For prespecified HTE analysis with a set of $p$ effect modifiers, ${\boldsymbol X}_{ijk}=(X_{ijk1},\ldots, X_{ijkp})^T$, we consider the following LM-ANCOVA model to formulate the HTE test:


(2.1) \begin{equation*}\label{eq:lme}
Y_{ijk} = \beta_1+\beta_2 W_{ijk}+\boldsymbol{\beta}_3^T{\boldsymbol X}_{ijk}+\boldsymbol{\beta}_4^T W_{ijk}{\boldsymbol X}_{ijk}+\gamma_i+u_{ij}+\epsilon_{ijk},
\end{equation*}

where $\gamma_i\sim \mathcal{N}(0,\sigma_{\gamma}^2)$ is the random intercept at the cluster level, $u_{ij}\sim \mathcal{N}(0,\sigma_u^2)$ is the random intercept at the subcluster level, and $\epsilon_{ijk}\sim \mathcal{N}(0,\sigma_{\epsilon}^2)$ is the participant-level random error. We adopt the conventional assumption in CRTs that the random effects are mutually independent and assume the absence of additional random variations. In model (2.1), $\beta_1$ is the intercept parameter, $\beta_2$ is the main effect of the treatment, $\boldsymbol{\beta}_3$ and $\boldsymbol{\beta}_4$ are the main covariate effects and the treatment-by-covariate interactions. The null hypothesis of no systematic HTE across subpopulations defined by ${\boldsymbol X}_{ijk}$ can be formulated as $\mathcal{H}_0$: $\boldsymbol{\beta}_4=\boldsymbol{0}$. Putting $\mathcal{H}_0$ in the context of a single binary effect modifier where $X_{ijk}=1$ for female and $X_{ijk}=0$ for nonfemale, $\beta_1$ represents the mean outcome among the nonfemale subgroup without receiving treatment, $\beta_2$ represents the treatment effect for the nonfemale subgroup, $\beta_3$ represents the mean outcome difference between the gender subgroups, and the scalar parameter $\beta_4$ encodes the difference in treatment effect between the gender subgroups, and a two-sided HTE test can proceed with the Wald statistic based on a standard normal sampling distribution. Of note, when ${\boldsymbol X}_{ijk}$ is mean centered, the interpretations of $\beta_1$ and $\beta_2$ will change (we discuss the interpretation of $\beta_2$ after mean-centering covariates in Section 2.3), but the interpretations of $\boldsymbol{\beta}_3$ and $\boldsymbol{\beta}_4$ remain unchanged, and therefore mean-centering covariates does not affect the HTE test for $\mathcal{H}_0:\boldsymbol{\beta}_4=\boldsymbol{0}$.

We assume the allocation ratio to the treatment and control groups is $\overline{W}/(1-\overline{W})$, with $\overline{W}=\mathbb{E}(W_{ijk})\in(0,1)$. We consider three design configurations: (i) CRT with randomization at the cluster level such that $n_c\overline{W}$ clusters are randomized to treatment and $n_c(1-\overline{W})$ clusters are randomized to usual care; (ii) CRT with randomization at the subcluster level such that within each cluster, $n_s\overline{W}$ subclusters are randomized to treatment and $n_s(1-\overline{W})$ subclusters are randomized to usual care; (iii) individually randomized trial (IRT) with a hierarchical structure such that within each subcluster, $m\overline{W}$ participants are randomized to treatment and $m(1-\overline{W})$ participants are randomized to usual care. In configurations (i) and (ii), we can further write the treatment indicator $W_{ijk}=W_i$, $\forall~j,k$ and $W_{ijk}=W_{ij}$, $\forall~k$, respectively, but we maintain $W_{ijk}$ throughout for a more unified presentation. To facilitate the characterization of the covariance parameters, we reparameterize model (2.1) by centering the treatment indicator and obtain


(2.2) \begin{equation*}\label{eq:lme2}
Y_{ijk} = b_1+b_2 (W_{ijk}-\overline{W})+{\boldsymbol b}_3^T{\boldsymbol X}_{ijk}+{\boldsymbol b}_4^T (W_{ijk}-\overline{W}){\boldsymbol X}_{ijk}+\gamma_i+u_{ij}+\epsilon_{ijk},
\end{equation*}

where $b_1=\beta_1+\beta_2\overline{W}$, $b_2=\beta_2$, ${\boldsymbol b}_3=\boldsymbol{\beta}_3+\boldsymbol{\beta}_4\overline{W,}$ and ${\boldsymbol b}_4=\boldsymbol{\beta}_4$. Let $\sigma^2_{y|x}$ denote the total variance of the outcome conditional on ${\boldsymbol X}_{ijk}$. Under model (2.2), $\sigma^2_{y|x}=\sigma_{\gamma}^2+\sigma_u^2+\sigma_{\epsilon}^2$. The strength of dependency between two outcomes conditional on covariates can be characterized by two distinct outcome-ICCs. The within-subcluster outcome-ICC describes the strength of dependency between two outcomes within the same subcluster, or $\alpha_{0}=\text{corr}(Y_{ijk}, Y_{ijk'} | {\boldsymbol X}_{ijk}, {\boldsymbol X}_{ijk'}, W_{ijk},W_{ijk'})=(\sigma_{\gamma}^2+\sigma_u^2)/\sigma^2_{y|x},~~\forall~k\neq k'$. The between-subcluster outcome-ICC describes the degree of dependency between two outcomes from two different subclusters but within the same cluster, or $\alpha_{1}=\text{corr}(Y_{ijk}, Y_{ij'k'} | {\boldsymbol X}_{ijk}, {\boldsymbol X}_{ij'k'}, W_{ijk},W_{ij'k'})=\sigma_{\gamma}^2/\sigma^2_{y|x},~~\forall~j\neq j',~k\neq k'$. If we write ${\boldsymbol Y}_i=(Y_{i11},\ldots,Y_{i1m},\ldots,Y_{in_s,1},\ldots,Y_{1n_s,m})^T$, then the implied correlation structure for ${\boldsymbol Y}_i$ is nested exchangeable (Teerenstra *and others*, 2010) with the matrix expression given by


(2.3) \begin{equation*}\label{eq:nexY}
{\boldsymbol R}_i=(1-\alpha_{0}){\boldsymbol I}_{n_sm}+(\alpha_{0}-\alpha_{1}){\boldsymbol I}_{n_s} \otimes {\boldsymbol J}_{m}+\alpha_{1}{\boldsymbol J}_{n_sm},
\end{equation*}

where “$\otimes$” refers to the Kronecker operator, ${\boldsymbol I}_{d}$ and ${\boldsymbol J}_{d}$ are a $d\times d$ identity matrix and $d\times d$ matrix of ones, respectively. Define the collection of design points ${\boldsymbol Z}_{ijk}=(1, (W_{ijk}-\overline{W}), {\boldsymbol X}_{ijk}^T, (W_{ijk}-\overline{W}){\boldsymbol X}_{ijk}^T)^T$, and ${\boldsymbol Z}_i=({\boldsymbol Z}_{i11},\ldots, {\boldsymbol Z}_{in_s,m})^T$ as the design matrix for cluster $i$. Given values of the variance components, the best linear unbiased estimator of ${\boldsymbol b}=(b_1,b_2,{\boldsymbol b}_3^T,{\boldsymbol b}_4^T)^T$ is the generalized least squares (GLS) estimator, given by $\widehat{{\boldsymbol b}}=\left(\sum_{i=1}^{n_c} {\boldsymbol Z}_i^T{\boldsymbol R}_i^{-1}{\boldsymbol Z}_i\right)^{-1}\left(\sum_{i=1}^{n_c}{\boldsymbol Z}_i^T{\boldsymbol R}_i^{-1}{\boldsymbol Y}_i\right)$. Furthermore, $\sqrt{n_c}(\widehat{{\boldsymbol b}}-{\boldsymbol b})$ converges in distribution to a multivariate normal random variate with mean zero and covariance matrix $\boldsymbol{\Sigma}_{(\infty,n_s,m)}=\sigma^2_{y|x}\left(\lim_{n_c\rightarrow\infty}n_c^{-1}\sum_{i=1}^{n_c}{\boldsymbol Z}_i^T{\boldsymbol R}_i^{-1}{\boldsymbol Z}_i\right)^{-1}$. Then, the asymptotic variance of $\widehat{{\boldsymbol b}}_4$ (based on $\sqrt{n_c}$-regime) is the lower-right element of the square matrix $\boldsymbol{\Sigma}_{(\infty,n_s,m)}$. Design calculations for sample size and power then boil down to explicitly characterizing the key elements of $\boldsymbol{\Sigma}_{(\infty,n_s,m)}$, which we operationalize below.

### 2.1. Univariate effect modifier

**Fig. 1.**
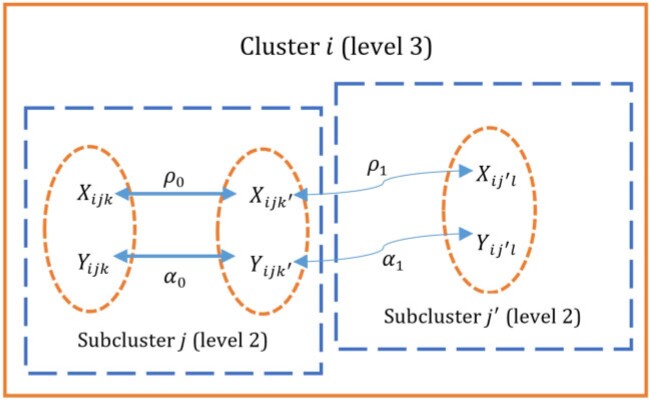
A graphical representation of the data structure in a cluster with two subclusters in a three-level trial. Each oval represents a participant (level 1) nested in a subcluster (level 2) nested in the cluster (level 3). The effect modifier $X$ and outcome $Y$ are measured for each participant with their respective covariate-IC7Cs and outcome-ICCs depicted. A thicker arrow indicates a stronger correlation between variables.

We first provide the analytical variance expressions when the treatment effect heterogeneity concerns a univariate effect modifier ($p=1$). For testing $\mathcal{H}_0$: $\beta_4=0$ with a two-sided Wald test with type I error rate $\alpha$ and target power $1-\lambda$, the required number of clusters, number of subclusters per cluster, and subcluster size, (or the total sample size), should satisfy $n_cn_sm\geq \sigma_4^2(z_{1-\alpha/2}-z_{1-\lambda})^2/\Delta_{\text{HTE}}^2$, where $z_{q}$ is the $q$th quantile of the standard normal distribution, $\Delta_{\text{HTE}}$ is the interaction effect size and $\sigma_4^2$ refers to the lower-right element of $n_sm\boldsymbol{\Sigma}_{(\infty,n_s,m)}$, or $\sigma_4^2=\lim_{n_c\rightarrow\infty}n_cn_sm\text{var}(\widehat{\beta}_4)$. Notice that $\boldsymbol{\Sigma}_{(\infty,n_s,m)}$ is a function of $n_s$, $m$ and decreases to zero as $n_s$ or $m$ increases. Therefore, we intentionally define $\sigma_4^2$ by multiplying $\boldsymbol{\Sigma}_{(\infty,n_s,m)}$ with $n_sm$ to facilitate efficiency comparison when randomization is conducted at different levels. This way, the limit of $\sigma_4^2$ will remain as a constant instead of zero if we also let $n_s$ or $m$ increase. To proceed, we write $\lambda_1=1-\alpha_0$, $\lambda_2=1+(m-1)\alpha_0-m\alpha_1$ and $\lambda_3=1+(m-1)\alpha_0+(n_s-1)m\alpha_1$ as three distinct eigenvalues of the nested exchangeable correlation structure (2.3) (Li *and others*, 2018), based on which an explicit inverse is given by ${\boldsymbol R}_i^{-1}=(1/\lambda_1){\boldsymbol I}_{n_sm}-(\lambda_2-\lambda_1)/(m\lambda_1\lambda_2){\boldsymbol I}_{n_s} \otimes {\boldsymbol J}_{m}-(\lambda_3-\lambda_2)/(n_sm\lambda_2\lambda_3){\boldsymbol J}_{n_sm}$; this analytical inverse plays a critical role in simplifying $\sigma_4^2$. For testing the HTE, we further assume a nested exchangeable correlation structure for the univariate effect modifier such that the marginal correlation matrix for ${\boldsymbol X}_i=(X_{i11},\ldots,X_{i1m},\ldots,X_{in_s,1},\ldots,X_{in_s,m})^T$ is


(2.4) \begin{equation*}\label{eq:nexX}
{\boldsymbol L}_i=(1-\rho_{0}){\boldsymbol I}_{n_s m}+(\rho_0-\rho_1){\boldsymbol I}_{n_s} \otimes {\boldsymbol J}_{m}+\rho_{1}{\boldsymbol J}_{n_s m},
\end{equation*}

where $\rho_0=\text{corr}(X_{ijk},X_{ijk'})$, $\forall~k\neq k'$, defines the within-subcluster covariate-ICC and $\rho_1=\text{corr}(X_{ijk},X_{ij'k'})$, $\forall~j\neq j'$ (without restrictions on $k$, $k'$), defines the between-subcluster covariate-ICC. Figure 1 provides a graphical representation of the data structure in a three-level trial. Defining $\zeta_1=1-\rho_0$, $\zeta_2=1+(m-1)\rho_0-m\rho_1$ and $\zeta_3=1+(m-1)\rho_0+(n_s-1)m\rho_1$ as three distinct eigenvalues of the nested exchangeable correlation structure (2.4), we first establish the following result.

Theorem 2.1 Defining $\sigma_x^2$ as the marginal variance of the univariate effect modifier, the limit variance $\sigma_4^2$ can be expressed as a function of the eigenvalues of the outcome-ICC and covariate-ICC matrices, and is dependent on the level of randomization. (i) When randomization is carried out at the cluster level, we have

\begin{equation*}
\sigma_{4,(3)}^2= \frac{\sigma_{y|x}^2}{\overline{W}(1-\overline{W})\sigma_x^2}\times\frac{n_s m}{\lambda_3^{-1}\zeta_3+(n_s-1)\lambda_2^{-1}\zeta_2+n_s(m-1)\lambda_1^{-1}\zeta_1}.
\end{equation*}

(ii) When randomization is carried out at the subcluster level, we have

\begin{equation*}
\sigma_{4,(2)}^2= \frac{\sigma_{y|x}^2}{\overline{W}(1-\overline{W})\sigma_x^2}\times\frac{m}{m\lambda_1^{-1} - \{1+(m-1)\rho_0\}\left(\lambda_1^{-1}-\lambda_2^{-1}\right)}.
\end{equation*}

(iii) When randomization is carried out at the participant level, we have $\sigma_{4,(1)}^2= \sigma_{y|x}^2/\left\{\overline{W}(1-\overline{W})\sigma_x^2\right\}\times \lambda_1$. (iv) The variances are linearly ordered such that $\sigma_{4,(3)}^2\geq \sigma_{4,(2)}^2\geq \sigma_{4,(1)}^2$, with equality obtained in the absence of residual clustering (e.g., $\lambda_1=\lambda_2=\lambda_3=1$ or $\sigma^2_\gamma=\sigma^2_u=0$).

The proof of Theorem 2.1 can be found in Appendix A of the Supplementary material available at *Biostatistics* online. Depending on the level of randomization, Theorem 2.1 provides a cascade of variance expressions to facilitate analytical power calculations. First, in the absence of any residual clustering in a three-level design such that $\alpha_0=\alpha_1=0$, we can write $\widetilde{\sigma}_{4,(3)}^2=\widetilde{\sigma}_{4,(2)}^2=\widetilde{\sigma}_{4,(1)}^2= {\sigma_{y|x}^2}/\{\overline{W}(1-\overline{W})\sigma_x^2\}$, which is essentially the limit variance of an interaction effect estimator in a simple ANCOVA model without clustering (Shieh, 2009). Therefore, we immediately notice that all three variances share the same form of the variance without clustering, ${\sigma_{y|x}^2}/\{\overline{W}(1-\overline{W})\sigma_x^2\}$, multiplied by a design effect expression characterizing the nontrivial residual clustering, which we denote by $\theta_{(3)}(n_s,m)=\sigma_{4,(3)}^2/\widetilde{\sigma}_{4,(3)}^2=n_sm/\{\lambda_3^{-1}\zeta_3+(n_s-1)\lambda_2^{-1}\zeta_2+n_s(m-1)\lambda_1^{-1}\zeta_1\}$, $\theta_{(2)}(m)=\sigma_{4,(2)}^2/\widetilde{\sigma}_{4,(2)}^2=m/\{m\lambda_1^{-1} - \{1+(m-1)\rho_0\}(\lambda_1^{-1}-\lambda_2^{-1})\}$, $\theta_{(1)}=\sigma_{4,(1)}^2/\widetilde{\sigma}_{4,(1)}^2=\lambda_1$, when the randomization unit is the cluster, subcluster, and participant, respectively. When the randomization is at the cluster level, Cunningham and Johnson (2016) showed that the design effect for testing the ATE in a three-level trial is unbounded when the subcluster size $m$ increases indefinitely. In sharp contrast, with a participant-level effect modifier, the design effect under a three-level CRT is bounded above even if the subcluster size goes to infinity, as $\theta_{(3)}(n_s,\infty)=(1-\alpha_0)/(1-\rho_0)$. Intuitively, the HTE parameter $\beta_4$ corresponds to a participant-level interaction covariate that varies within each subcluster whereas the ATE corresponds to a cluster-level treatment variable that only varies between clusters; this subtle difference underlies the difference in the two design effects. Interestingly, when $\rho_0\leq \alpha_0$, then $\theta_{(3)}(n_s,\infty)\leq 1$ and there can even be variance deflation in testing HTE under a three-level CRT relative to an individually randomized trials without clustering. For the subcluster randomized trial, $\theta_{(2)}(\infty)=(1-\alpha_0)/(1-\rho_0)=\theta_{(3)}(n_s,\infty)$, and the design effect is similarly bounded in the limit. The design effect $\theta_{(1)}$, however, does not depend on $n_s$ or $m$ and shares the same form with the one derived in Cunningham and Johnson (2016) for testing the ATE in a three-level IRT.

In addition, Theorem 2.1 demonstrates that randomization at a lower level leads to potential efficiency gain for estimating the interaction parameter $\beta_4$. This ordering result parallels that developed for testing the ATE in three-level designs (Cunningham and Johnson, 2016) and is intuitive because randomization at a lower level allows the within-cluster or within-subcluster comparisons to inform the estimation of the associated parameter. All three variances are increasing functions of the conditional outcome variance $\sigma_{y|x}^2$ and decreasing functions of the marginal covariate variance $\sigma_x^2$, matching the intuition that explained variation due to the effect modifier can lead to a HTE estimate with higher precision. Similar to the analysis of the ATE in IRTs or CRTs, a balanced design with equal randomization, $\overline{W}=1/2$, leads to the largest power for testing $\mathcal{H}_0$ under a fixed sample size. Interestingly, the covariate-ICCs enter the variance for estimating $\beta_4$ in an orderly fashion, that is, $\sigma_{4,(1)}^2$ is independent of $\rho_0$ and $\rho_1$, and $\sigma_{4,(2)}^2$ depends on $\rho_0$ but is free of $\rho_1$, whereas $\sigma_{4,(3)}^2$ depends on both $\rho_0$ and $\rho_1$. This observation suggests that the variance of $\widehat{\beta}_4$ only depends on the covariate-ICCs defined within each randomization unit but not between randomization units.

Theorem 2.1 helps demystify the relationships between the key ICC parameters and design efficiency for estimating the HTE. When randomization is carried out at the cluster level, larger values of covariate-ICCs, $\rho_0$, $\rho_1$, are always associated with a larger $\sigma_{4,(3)}^2$ (smaller power). This is expected because larger covariate-ICCs imply less per-participant information for estimating $\beta_4$ and therefore reduce statistical efficiency. The relationship between $\sigma_{4,(3)}^2$ and outcome-ICCs, $\alpha_0$, $\alpha_1$, can be nonmonotone and is graphically explored in Figure S1 of the Supplementary material available at *Biostatistics* online, where $\sigma_{4,(3)}^2$ exhibits a parabolic relationship with $\alpha_0$ and $\alpha_1$. This implies that a direct application of the existing design formula in Yang *and others* (2020) by equating $\alpha_0=\alpha_1$ and $\rho_0=\rho_1$ can lead to either conservative or anticonservative predictions for $\sigma_{4,(3)}^2$ and inaccurate sample size results in general. When randomization is carried out at the subcluster level, larger values of between-subcluster outcome-ICC, $\alpha_1$, is always associated with a smaller $\sigma_{4,(2)}^2$ (larger power), whereas a larger value of covariate-ICC, $\rho_0$, is always associated with a larger $\sigma_{4,(2)}^2$ (smaller power). However, $\sigma_{4,(2)}^2$ can be nonmonotone in the within-subcluster outcome-ICC, $\alpha_0$ (see Figure S2 of the Supplementary material available at *Biostatistics* online for a graphical exploration). Finally, when randomization is carried out at the participant level, a larger value of within-subcluster outcome-ICC, $\alpha_0$, is always associated with a smaller $\sigma_{4,(1)}^2$ (larger power), whereas $\sigma_{4,(1)}^2$ is independent of other ICC parameters. Table 1 provides a concise summary of these relationships and we further expand on these remarks in Appendix B of the Supplementary material available at *Biostatistics* online.

**Table 1.** Definition of ICC parameters, and a concise summary of the relationships between ICC parameters and the variance of the interaction effect parameter with a univariate participant-level effect modifier ($p=1$), under different levels of randomization

		Level of randomization		
ICC	Interpretation	Cluster	Subcluster	Participant
		$\sigma_{4,(3)}^2$	$\sigma_{4,(2)}^2$	$\sigma_{4,(1)}^2$
$\alpha_0$	The intraclass correlation parameter between outcomes from two participants within the same subcluster	$\curvearrowright$	$\curvearrowright$	$\Downarrow$
$\alpha_1$	The intraclass correlation parameter between outcomes from two participants in two different subclusters	$\curvearrowright$	$\Downarrow$	Indep
$\rho_0$	The intraclass correlation parameter between covariates from two participants within the same subcluster	$\Uparrow$	$\Uparrow$	Indep
$\rho_1$	The intraclass correlation parameter between covariates from two participants in two different subclusters	$\Uparrow$	Indep	Indep

“Indep” indicates that the variance is independent of and thus does not change with the specific ICC parameter; “$\Uparrow$” indicates a monotonically increasing relationship; “$\Downarrow$” indicates a monotonically decreasing relationship; and “$\curvearrowright$” indicates a nonmonotone and likely quadratic relationship.

Based on Theorem 2.1, further simplifications of the variance expressions are possible when the effect modifier is measured at the subcluster or cluster level. When the effect modifier is measured at the subcluster level (i.e., $\rho_0=1$) we obtain


(2.5) \begin{align*}
\sigma_{4,(3)}^2=& \frac{\sigma_{y|x}^2}{\overline{W}(1-\overline{W})\sigma_x^2}\times\frac{n_s}{\lambda_3^{-1}\{1+(n_s-1)\rho_1\}+\lambda_2^{-1}(n_s-1)(1-\rho_1)},\nonumber\\[-3pt]
\sigma_{4,(2)}^2=& \frac{\sigma_{y|x}^2}{\overline{W}(1-\overline{W})\sigma_x^2}\times\lambda_2.\label{eq:special}
\end{align*}

When the effect modifier is measured at the cluster level (i.e., $\rho_0=\rho_1=1$), $\sigma_{4,(3)}^2$ reduces to $\sigma_{4,(3)}^2={\sigma_{y|x}^2}/\{\overline{W}(1-\overline{W})\sigma_x^2\}\times\lambda_3$, but $\sigma_{4,(2)}^2$ remains identical to (2.5). In both cases, $\sigma_{4,(1)}^2$ is unaffected and remains the same as in Theorem 2.1.

### 2.2. Generalization to accommodate multivariate effect modifiers

Although a common practice for confirmatory tests of HTE is to consider a univariate effect modifier, the above analytical sample size procedure can be generalized to jointly test the interaction effects with multivariate effect modifiers. In this case, we write ${\boldsymbol X}_{ijk}=(X_{ijk1},\cdots,X_{ijkp})^T$ as the set of $p\geq2$ baseline covariates and $\boldsymbol{\beta}_4=(\beta_{41},\dots,\beta_{4p})^T$. We are interested in testing the global null hypothesis $\mathcal{H}_0$: $\boldsymbol{\beta}_4=0$ based on a Wald test. From the LM-ANCOVA model (2.2), the scaled GLS estimator $\sqrt{n_c}(\widehat{\boldsymbol{\beta}}_4-\boldsymbol{\beta}_4)$ is asymptotically normal with mean zero and variance equal to the lower-right $p\times p$ block of $\boldsymbol{\Sigma}_{(\infty,n_s,m)}$, which is denoted by $\boldsymbol{\Omega}_{4}$. This motivates a quadratic Wald test statistic $n_c\widehat{\boldsymbol{\beta}}_4^T\widehat{\boldsymbol{\Omega}}_{4}^{-1}\widehat{\boldsymbol{\beta}}_4$, which converges to a Chi-squared distribution $\chi^2(p,\vartheta)$ with $p$ degrees of freedom and noncentrality parameter $\vartheta=n_c\boldsymbol{\beta}_4^T\boldsymbol{\Omega}_4^{-1}\boldsymbol{\beta}_4$, where $\widehat{\boldsymbol{\Omega}}_4$ is the estimated covariance matrix of $\widehat{\boldsymbol{\beta}}_4$. For fixed effect size vector $\boldsymbol{\beta}_4=\boldsymbol{\Delta}_{\text{HTE}}$, the corresponding power equation of this Wald test is approximated by


(2.6) \begin{align*} \label{eq:mv-power}
1-\lambda = \int_{\chi^2_{1-\alpha}(p)}^\infty f\left(x;p,n_c\boldsymbol{\Delta}_{\text{HTE}}^T\boldsymbol{\Omega}_4^{-1}\boldsymbol{\Delta}_{\text{HTE}}\right)\mathrm{d} x,
\end{align*}

where $\chi^2_{1-\alpha}(p)$ is the $(1-\alpha)$ quantile of the central Chi-squared distribution with $p$ degrees of freedom and $f(x;p,\vartheta)$ is the probability density function of the $\chi^2(p,\vartheta)$ distribution. Fixing any two of $n_c$, $n_s$, or $m$, solving (2.6) for the other argument (for example, using the $\texttt{uniroot}$ function in $\texttt{R}$ and rounding to the next integer above) readily gives the required sample size to achieve a desired level of power; therefore, sample size determination for HTE analysis with multivariate effect modifiers boils down to the characterization of $\boldsymbol{\Omega}_4$ in explicit forms.

In Appendix C of the Supplementary material available at *Biostatistics* online, assuming a nested block exchangeable correlation structure for the multivariate effect modifiers, we prove a general version of Theorem 2.1. The general results establish the analytical forms of three covariance matrices of the $p$-vector of HTE estimators, $\boldsymbol{\Omega}_{4,(3)}$, $\boldsymbol{\Omega}_{4,(2)}$, and $\boldsymbol{\Omega}_{4,(1)}$, when randomization is carried out at the cluster level, subcluster level, and participant level, respectively. Furthermore, these covariance matrices have Löwner ordering such that $\boldsymbol{\Omega}_{4,(3)}\succeq\boldsymbol{\Omega}_{4,(2)}\succeq\boldsymbol{\Omega}_{4,(1)}$, with equality obtained in the absence of residual clustering (e.g., $\lambda_1=\lambda_2=\lambda_3=1$ or $\sigma^2_\gamma=\sigma^2_u=0$). Finally, with a univariate effect modifier measured at the participant level, the LM-ANCOVA model (2.1) can also be used to detect contextual effect modification by decomposing the covariate effect into a cluster-level, subcluster-level, and participant-level components to address different effects for the aggregated and lower-level variations (Raudenbush, 1997). Correspondingly, $\boldsymbol{X}_{ijk}=(X_{ijk}-\overline{X}_{ij},\overline{X}_{ij}-\overline{X}_i,\overline{X}_i)^T$ becomes the transformed vector of effect modifiers, and the more general Theorem derived in Appendix C of the Supplementary material available at *Biostatistics* online can be applied for analytical power analysis with contextual effect modification. We derive those explicit expressions in Appendix D of the Supplementary material available at *Biostatistics* online as an application of this more general result.

### 2.3. Connections to sample size requirements for studying the ATE

Even though the primary motivation for model (2.1) is to study HTE with prespecified effect modifiers, the LM-ANCOVA model also implies a covariate-adjusted estimator for the ATE. This is because the conditional ATE given by LM-ANCOVA is $\mathbb{E}(Y_{ijk}|{\boldsymbol X}_{ijk},W_{ijk}=1)-\mathbb{E}(Y_{ijk}|{\boldsymbol X}_{ijk},W_{ijk}=0)=\beta_2+\boldsymbol{\beta}_4^T{\boldsymbol X}_{ijk}$, and therefore the marginal ATE parameter can be given by integrating over the distribution of effect modifiers, $\Delta_{\text{ATE}}=\beta_2+\boldsymbol{\beta}_4^T\boldsymbol{\mu}_1$. Often times, a practical strategy is to globally mean center the collection of effect modifiers such that the sample mean is zero. Without loss of generality, we assume $\boldsymbol{\mu}_1=\boldsymbol{0}$ and therefore $\Delta_{\text{ATE}}=\beta_2$ can be interpreted as the covariate-adjusted ATE estimator. Such an observation is akin to the ANCOVA analysis of IRTs, for which model (2.1) is a direct generalization with multiple random effects. Different from prior work on ANCOVA analysis of IRTs without residual clustering (Yang and Tsiatis, 2001), we assume model (2.1) is correctly specified such that an explicit model-based variance expression of the ATE estimator can be used as a basis for study planning. Specifically, we denote the asymptotic variance expression of $\widehat{\beta}_2$ as $\sigma_2^2=\lim_{n_c\rightarrow\infty}n_c n_s m\text{var}(\widehat{\beta}_2)$, based on which the generic sample size requirement in a three-level design is $n_cn_sm\geq \sigma_2^2(z_{1-\alpha/2}-z_{1-\lambda})^2/\Delta_{\text{ATE}}^2$. Based on model (2.1), we provide a final result to facilitate power and sample size determination for testing the ATE in three-level designs (proof in Appendix E of the Supplementary material available at *Biostatistics* online).

Theorem 2.2 The limit variance expression of the covariate-adjusted ATE estimator depends on the unit of randomization and is at most a function of the outcome-ICCs. (a) When the randomization is carried out at the cluster level, we have $\sigma_{2,(3)}^2= {\sigma_{y|x}^2}/\{\overline{W}(1-\overline{W})\}\times\lambda_3$. (b) When randomization is carried out at the subcluster level, we have $\sigma_{2,(2)}^2= {\sigma_{y|x}^2}/\{\overline{W}(1-\overline{W})\}\times\lambda_2$. (c) When randomization is carried out at the participant level, we have $\sigma_{2,(1)}^2={\sigma_{y|x}^2}/\{\overline{W}(1-\overline{W})\}\times\lambda_1$. (d) The variances are linearly ordered such that $\sigma_{2,(3)}^2\geq \sigma_{2,(2)}^2\geq \sigma_{2,(1)}^2$, and equality is obtained when $\alpha_1=0$ ($\sigma_{2,(3)}^2=\sigma_{2,(2)}^2$) and $\alpha_0=\alpha_1=0$ ($\sigma_{2,(2)}^2=\sigma_{2,(1)}^2)$.

Theorem 2.2 implies that the design effect in a three-level design for estimating the ATE, as compared to an unclustered randomized design, is one eigenvalue of the outcome-ICC matrix ${\boldsymbol R}_i$, matching those derived by Cunningham and Johnson (2016) under three-level designs with one subtle difference. In Cunningham and Johnson (2016), the design effects were all derived assuming a linear mixed model without any effect modifiers and therefore the outcome variance and the outcome-ICCs in each eigenvalue are marginal with respect to effect modifiers. In contrast, the design effect expressions implied by Theorem 2.2 are parameterized by outcome variance and outcome-ICCs conditional on effect modifiers. As we demonstrate in Section 3, adjusting for effect modifiers can partially explain the residual variation of the outcomes at any level, and therefore may reduce either the outcome variance or the amount of residual correlation. In such a case, the conditional outcome-ICCs and conditional outcome variance are frequently no larger than their marginal counterparts, and therefore the ATE estimators based on LM-ANCOVA are likely to be more efficient than those under the unadjusted linear mixed model. This improvement in efficiency can directly translate into sample size savings. Finally, if the marginal variance of a univariate effect modifier is a unity such that $\sigma_x^2=1$, regardless of the level of randomization, the large-sample variance expression for the covariate-adjusted ATE estimator is identical to the large-sample variance expression for the HTE estimator, when the effect modifier is measured at or above the unit of randomization. Namely, $\sigma_{2,(1)}^2=\sigma_{4,(1)}^2$ regardless of the measurement level of the effect modifier; $\sigma_{2,(2)}^2=\sigma_{4,(2)}^2$ when the effect modifier is measured at the subcluster or cluster level; and $\sigma_{2,(3)}^2=\sigma_{4,(3)}^2$ when the effect modifier is measured at the cluster level.

## 3. Numerical studies

We carry out simulations to assess the finite-sample performance of the proposed sample size procedures for planning three-level trials. Our objectives are (i) assessing the accuracy of the proposed methods for powering three-level trials to detect HTE as well as the ATE and (ii) demonstrating, from a cost-effectiveness perspective, whether the sample size estimates based on Theorem 2.2 can be smaller than those estimated by the approach given in Cunningham and Johnson (2016), for the same level of power. Whenever applicable, we also compare our sample size results with those obtained from Dong *and others* (2018). To focus on the main idea, throughout we assume a univariate continuous effect modifier measured at the participant level. We consider designs with randomization at each of the three levels, leading to CRTs, subcluster randomized trials, and IRTs with a hierarchical structure. We assume equal randomization with $\overline{W}$=1/2, and a balanced design with equal numbers of subclusters $n_s$ in each cluster and equal subcluster sizes $m$.

For the first objective, we fix $\sigma_{y|x}^2$ and $\sigma_x^2$ at $1$, nominal type I error $\alpha=0.05$ and the desired power level $1-\lambda=0.8$; the remaining parameters are varied. Under each design, we consider two levels of subcluster sizes $m \in \{20, 50\}$ and two values for the number of subclusters per cluster $n_s \in \{4, 8\}$. Since typically $\alpha_0\ge \alpha_1$ and $\rho_0\ge \rho_1$, we chose $(\alpha_0, \alpha_1)\in\{(0.015, 0.01), (0.1, 0.05)\}$ to represent small and moderate conditional outcome-ICC, and $(\rho_0, \rho_1)\in\{(0.15, 0.1), (0.3, 0.15), (0.5, 0.3)\}$ to represent small, moderate, and large covariate-ICCs, based on ranges commonly reported in the CRT literature (Murray and Blitstein, 2003). To ensure that the predicted number of clusters is practical and mostly below $100$, we set $\Delta_{\text{HTE}}=0.1$ for all randomization scenarios, $\Delta_{\text{ATE}}=0.2$ for the CRTs, and $\Delta_{\text{ATE}}=0.1$ for the subcluster randomized trial or IRT. For each parameter combination, we estimate the number of clusters $\widehat{n}_c$ that ensures at least $80\%$ power and round to the nearest even integer above to ensure an exactly equal randomization. We then use the predicted cluster number $\widehat{n}_c$ to simulate correlated outcome data based on the LM-ANCOVA model and compute the empirical power of the Wald test for HTE or the ATE. When the randomization is carried out at the cluster level, we quantify the proportion of explained variation due to the effect modifier (details in Appendix F of the Supplementary material available at *Biostatistics* online) and use the method of Dong *and others* (2018) to obtain the required number of cluster $\widehat{n}^*_c$ to ensure at least 80$\%$ power, as a comparator to our new method.

We generate the individual-level outcomes using the LM-ANCOVA model (2.1) by fixing $\beta_1=1$ and $\beta_3=0.3$. For the HTE tests under each randomized design, we fix $\beta_2=0.2$, and $\beta_4=\Delta_{\text{HTE}}$. For studying the empirical power of the Wald test for the covariate-adjusted ATE, we fix $\beta_4=0.05$ such that $\beta_2=\Delta_{\text{ATE}}-\beta_4\mu_x$. We generate the correlated effect modifiers based on the linear mixed model, $X_{ijk}=\mu_x+a_i+b_{ij}+c_{ijk}$, where the global mean $\mu_x=1$, $a_i\sim\mathcal{N}(0,\sigma_x^2\rho_1)$, $b_{ij}\sim\mathcal{N}(0,\sigma_x^2(\rho_0-\rho_1))$, and $c_{ij}\sim\mathcal{N}(0,\sigma_x^2(1-\rho_0))$. The cluster-specific random intercept in LM-ANCOVA $\gamma_i\sim\mathcal{N}(0,\sigma_{y|x}^2\alpha_1)$, the subcluster-level random effect $u_{ij}\sim\mathcal{N}(0,\sigma_{y|x}^2(\alpha_0-\alpha_1))$, and the random error $\epsilon_{ijk}\sim\mathcal{N}(0,\sigma_{y|x}^2(1-\alpha_0))$. For each parameter configuration, we generate $5000$ hypothetical trials to evaluate the empirical type I error under the null and empirical power under the alternative. For each hypothetical trial, we fit the LM-ANCOVA model using restricted maximum likelihood estimation and carry out the corresponding Wald test for inference. To evaluate the covariate-adjusted Wald test for the ATE, we first globally mean-center the effect modifier before fitting the LM-ANCOVA. Finally, while the sample size estimation for HTE test and ATE test under a three-level design can generally be based on the standard normal distribution, we consider the $t$-distribution with the between-within degrees of freedom ($n_c-2$) only when studying the ATE under a CRT (standard normal distribution will still be used for testing the ATE in both subcluster randomized trials and IRTs). This choice represents an effective small-sample degrees of freedom correction specifically for CRTs with a limited number of clusters (and will have negligible difference from the standard normal when $n_c$ is sufficiently large, also see Chapter 2.4.2 in Pinheiro and Bates (2006)) and has been previously shown to maintain valid type I error rate (Li *and others*, 2016) in small CRTs for testing the ATE and therefore is adopted to more objectively assess the agreement between empirical and predicted power under that specific scenario. For this scenario, we also confirm in Table S1 of the Supplementary material available at *Biostatistics* online that the predicted variance of the ATE estimator based on Theorem 2.2 is close to the Monte Carlo variance.

For the second objective, given each effect size $\Delta_{\text{ATE}}$, we still generate the correlated outcome data using the LM-ANCOVA model as the above, but assume that the primary analysis of the ATE is based on a linear mixed model without the effect modifier. Correspondingly, we compute the required sample size using the formulas in the absence of any covariates. To operationalize those formulas, we obtain the total outcome variance marginalizing over the covariate distribution, $\sigma^2_y$. Furthermore, the unconditional outcome-ICCs can be approximated by $\widetilde{\alpha}_0\approx \omega\alpha_0+(1-\omega)\rho_0$ and $\widetilde{\alpha}_1\approx \omega\alpha_1+(1-\omega)\rho_1$, where the explicit form of $\omega$ is derived in Appendix F of the Supplementary material available at *Biostatistics* online. The required sample size for the unadjusted mixed model analysis is then estimated from the Theorem 2.2 but by replacing $\alpha_0$, $\alpha_1$ with $\widetilde{\alpha}_0$, $\widetilde{\alpha}_1$, and $\sigma^2_{y|x}$ with $\sigma^2_y$, namely, using the formulas in Cunningham and Johnson (2016). We compared the results with those estimated based on Theorem 2.2 to investigate saving in sample size due to adjustment for the univariate effect modifier. Finally, we also obtain the empirical type I error rate and empirical power by fitting the linear mixed model omitting $X_{ijk}$ to verify the accuracy of the sample size procedure without the effect modifier.

### 3.1. Results

Table 2 provides a summary of the estimated number of clusters using the proposed formula in Theorem 2.1, the empirical size, empirical power as well as predicted power by formula of the Wald test for HTE when $\Delta_{\text{HTE}}=0.1$, and when the randomization is carried out at the cluster level (differences from 80$\%$ power are due to rounding). The Wald test maintains the nominal type I error rate, which ensures the validity of the subsequent comparisons between the empirical and predicted power. Across all scenarios, the predicted power is in good agreement with the empirical power, even when there are as few as $10$ clusters. In Table 2, we also observe that the method of Dong *and others* (2018) often leads to much smaller sample size estimates ($\widehat{n}_c^*$) than the proposed method and therefore their method is consistently anticonservative. In Appendix F of the Supplementary material available at *Biostatistics* online, we have re-expressed their design formula using our notation and found that it does not depend on any covariate-ICC parameters. It is apparent from Table 2 that ignoring the covariate-ICC at each level during study planning can result in under-powered CRTs, especially in the presence of nontrivial covariate-ICCs.

**Table 2.** Estimated required number of clusters $\widehat{n}_c$ for the HTE test based on the proposed formula, empirical type I error (Emp. size), empirical power (Emp. power), as well as predicted power (Pred. power) for the HTE, test when randomization is at the cluster level. For studying power, the HTE effect size is fixed at $\Delta_{\text{HTE}}=0.1$. In the last two columns, we estimate the required sample size $\widehat{n}_c^*$ using the method of Dong *and others* (2018) and obtain the actual predicted power (Actual power) using our formula based on the estimated sample size $\widehat{n}_c^*$ to assess the degree to which the three-level CRT may be under-powered

Design parameters							Performance characteristics			Comparator	
$m$	$n_s$	$\alpha_0$	$\alpha_1$	$\rho_0$	$\rho_1$	$\widehat{n}_c$	Emp. size	Emp. power	Pred. power	$\widehat{n}_c^*$	Actual power
20	4	0.015	0.010	0.150	0.100	42	0.051	0.812	0.806	40	0.787
20	4	0.015	0.010	0.300	0.150	44	0.054	0.806	0.807	40	0.768
20	4	0.015	0.010	0.500	0.300	48	0.052	0.801	0.804	40	0.730
20	4	0.100	0.050	0.150	0.100	42	0.051	0.813	0.808	36	0.746
20	4	0.100	0.050	0.300	0.150	48	0.050	0.805	0.813	36	0.694
20	4	0.100	0.050	0.500	0.300	58	0.053	0.798	0.800	36	0.598
20	8	0.015	0.010	0.150	0.100	22	0.048	0.811	0.819	20	0.781
20	8	0.015	0.010	0.300	0.150	24	0.054	0.837	0.832	20	0.760
20	8	0.015	0.010	0.500	0.300	26	0.055	0.816	0.816	20	0.708
20	8	0.100	0.050	0.150	0.100	22	0.045	0.817	0.825	18	0.744
20	8	0.100	0.050	0.300	0.150	24	0.048	0.813	0.811	18	0.692
20	8	0.100	0.050	0.500	0.300	30	0.046	0.796	0.806	18	0.590
50	4	0.015	0.010	0.150	0.100	18	0.054	0.818	0.822	16	0.775
50	4	0.015	0.010	0.300	0.150	20	0.055	0.829	0.833	16	0.744
50	4	0.015	0.010	0.500	0.300	22	0.055	0.811	0.811	16	0.678
50	4	0.100	0.050	0.150	0.100	18	0.052	0.828	0.832	16	0.787
50	4	0.100	0.050	0.300	0.150	20	0.054	0.812	0.813	16	0.722
50	4	0.100	0.050	0.500	0.300	26	0.056	0.816	0.808	16	0.603
50	8	0.015	0.010	0.150	0.100	10	0.053	0.857	0.856	8	0.772
50	8	0.015	0.010	0.300	0.150	10	0.052	0.827	0.828	8	0.739
50	8	0.015	0.010	0.500	0.300	12	0.058	0.825	0.830	8	0.663
50	8	0.100	0.050	0.150	0.100	10	0.052	0.874	0.868	8	0.786
50	8	0.100	0.050	0.300	0.150	10	0.053	0.818	0.813	8	0.722
50	8	0.100	0.050	0.500	0.300	14	0.054	0.831	0.834	8	0.600

Under cluster randomization, Table 3 provides a summary of the estimated number of clusters using the proposed formula in Theorem 2.2, the empirical size, empirical power as well predicted power by formula of the Wald test for the covariate-adjusted ATE when $\Delta_{\text{ATE}}=0.2$. The Wald test still maintains valid type I error rate, with empirical power close to analytical prediction by our formula. This suggests that our variance expressions are accurate for study design purposes. Interestingly, even though the ATE size is twice the HTE effect size, the required sample size to achieve 80$\%$ power is not always larger for the HTE test compared to the ATE and can depend on the remaining design parameters. Finally, we demonstrate that ignoring the univariate effect modifier in the study design stage can lead to larger than necessary sample size estimates under a wide range of design configurations (Table S2 of the Supplementary material available at *Biostatistics* online). In fact, for the same ATE size, we find that the required sample size based on the Cunningham and Johnson (2016) formula may even be $50\%$ larger than that based on Theorem 2.2, as a result of explained variation. For example, as seen in Table S2 of the Supplementary material available at *Biostatistics* online, the adjusted outcome-ICCs $\alpha_0$ and $\alpha_1$ can be substantially smaller than their marginal counterparts, $\widehat{\alpha}_0$, $\widehat{\alpha}_1$, especially when the covariate-ICCs are farther away from zero.

**Table 3.** Estimated required number of clusters $\widehat{n}_c$ for the covariate-adjusted ATE test based on the proposed formula, empirical type I error (Emp. size), empirical power (Emp. power), as well as predicted power (Pred. power) for the covariate-adjusted ATE test when randomization is at the cluster level. For studying power, the ATE size is fixed at $\Delta_{\text{ATE}}=0.2$

Design parameters							Performance characteristics		
$m$	$n_s$	$\alpha_0$	$\alpha_1$	$\rho_0$	$\rho_1$	$\widehat{n}_c$	Emp. size	Emp. power	Pred. power
20	4	0.015	0.010	0.150	0.100	22	0.051	0.824	0.828
20	4	0.015	0.010	0.300	0.150	22	0.052	0.821	0.828
20	4	0.015	0.010	0.500	0.300	22	0.054	0.821	0.828
20	4	0.100	0.050	0.150	0.100	60	0.052	0.798	0.801
20	4	0.100	0.050	0.300	0.150	60	0.053	0.797	0.801
20	4	0.100	0.050	0.500	0.300	60	0.053	0.796	0.801
20	8	0.015	0.010	0.150	0.100	16	0.048	0.820	0.819
20	8	0.015	0.010	0.300	0.150	16	0.048	0.818	0.819
20	8	0.015	0.010	0.500	0.300	16	0.051	0.814	0.819
20	8	0.100	0.050	0.150	0.100	52	0.048	0.815	0.811
20	8	0.100	0.050	0.300	0.150	52	0.048	0.815	0.811
20	8	0.100	0.050	0.500	0.300	52	0.049	0.813	0.811
50	4	0.015	0.010	0.150	0.100	16	0.051	0.839	0.832
50	4	0.015	0.010	0.300	0.150	16	0.053	0.840	0.832
50	4	0.015	0.010	0.500	0.300	16	0.055	0.837	0.832
50	4	0.100	0.050	0.150	0.100	56	0.050	0.812	0.810
50	4	0.100	0.050	0.300	0.150	56	0.050	0.814	0.810
50	4	0.100	0.050	0.500	0.300	56	0.050	0.813	0.810
50	8	0.015	0.010	0.150	0.100	14	0.054	0.851	0.851
50	8	0.015	0.010	0.300	0.150	14	0.056	0.850	0.851
50	8	0.015	0.010	0.500	0.300	14	0.059	0.842	0.851
50	8	0.100	0.050	0.150	0.100	48	0.051	0.805	0.801
50	8	0.100	0.050	0.300	0.150	48	0.052	0.802	0.801
50	8	0.100	0.050	0.500	0.300	48	0.054	0.802	0.801

Simulation results for when the randomization is carried at the subcluster level and at the participant level are presented in Tables S3–S8 of the Supplementary material available at *Biostatistics* online. The patterns are qualitatively similar to the results in Tables 2 and 3 as well as Table S2 of the Supplementary material available at *Biostatistics* online and confirm that our analytical power procedure can accurately track the empirical power for the Wald test for HTE and ATE. In addition, these results confirm the ordering properties of the variances under different randomized designs, with the cluster-level randomization requiring the largest sample size and the participant-level randomization requiring the smallest sample size, for studying both HTE and the ATE. Finally, we observe that, for studying the ATE, the sample size saving by adjusting for the participant-level effect modifier is often the largest under a CRT and the smallest under an IRT with a hierarchical structure.

## 4. Two real CRT examples

We illustrate the proposed methods using two real trial examples, with a primary focus on the detection of confirmatory HTE. In both examples, we consider two-sided tests with a nominal $5\%$ type I error and $80\%$ power.

**Fig. 2.**
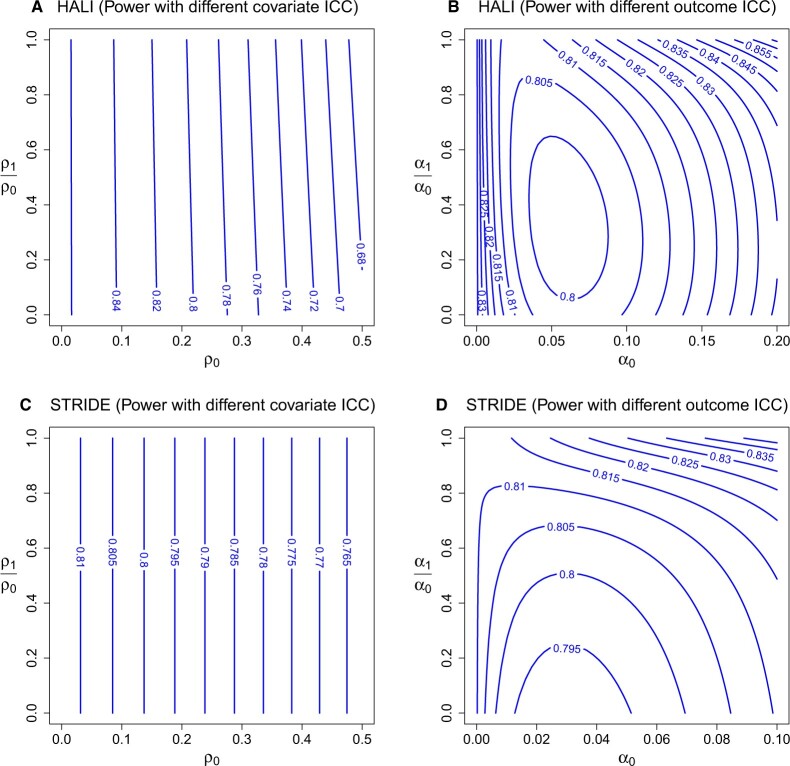
Estimated power contours for studying the heterogeneous treatment effect in the HALI and STRIDE trials as a function of the covariate and outcome-ICCs. (a) and (b) are based on the participant-level continuous effect modifier, baseline spelling score, in the HALI CRT ($n_c=24$, $n_s=4,$ and $m=25$), while (c) and (d) are based on the participant-level binary effect modifier, SRH, in the STRIDE subcluster randomized trial ($n_c=10$, $n_s=8,$ and $m=63$).

Example 4.1 (The HALI trial). The HALI trial compared the effect of a literacy intervention with usual instruction in terms of students’ literacy outcomes measured by performance on spelling and reading tests (Jukes *and others*, 2017). The study exhibits a three-level structure since the children participants are nested in schools (subclusters) which are further nested in TAC tutor zones (clusters); and the randomization of literacy intervention is carried out at the TAC level with $\overline{W}=1/2$. There are approximately $m=25$ children in each school, and on average $n_s=4$ schools per TAC tutor zone. We focus on the $9$-month spelling score as a continuous outcome and the baseline spelling score as a continuous potential effect modifier. Based on the estimates in Jukes *and others* (2017), we assume the conditional within-subcluster and between-subcluster outcome-ICCs as $\alpha_0=0.104$ and $\alpha_1=0.008$. We further assume the within-subcluster and between-subcluster covariate-ICCs $\rho_0=0.2$ and $\rho_1=\rho_0/2=0.1$. Using Theorem 2.1(a), the required number of TAC tutor zones to detect HTE by the baseline spelling score for a standardized effect size, $\Delta_{\text{HTE}}\sigma_x/\sigma_{y|x}=0.12$ (interpreted as the impact due to one standard deviation unit change in baseline spelling score on standard deviation unit change in the 9-month spelling score) is $n_c=24$. To explore the sensitivity of power to varying covariate- and outcome-ICC values, we estimate the power of the HTE test by varying $\rho_0\in[0,0.5]$, $\rho_1/\rho_0\in[0,1]$ with fixed outcome-ICC values, and by varying $\alpha_0\in[0,0.2]$, $\alpha_1/\alpha_0\in[0,1]$ with fixed covariate-ICCs. The power contours in Figures 2(a) and (b) indicate that the test power is sensitive to the magnitude of within-cluster covariate-ICC, but relatively insensitive to the magnitude of between-cluster covariate-ICC. While larger values of the within-cluster covariate-ICC can reduce the power of the HTE test to below $70\%$, variations of the outcome-ICCs within the range we considered maintain the power of the HTE test above $80\%$.

Example 4.2 (The STRIDE Trial). The STRIDE trial was a subcluster randomized trial comparing the effectiveness of a multifactorial fall prevention intervention program versus enhanced usual care on patient’s health outcomes (Gill *and others*, 2021). The study randomized primary care clinics to intervention conditions with allocation probability $\overline{W}=0.5$ and measured outcomes at the participant level; the clinics were nested within health centers, thus exhibiting a three-level structure. Each health center included about $n_s=8$ clinics, and the average clinic size was $m=63$. In this illustrative example, we considered the concern score for falling as a continuous outcome and self-rated health (SRH; measures whether one has good/excellent SRH) as a binary effect modifier measured at the participant level (Gill *and others*, 2021). We assumed the conditional within-subcluster and between-subcluster outcome-ICCs as $\alpha_0=0.01$ and $\alpha_1=0.005$, and further assumed the within-subcluster covariate-ICC $\rho_0=0.1$. Using Theorem 2.1(b), $n_c=10$ health centers would be required to detect HTE moderated with the SRH for a standardized effect size $\delta/\sigma_{y|x}=0.2$ (interpreted as the impact due to change in SRH on the standard deviation unit change in the concern score). To additionally explore the sensitivity of power to varying covariate- and outcome-ICC values, we present the power contours in Figures 2(c) and (d) as in Example 4.1. In particular, Figure 2(c) confirms that larger values of $\rho_0$ can decrease the power of the HTE test, but varying $\rho_1$ has no effect on the test power in a subcluster randomized trial. Varying values of the outcome-ICCs generally do not result in an under-powered HTE test, except when $\alpha_0\in[0.01,0.05]$ and $\alpha_1/\alpha_0\leq 0.4$ (a scenario with a moderate within-cluster outcome-ICC but a small between-subcluster outcome-ICC), in which case the power appears just below $80\%$. Overall, these two examples illustrate how our design expressions can be effectively operationalized in practical design situations and emphasize the critical role of the within-subcluster covariate-ICC in determining the power of the HTE test.

## 5. Concluding remarks

While prespecified HTE analysis has been a recognized goal in randomized trials, guidance to date on planning pragmatic trials involving clusters with HTE analysis is scarce. Through analytical derivations, we contribute a cascade of new variance expressions to allow for rigorous and yet computationally efficient design of pragmatic CRTs to power confirmatory HTE analysis with effect modifiers measured at each level, addressing a critical methodological gap in designing definitive pragmatic CRTs. Compared to the design of IRTs with HTE analysis (Brookes *and others*, 2004), the design of CRTs with HTE analysis requires more complex considerations due to the multilevel data structure and additional design parameters, especially the ICCs of the outcome as well as the effect modifier. While recent advancements in computational tools have made the use of a simulation-based power calculation an attractive alternative to analytical power predictions in clustered designs, such an approach is typically time-consuming and can quickly become impractical due to the need to search across multidimensional parameter spaces and repeatedly fitting multilevel models. Our proposed design formulas not only reduce the associated computational issues, but, more importantly, identify key aspects of the data generating processes that contribute to the study power. For example, the power of the HTE test is only affected by the HTE effect size but not the ATE size. Furthermore, the power of the HTE test only depends on the covariate-ICCs defined within each randomization unit but not between randomization units. Regardless of the level of randomization, the variance of the interaction effect estimator from the LM-ANCOVA model is proportional to the ratio of the conditional outcome variance and the variance of the effect modifier. In the context of a binary effect modifier, for example, the variance of the effect modifier is a function of the mean, and it follows that the variance of the interaction effect estimator reaches its minimum when the prevalence of the effect modifier is $0.5$ (holding all other factors constant). These insights greatly simplify power analysis by obviating the need to exhaust ancillary design parameters as would otherwise be required in a simulation-based power calculation procedure, and can possibly provide a basis for deriving optimal designs for testing effect modification.

There are additional aspects that we do not address in this article but remain important topics for future research. First, we have assumed equal cluster sizes to arrive at the main results. In a two-level CRT, Tong *and others* (2021) recently developed a correction factor for the variance formula of the HTE estimator under variable cluster sizes and found that the impact of cluster size variation is minimal with an participant-level effect modifier but can be more substantial with a cluster-level effect modifier. We anticipate these findings can be generalizable to three-level designs, although a careful derivation is needed to obtain the explicit correction factors when only the number of participants varies across subclusters, or only the number of subclusters varies across clusters, or both. Second, our design expressions developed under the LM-ANCOVA model are at best an approximation for three-level designs with a binary outcome. Analytical design formulas for powering the HTE test with a binary outcome that explicitly acknowledge the binomial variance structure warrant additional research. Finally, it would be worthwhile to generalize our results to further accommodate random covariate effects as an additional source of random variation. In general, the asymptotic variance expression tends to be analytically less tractable under random coefficients models and therefore requires a separate development.

## Supplementary Material

kxac026_Supplementary_Data

## Software

R code for implementing the proposed methods, simulations, and data examples are available at https://github.com/BillyTian/code_3levelHTE.
