# Supplementary material for: Designing three-level cluster randomized trials to assess treatment effect heterogeneity
Source: Biostatistics. 2022 Jul 21;24(4):833–49. doi: 10.1093/biostatistics/kxac026 (PMC10583727; doi:10.1093/biostatistics/kxac026)
Supplement: kxac026_Supplementary_Data [file kxac026_supplementary_data.zip › biosts-22066-File008.pdf]

Online Supplementary Material for "Designing three-level cluster randomized trials to assess treatment effect heterogeneity" by Li et al.

Web Appendix A Proof of Theorem 2.1

A.1 Part (a)

For the univariate case when the randomization is carried out at the cluster level. Recall the definitions in Section 2.1, the reparameterized linear mixed analysis of covariance (LM-ANCOVA) model is then given by

$$Y_{ijk} = b_1 + b_2(W_{ijk} - \overline{W}) + b_3 X_{ijk} + b_4(W_{ijk} - \overline{W})X_{ijk} + \gamma_i + u_{ij} + \epsilon_{ijk}, \tag{1}$$

where $W_{ijk} = W_i$ under the current randomization scenario, $b_1 = \beta_1 + \beta_2\overline{W}$, $b_2 = \beta_2$, $b_3 = \beta_3 + \beta_4\overline{W}$ and $b_4 = \beta_4$. Define the total variance of outcome conditional on X_{ijk} as $\sigma^2_{y|x} = \sigma^2_\gamma + \sigma^2_u + \sigma^2_\epsilon$, then (1) suggests the within-subcluster outcome-ICC, $\alpha_0 = (\sigma^2_\gamma + \sigma^2_u)/\sigma^2_{y|x}$, and the between-subcluster outcome-ICC, $\alpha_1 = \sigma^2_\gamma/\sigma^2_{y|x}$.

The implied correlation structure for $\boldsymbol{Y}_i = (Y_{i11}, \dots, Y_{i1m}, \dots, Y_{in_s,1}, \dots, Y_{1n_s,m})^T$ is nested exchangeable with matrix expression given as

$$\boldsymbol{R}_i = (1-\alpha_0)\boldsymbol{I}_{n_s m} + (\alpha_0 - \alpha_1)\boldsymbol{I}_{n_s} \otimes \boldsymbol{J}_m + \alpha_1 \boldsymbol{J}_{n_s m}, \tag{2}$$

where '$\otimes$' is the Kronecker product, $\boldsymbol{I}_d$ and $\boldsymbol{J}_d$ are $d \times d$ identity matrix and $d \times d$ matrix of ones, respectively. Define the collection of design points $\boldsymbol{Z}_{ijk} = (1, (W_i - \overline{W}), X_{ijk}, (W_i - \overline{W})X_{ijk})^T$, and $\boldsymbol{Z}_i = (\boldsymbol{Z}_{i11}, \dots, \boldsymbol{Z}_{in_s,m})^T$ as the design matrix for cluster i. Given values of the variance components, the best linear unbiased estimator of $\boldsymbol{b} = (b_1, b_2, b_3, b_4)^T$ is the Generalized Least Squares (GLS) estimator, given by $\widehat{\boldsymbol{b}} = \left(\sum_{i=1}^{n_c} \boldsymbol{Z}_i^T \boldsymbol{R}_i^{-1} \boldsymbol{Z}_i\right)^{-1} \left(\sum_{i=1}^{n_c} \boldsymbol{Z}_i^T \boldsymbol{R}_i^{-1} \boldsymbol{Y}_i\right)$.

As discussed in Section 2.1 in the main text, deriving the large-sample variance of $\sqrt{n_c}\widehat{\boldsymbol{b}}$ is equivalent to identifying the explicit expression of the limit variance matrix $\boldsymbol{\Sigma}_{(\infty,n_s,m)} = \sigma^2_{y|x}\boldsymbol{U}^{-1} = \sigma^2_{y|x}\left(\lim_{n_c\to\infty} n_c^{-1}\boldsymbol{U}_{(n_c,n_s,m)}\right)^{-1}$, where $\boldsymbol{U}_{(n_c,n_s,m)} = \sum_{i=1}^{n_c} \boldsymbol{Z}_i^T \boldsymbol{R}_i^{-1} \boldsymbol{Z}_i$ is a function of n_c, n_s and m. Specifically, $\sigma^2_{4,(3)} = \lim_{n_c\to\infty} n_c n_s m \text{var}(\widehat{b}_4)$ and $\lim_{n_c\to\infty} n_c \text{var}(\widehat{b}_4)$ is the lower-right element of $\boldsymbol{\Sigma}_\infty$.

Define $\lambda_1 = 1 - \alpha_0$, $\lambda_2 = 1 + (m-1)\alpha_0 - m\alpha_1$, and $\lambda_3 = 1 + (m-1)\alpha_0 + (n_s - 1)m\alpha_1$, then adopting the results in Li et al. (2018), we have

$$\boldsymbol{R}_i^{-1} = \frac{1}{\lambda_1}\boldsymbol{I}_{n_s m} - \frac{\lambda_2 - \lambda_1}{m\lambda_1\lambda_2}\boldsymbol{I}_{n_s} \otimes \boldsymbol{J}_m - \frac{\lambda_3 - \lambda_2}{n_s m \lambda_2 \lambda_3}\boldsymbol{J}_{n_s m} = c\boldsymbol{I}_{n_s m} + d\boldsymbol{I}_{n_s} \otimes \boldsymbol{J}_m + e\boldsymbol{J}_{n_s m},$$

where $c = 1/\lambda_1$, $d = -(\lambda_2 - \lambda_1)/(m\lambda_1\lambda_2)$, and $e = -(\lambda_3 - \lambda_2)/(n_s m \lambda_2 \lambda_3)$. We further define $\overline{X}_{ij} =$

$m^{-1}\sum_{k=1}^m X_{ijk}$ as the mean of covariate for each subcluster, and $\overline{X}_i = (n_s m)^{-1}\sum_{j=1}^{n_s}\sum_{k=1}^m X_{ijk}$ as the mean of covariate for each cluster, and based on the form of $\boldsymbol{R}_i^{-1}$, we can write $\boldsymbol{U}_{(n_c,n_s,m)} = c\boldsymbol{S}_{(n_c,n_s,m)} + d\boldsymbol{T}_{(n_c,n_s,m)} + e\boldsymbol{Q}_{(n_c,n_s,m)}$, where

$$\begin{aligned}
\boldsymbol{S}_{(n_c,n_s,m)} &= \sum_{i=1}^{n_c}\sum_{j=1}^{n_s}\sum_{k=1}^{m}\boldsymbol{Z}_{ijk}\boldsymbol{Z}_{ijk}^T \\
&= \sum_{i=1}^{n_c}\sum_{j=1}^{n_s}\sum_{k=1}^{m}\begin{bmatrix} 1 & W_i-\overline{W} & X_{ijk} & (W_i-\overline{W})X_{ijk} \\ W_i-\overline{W} & (W_i-\overline{W})^2 & (W_i-\overline{W})X_{ijk} & (W_i-\overline{W})^2X_{ijk} \\ X_{ijk} & (W_i-\overline{W})X_{ijk} & X_{ijk}^2 & (W_i-\overline{W})X_{ijk}^2 \\ (W_i-\overline{W})X_{ijk} & (W_i-\overline{W})^2X_{ijk} & (W_i-\overline{W})X_{ijk}^2 & (W_i-\overline{W})^2X_{ijk}^2 \end{bmatrix}, \\
\boldsymbol{T}_{(n_c,n_s,m)} &= \sum_{i=1}^{n_c}\sum_{j=1}^{n_s}\left(\sum_{k=1}^{m}\boldsymbol{Z}_{ijk}\right)\left(\sum_{k=1}^{m}\boldsymbol{Z}_{ijk}^T\right) \\
&= \sum_{i=1}^{n_c}\sum_{j=1}^{n_s}m^2\begin{bmatrix} 1 & W_i-\overline{W} & \overline{X}_{ij} & (W_i-\overline{W})\overline{X}_{ij} \\ W_i-\overline{W} & (W_i-\overline{W})^2 & (W_i-\overline{W})\overline{X}_{ij} & (W_i-\overline{W})^2\overline{X}_{ij} \\ \overline{X}_{ij} & (W_i-\overline{W})\overline{X}_{ij} & \overline{X}_{ij}^2 & (W_i-\overline{W})\overline{X}_{ij}^2 \\ (W_i-\overline{W})\overline{X}_{ij} & (W_i-\overline{W})^2\overline{X}_{ij} & (W_i-\overline{W})\overline{X}_{ij}^2 & (W_i-\overline{W})^2\overline{X}_{ij}^2 \end{bmatrix}, \\
\boldsymbol{Q}_{(n_c,n_s,m)} &= \sum_{i=1}^{n_c}\left(\sum_{j=1}^{n_s}\sum_{k=1}^{m}\boldsymbol{Z}_{ijk}\right)\left(\sum_{j=1}^{n_s}\sum_{k=1}^{m}\boldsymbol{Z}_{ijk}^T\right) \\
&= \sum_{i=1}^{n_c}(n_s m)^2\begin{bmatrix} 1 & W_i-\overline{W} & \overline{X}_{i} & (W_i-\overline{W})\overline{X}_{i} \\ W_i-\overline{W} & (W_i-\overline{W})^2 & (W_i-\overline{W})\overline{X}_{i} & (W_i-\overline{W})^2\overline{X}_{i} \\ \overline{X}_{i} & (W_i-\overline{W})\overline{X}_{i} & \overline{X}_{i}^2 & (W_i-\overline{W})\overline{X}_{i}^2 \\ (W_i-\overline{W})\overline{X}_{i} & (W_i-\overline{W})^2\overline{X}_{i} & (W_i-\overline{W})\overline{X}_{i}^2 & (W_i-\overline{W})^2\overline{X}_{i}^2 \end{bmatrix}.
\end{aligned}$$

To simplify the above matrices, we further define population-level parameters $\mu_r = \lim_{n_c\to\infty}(n_c n_s m)^{-1}\sum_{i=1}^{n_c}\sum_{j=1}^{n_s}\sum_{k=1}^m X_{ijk}^r$ as the moment of X_{ijk} across all clusters for $r=1,2$, $\tau_2 = \lim_{n_c\to\infty}(n_c n_s)^{-1}\sum_{i=1}^{n_c}\sum_{j=1}^{n_s}\overline{X}_{ij}^2$ as the second moment of subcluster-specific mean across all clusters, and $\eta_2 = \lim_{n_c\to\infty} n_c^{-1}\sum_{i=1}^{n_c}\overline{X}_i^2$ as the second moment of cluster-specific mean across all clusters. Denote $\sigma_w^2 = \text{var}(W_i)$, we can obtain the following limits

$$\boldsymbol{S} = \lim_{n_c\to\infty}\frac{1}{n_c}\boldsymbol{S}_{(n_c,n_s,m)} = n_s m\begin{bmatrix} 1 & 0 & \mu_1 & 0 \\ 0 & \sigma_w^2 & 0 & \mu_1\sigma_w^2 \\ \mu_1 & 0 & \mu_2 & 0 \\ 0 & \mu_1\sigma_w^2 & 0 & \mu_2\sigma_w^2 \end{bmatrix},$$

$$\boldsymbol{T} = \lim_{n_c\to\infty}\frac{1}{n_c}\boldsymbol{T}_{(n_c,n_s,m)} = n_s m^2\begin{bmatrix} 1 & 0 & \mu_1 & 0 \\ 0 & \sigma_w^2 & 0 & \mu_1\sigma_w^2 \\ \mu_1 & 0 & \tau_2 & 0 \\ 0 & \mu_1\sigma_w^2 & 0 & \tau_2\sigma_w^2 \end{bmatrix},$$

$$\boldsymbol{Q} = \lim_{n_c\to\infty}\frac{1}{n_c}\boldsymbol{Q}_{(n_c,n_s,m)} = n_s^2 m^2\begin{bmatrix} 1 & 0 & \mu_1 & 0 \\ 0 & \sigma_w^2 & 0 & \mu_1\sigma_w^2 \\ \mu_1 & 0 & \eta_2 & 0 \\ 0 & \mu_1\sigma_w^2 & 0 & \eta_2\sigma_w^2 \end{bmatrix}.$$

Then, we write $\boldsymbol{U}$ in four blocks such that $\boldsymbol{U} = c\boldsymbol{S} + d\boldsymbol{T} + e\boldsymbol{Q} = \begin{bmatrix} \boldsymbol{A} & \boldsymbol{B} \\ \boldsymbol{C} & \boldsymbol{D} \end{bmatrix}$, where $\boldsymbol{C} = \boldsymbol{B}$ and

$$\begin{aligned}
\boldsymbol{A} &= n_s m(c + dm + en_s m) \begin{bmatrix} 1 & 0 \\ 0 & \sigma_w^2 \end{bmatrix}, \\
\boldsymbol{B} &= n_s m(c + dm + en_s m)\mu_1 \begin{bmatrix} 1 & 0 \\ 0 & \sigma_w^2 \end{bmatrix} = \mu_1 \boldsymbol{A}, \\
\boldsymbol{D} &= n_s m(c\mu_2 + dm\tau_2 + en_s m\eta_2) \begin{bmatrix} 1 & 0 \\ 0 & \sigma_w^2 \end{bmatrix}.
\end{aligned}$$

Based on the block matrix inversion formula, we can derive the explicit form of the lower-right block in $\boldsymbol{U}^{-1}$, and therefore, the desired lower-right element of $\boldsymbol{\Sigma}_{(\infty,n_s,m)} = \sigma_{y|x}^2 \boldsymbol{U}^{-1}$. Notice that each block matrix in $\boldsymbol{U}$ is now a diagonal matrix, which makes the derivation simple. Specifically, the lower-right block of $\boldsymbol{\Sigma}_{(\infty,n_s,m)}$ is $\sigma_{y|x}^2(\boldsymbol{D} - \boldsymbol{C}\boldsymbol{A}^{-1}\boldsymbol{B})^{-1} = \sigma_{y|x}^2(\boldsymbol{D} - \mu_1\boldsymbol{B})^{-1}$, and its lower-right element is

$$\begin{aligned}
\lim_{n_c \to \infty} n_c \text{var}(\widehat{b}_4) &= \frac{\sigma_{y|x}^2}{n_s m \sigma_w^2 \{c(\mu_2 - \mu_1^2) + dm(\tau_2 - \mu_1^2) + en_s m(\eta_2 - \mu_1^2)\}} \qquad (3) \\
&= \frac{\sigma_{y|x}^2 \lambda_1 \lambda_2 \lambda_3}{n_s m \sigma_w^2 \{\lambda_2\lambda_3(\mu_2 - \mu_1^2) - \lambda_3(\lambda_2 - \lambda_1)(\tau_2 - \mu_1^2) - \lambda_1(\lambda_3 - \lambda_2)(\eta_2 - \mu_1^2)\}}.
\end{aligned}$$

We continue to simplify the formula using the covariate-ICC. Analogous to outcome-ICC, we introduce the nested exchangeable correlation structure for $\boldsymbol{X}_i = (X_{i11}, \ldots, X_{i1m}, \ldots, X_{in_s,1}, \ldots, X_{in_s,m})^T$ as

$$\boldsymbol{L}_i = (1 - \rho_0)\boldsymbol{I}_{n_s m} + (\rho_0 - \rho_1)\boldsymbol{I}_{n_s} \otimes \boldsymbol{J}_m + \rho_1 \boldsymbol{J}_{n_s m}, \qquad (4)$$

where the within-subcluster covariate-ICC and between-subcluster covariate-ICC are respectively defined as

$$\begin{aligned}
\rho_0 &= \frac{\mathbb{E}(X_{ijk}X_{ijk'}) - \mu_1^2}{\mu_2 - \mu_1^2}, \quad \forall\, k \neq k', \\
\rho_1 &= \frac{\mathbb{E}(X_{ijk}X_{ij'k'}) - \mu_1^2}{\mu_2 - \mu_1^2}, \quad \forall\, j \neq j', k \neq k'.
\end{aligned}$$

$\sigma_x^2 = \mu_2 - \mu_1^2$ is the marginal variance of the univariate effect modifier and $\zeta_1 = 1 - \rho_0$, $\zeta_2 = 1 + (m-1)\rho_0 - m\rho_1$, and $\zeta_3 = 1 + (m-1)\rho_0 + (n_s - 1)m\rho_1$ are three distinct eigenvalues of the nested exchangeable correlation structure (4).

Recall that

$$\begin{aligned}
\eta_2 &= \lim_{n_c \to \infty} \frac{1}{n_c(n_s m)^2} \sum_{i=1}^{n_c} \left(\sum_{j=1}^{n_s} \sum_{k=1}^{m} X_{ijk} \right)^2 \\
&= \lim_{n_c \to \infty} \frac{1}{n_c(n_s m)^2} \sum_{i=1}^{n_c} \left(\sum_{j=1}^{n_s} \sum_{k=1}^{m} X_{ijk}^2 + \sum_{j=1}^{n_s} \sum_{k \neq k'} X_{ijk}X_{ijk'} + \sum_{j \neq j'} \sum_{k \neq k'} X_{ijk}X_{ij'k'} \right)
\end{aligned}$$

$$= \frac{1}{n_s m}\mu_2 + \frac{n_c n_s m(m-1)\{\mu_1^2 + \rho_0(\mu_2 - \mu_1^2)\}}{n_c(n_s m)^2} + \frac{n_c n_s(n_s-1)m^2\{\mu_1^2 + \rho_1(\mu_2 - \mu_1^2)\}}{n_c(n_s m)^2}$$
$$= \frac{1}{n_s m}\{\mu_2 - \mu_1^2 + m n_s \mu_1^2 + (m-1)\rho_0(\mu_2 - \mu_1^2) + m(n_s-1)\rho_1(\mu_2 - \mu_1^2)\}.$$

This gives

$$\eta_2 - \mu_1^2 = \frac{1}{n_s m}\{\mu_2 - \mu_1^2 + (m-1)\rho_0(\mu_2 - \mu_1^2) + m(n_s-1)\rho_1(\mu_2 - \mu_1^2)\} = \frac{(\mu_2 - \mu_1^2)\zeta_3}{n_s m} = \frac{\sigma_x^2 \zeta_3}{n_s m}.$$

Similarly, we derive

$$\tau_2 = \lim_{n_c\to\infty} \frac{1}{n_c n_s m^2} \sum_{i=1}^{n_c}\sum_{j=1}^{n_s}\left(\sum_{k=1}^{m} X_{ijk}^2 + \sum_{k\neq k'} X_{ijk}X_{ijk'}\right) = \frac{\mu_2}{m} + \frac{m-1}{m}\{\mu_1^2 + \rho_0(\mu_2 - \mu_1^2)\},$$

which allows us to write

$$\tau_2 - \mu_1^2 = \frac{\sigma_x^2\{1 + (m-1)\rho_0\}}{m} = \frac{\sigma_x^2\{\zeta_3 + (n_s-1)\zeta_2\}}{n_s m}.$$

Plugging all these results into the large-sample variance expression (3), we can obtain

$$\lim_{n_c\to\infty} n_c \mathrm{var}(\widehat{\beta}_4) = \lim_{n_c\to\infty} n_c \mathrm{var}(\widehat{b}_4) = \frac{\sigma_{y|x}^2 \lambda_1\lambda_2\lambda_3}{\sigma_w^2\sigma_x^2\{n_s(m-1)\lambda_2\lambda_3\zeta_1 + (n_s-1)\lambda_1\lambda_3\zeta_2 + \lambda_1\lambda_2\zeta_3\}}.$$

Re-organizing the equation, we can conclude that

$$\sigma_{4,(3)}^2 = \lim_{n_c\to\infty} n_c n_s m \mathrm{var}(\widehat{\beta}_4) = \frac{\sigma_{y|x}^2}{\overline{W}(1-\overline{W})\sigma_x^2} \times \frac{n_s m}{n_s(m-1)\lambda_1^{-1}\zeta_1 + (n_s-1)\lambda_2^{-1}\zeta_2 + \lambda_3^{-1}\zeta_3},$$

which reaches the asymptotic variance when the randomization is carried out at the cluster level.

A.2 Part (b)

We then consider the univariate case when randomization is carried out at the subcluster level, where $W_{ijk} = W_{ij}$. Using the same reparametrization and procedures, we aim at the lower-right element of the limit matrix $\sigma_{y|x}^2 \boldsymbol{U}^{-1} = \sigma_{y|x}^2(\lim_{n_c\to\infty} n_c^{-1}\boldsymbol{U}_{(n_c,n_s,m)})^{-1}$, where $\boldsymbol{U}_{(n_c,n_s,m)} = \sum_{i=1}^{n_c} \boldsymbol{Z}_i^T \boldsymbol{R}_i^{-1}\boldsymbol{Z}_i$. Define the collection of design points as $\boldsymbol{Z}_{ijk} = (1, (W_{ij} - \overline{W}), X_{ijk}, (W_{ij} - \overline{W})X_{ijk})^T$, we pursue the component matrices referred in $\boldsymbol{U}_{(n_c,n_s,m)} = c\boldsymbol{S}_{(n_c,n_s,m)} + d\boldsymbol{T}_{(n_c,n_s,m)} + e\boldsymbol{Q}_{(n_c,n_s,m)}$ and $\boldsymbol{U} = c\boldsymbol{S} + d\boldsymbol{T} + e\boldsymbol{Q}$.

$$\begin{aligned}
\boldsymbol{S}_{(n_c,n_s,m)} &= \sum_{i=1}^{n_c}\sum_{j=1}^{n_s}\sum_{k=1}^{m} \boldsymbol{Z}_{ijk}\boldsymbol{Z}_{ijk}^T \\
&= \sum_{i=1}^{n_c}\sum_{j=1}^{n_s}\sum_{k=1}^{m} \begin{bmatrix} 1 & W_{ij} - \overline{W} & X_{ijk} & (W_{ij} - \overline{W})X_{ijk} \\ W_{ij} - \overline{W} & (W_{ij} - \overline{W})^2 & (W_{ij} - \overline{W})X_{ijk} & (W_{ij} - \overline{W})^2 X_{ijk} \\ X_{ijk} & (W_{ij} - \overline{W})X_{ijk} & X_{ijk}^2 & (W_{ij} - \overline{W})X_{ijk}^2 \\ (W_{ij} - \overline{W})X_{ijk} & (W_{ij} - \overline{W})^2 X_{ijk} & (W_{ij} - \overline{W})X_{ijk}^2 & (W_{ij} - \overline{W})^2 X_{ijk}^2 \end{bmatrix},
\end{aligned}$$

which implies an $\boldsymbol{S}$ matrix that is identical to what is presented in Part (a). Actually, we find that the limit, $\boldsymbol{S}$, will be the same no matter at which level the randomization is carried out. We further express

$$\begin{aligned}
\boldsymbol{T}_{(n_c,n_s,m)} &= \sum_{i=1}^{n_c}\sum_{j=1}^{n_s}\left(\sum_{k=1}^{m}\boldsymbol{Z}_{ijk}\right)\left(\sum_{k=1}^{m}\boldsymbol{Z}_{ijk}^T\right) \\
&= \sum_{i=1}^{n_c}\sum_{j=1}^{n_s} m^2 \begin{bmatrix} 1 & W_{ij}-\overline{W} & \overline{X}_{ij} & (W_{ij}-\overline{W})\overline{X}_{ij} \\ W_{ij}-\overline{W} & (W_{ij}-\overline{W})^2 & (W_{ij}-\overline{W})\overline{X}_{ij} & (W_{ij}-\overline{W})^2\overline{X}_{ij} \\ \overline{X}_{ij} & (W_{ij}-\overline{W})\overline{X}_{ij} & \overline{X}_{ij}^2 & (W_{ij}-\overline{W})\overline{X}_{ij}^2 \\ (W_{ij}-\overline{W})\overline{X}_{ij} & (W_{ij}-\overline{W})^2\overline{X}_{ij} & (W_{ij}-\overline{W})\overline{X}_{ij}^2 & (W_{ij}-\overline{W})^2\overline{X}_{ij}^2 \end{bmatrix}.
\end{aligned}$$

We find that its corresponding limit, $\boldsymbol{T}$, will also has the same form as in the cluster-level randomization case in Part (a). And, we finally express

$$\boldsymbol{Q}_{(n_c,n_s,m)} = \sum_{i=1}^{n_c}\left(\sum_{j=1}^{n_s}\sum_{k=1}^{m}\boldsymbol{Z}_{ijk}\right)\left(\sum_{j=1}^{n_s}\sum_{k=1}^{m}\boldsymbol{Z}_{ijk}^T\right)$$

$$= \sum_{i=1}^{n_c} m^2 \left[\begin{matrix} n_s^2 & n_s\sum_{j=1}^{n_s}(W_{ij}-\overline{W}) & \cdots \\ n_s\sum_{j=1}^{n_s}(W_{ij}-\overline{W}) & \{\sum_{j=1}^{n_s}(W_{ij}-\overline{W})\}^2 & \cdots \\ n_s\sum_{j=1}^{n_s}\overline{X}_{ij} & \sum_{j=1}^{n_s}(W_{ij}-\overline{W})\sum_{j=1}^{n_s}\overline{X}_{ij} & \cdots \\ n_s\sum_{j=1}^{n_s}(W_{ij}-\overline{W})\overline{X}_{ij} & \sum_{j=1}^{n_s}(W_{ij}-\overline{W})\sum_{j=1}^{n_s}(W_{ij}-\overline{W})\overline{X}_{ij} & \cdots \end{matrix}\right.$$

$$\left.\begin{matrix} \cdots & n_s\sum_{j=1}^{n_s}\overline{X}_{ij} & n_s\sum_{j=1}^{n_s}(W_{ij}-\overline{W})\overline{X}_{ij} \\ \cdots & \sum_{j=1}^{n_s}(W_{ij}-\overline{W})\sum_{j=1}^{n_s}\overline{X}_{ij} & \sum_{j=1}^{n_s}(W_{ij}-\overline{W})\sum_{j=1}^{n_s}(W_{ij}-\overline{W})\overline{X}_{ij} \\ \cdots & \{\sum_{j=1}^{n_s}\overline{X}_{ij}\}^2 & \sum_{j=1}^{n_s}\overline{X}_{ij}\sum_{j=1}^{n_s}(W_{ij}-\overline{W})\overline{X}_{ij} \\ \cdots & \sum_{j=1}^{n_s}\overline{X}_{ij}\sum_{j=1}^{n_s}(W_{ij}-\overline{W})\overline{X}_{ij} & \{\sum_{j=1}^{n_s}(W_{ij}-\overline{W})\overline{X}_{ij}\}^2 \end{matrix}\right].$$

Due to the subcluster-level randomization is performed within each cluster, it satisfies $\sum_{j=1}^{n_s}(W_{ij}-\overline{W})=0$, therefore,

$$\begin{aligned}
\frac{1}{n_c}\sum_{i=1}^{n_c}\left\{\sum_{j=1}^{n_s}(W_{ij}-\overline{W})\right\}^2 &\to \mathbb{E}\left[\left\{\sum_{j=1}^{n_s}(W_{ij}-\overline{W})\right\}^2\right] = 0 \\
&= \mathbb{E}\left\{\sum_{j=1}^{n_s}(W_{ij}-\overline{W})^2 + \sum_{j\neq j'}(W_{ij}-\overline{W})(W_{ij'}-\overline{W})\right\} \\
&= \mathbb{E}\left\{\sum_{j=1}^{n_s}(W_{ij}-\overline{W})^2\right\} + \mathbb{E}\left\{\sum_{j\neq j'}(W_{ij}-\overline{W})(W_{ij'}-\overline{W})\right\}
\end{aligned}$$

$$\frac{1}{n_c}\sum_{i=1}^{n_c}\left[\sum_{j=1}^{n_s}(W_{ij}-\overline{W})\sum_{j=1}^{n_s}(W_{ij}-\overline{W})\overline{X}_{ij}\right] \to \mathbb{E}\left[\sum_{j=1}^{n_s}(W_{ij}-\overline{W})\sum_{j=1}^{n_s}(W_{ij}-\overline{W})\overline{X}_{ij}\right] = 0$$

These help to return the limit

$$\boldsymbol{Q} = \lim_{n_c\to\infty} \frac{1}{n_c}\boldsymbol{Q}_{(n_c,n_s,m)} = m^2 n_s^2 \begin{bmatrix} 1 & 0 & \mu_1 & 0 \\ 0 & 0 & 0 & 0 \\ \mu_1 & 0 & \eta_2 & 0 \\ 0 & 0 & 0 & 0 \end{bmatrix}.$$

Then, it comes to $\boldsymbol{U} = c\boldsymbol{S} + d\boldsymbol{T} + e\boldsymbol{Q} = \begin{bmatrix} \boldsymbol{A} & \boldsymbol{B} \\ \boldsymbol{C} & \boldsymbol{D} \end{bmatrix}$, where

$$\begin{aligned}
\boldsymbol{A} &= \begin{bmatrix} n_s m(c + dm + e n_s m) & 0 \\ 0 & n_s m \sigma_w^2 (c + dm) \end{bmatrix}, \\
\boldsymbol{B} &= \begin{bmatrix} n_s m(c + dm + e n_s m)\mu_1 & 0 \\ 0 & n_s m \sigma_w^2 (c + dm)\mu_1 \end{bmatrix} = \mu_1 \boldsymbol{A} = \boldsymbol{C}, \\
\boldsymbol{D} &= \begin{bmatrix} n_s m(c\mu_2 + dm\tau_2 + e n_s m \eta_2) & 0 \\ 0 & n_s m \sigma_w^2 (c\mu_2 + dm\tau_2) \end{bmatrix}.
\end{aligned}$$

Since the lower-right block of $\sigma_{y|x}^2 \boldsymbol{U}^{-1}$ is $\sigma_{y|x}^2(\boldsymbol{D} - \boldsymbol{C}\boldsymbol{A}^{-1}\boldsymbol{B})^{-1} = \sigma_{y|x}^2(\boldsymbol{D} - \mu_1\boldsymbol{B})^{-1}$, its lower-right element becomes

$$\lim_{n_c\to\infty} n_c \text{var}(\widehat{b}_4) = \lim_{n_c\to\infty} n_c \text{var}(\widehat{\beta}_4) = \frac{\sigma_{y|x}^2}{n_s m \sigma_w^2 \{c(\mu_2 - \mu_1^2) + dm(\tau_2 - \mu_1^2)\}}$$

Recall the results derived based on outcome-ICCs in Part (a), we can finally obtain

$$\lim_{n_c\to\infty} n_c \text{var}(\widehat{\beta}_4) = \frac{\sigma_{y|x}^2 \lambda_1\lambda_2\lambda_3}{\sigma_w^2\sigma_x^2[n_s m \lambda_2\lambda_3 - n_s\lambda_3(\lambda_2 - \lambda_1)\{1 + (m-1)\rho_0\}]},$$

which is only related to within-subcluster covariate-ICC, ρ_0. We therefore re-organize the equation to conclude that when randomization is carried out at the subcluster level, it satisfies

$$\sigma_{4,(2)}^2 = \lim_{n_c\to\infty} n_c n_s m \text{var}(\widehat{\beta}_4) = \frac{\sigma_{y|x}^2}{\overline{W}(1-\overline{W})\sigma_x^2} \times \frac{m}{m\lambda_1^{-1} - \{1 + (m-1)\rho_0\}\left(\lambda_1^{-1} - \lambda_2^{-1}\right)}.$$

A.3 Part (c)

We finally consider the univariate case when randomization is carried out at the participant level. We proceed in the same strategies and define the collection of design points as $\boldsymbol{Z}_{ijk} = (1, (W_{ijk} - \overline{W}), X_{ijk}, (W_{ijk} - \overline{W})X_{ijk})^T$. With the same target, we derive each necessary matrix components $\boldsymbol{S}_{(n_c,n_s,m)}$, $\boldsymbol{T}_{(n_c,n_s,m)}$, $\boldsymbol{Q}_{(n_c,n_s,m)}$, and their corresponding limits.

$$\boldsymbol{S}_{(n_c,n_s,m)} = \sum_{i=1}^{n_c}\sum_{j=1}^{n_s}\sum_{k=1}^{m} \boldsymbol{Z}_{ijk}\boldsymbol{Z}_{ijk}^T$$

$$= \sum_{i=1}^{n_c}\sum_{j=1}^{n_s}\sum_{k=1}^{m} \begin{bmatrix} 1 & W_{ijk}-\overline{W} & X_{ijk} & (W_{ijk}-\overline{W})X_{ijk} \\ W_{ijk}-\overline{W} & (W_{ijk}-\overline{W})^2 & (W_{ijk}-\overline{W})X_{ijk} & (W_{ijk}-\overline{W})^2X_{ijk} \\ X_{ijk} & (W_{ijk}-\overline{W})X_{ijk} & X_{ijk}^2 & (W_{ijk}-\overline{W})X_{ijk}^2 \\ (W_{ijk}-\overline{W})X_{ijk} & (W_{ijk}-\overline{W})^2X_{ijk} & (W_{ijk}-\overline{W})X_{ijk}^2 & (W_{ijk}-\overline{W})^2X_{ijk}^2 \end{bmatrix},$$

Note that the limit matrix $\boldsymbol{S}$ will be totally the same under the three randomization scenarios.

$$\boldsymbol{T}_{(n_c,n_s,m)} = \sum_{i=1}^{n_c}\sum_{j=1}^{n_s}\left(\sum_{k=1}^{m}\boldsymbol{Z}_{ijk}\right)\left(\sum_{k=1}^{m}\boldsymbol{Z}_{ijk}^T\right)$$

$$= \sum_{i=1}^{n_c}\sum_{j=1}^{n_s} \begin{bmatrix} m^2 & m\sum_{k=1}^m(W_{ijk}-\overline{W}) & m^2\overline{X}_{ij} & m\sum_{k=1}^m(W_{ijk}-\overline{W})\overline{X}_{ij} \\ m\sum_{k=1}^m(W_{ijk}-\overline{W}) & \{\sum_{k=1}^m(W_{ijk}-\overline{W})\}^2 & m\overline{X}_{ij}\sum_{k=1}^m(W_{ijk}-\overline{W}) & \sum_{k=1}^m(W_{ijk}-\overline{W})\sum_{k=1}^m(W_{ijk}-\overline{W})X_{ijk} \\ m^2\overline{X}_{ij} & m\overline{X}_{ij}\sum_{k=1}^m(W_{ijk}-\overline{W}) & m^2\overline{X}_{ij}^2 & m\overline{X}_{ij}\sum_{k=1}^m(W_{ijk}-\overline{W})X_{ijk} \\ m\sum_{k=1}^m(W_{ijk}-\overline{W})X_{ijk} & \sum_{k=1}^m(W_{ijk}-\overline{W})\sum_{k=1}^m(W_{ijk}-\overline{W})X_{ijk} & m\overline{X}_{ij}\sum_{k=1}^m(W_{ijk}-\overline{W})X_{ijk} & \{\sum_{k=1}^m(W_{ijk}-\overline{W})X_{ijk}\}^2 \end{bmatrix},$$

Because of the participant-level randomization is conducted within each subcluster, we have $\sum_{k=1}^m(W_{ijk}-\overline{W})=0$. This will lead to many zeros in the matrix above, similar to the derivation in the subcluster-level randomization scenario. We therefore have

$$\boldsymbol{T} = \lim_{n_c\to\infty}\frac{1}{n_c}\boldsymbol{T}_n = \begin{bmatrix} n_sm^2 & 0 & n_sm^2\mu_1 & 0 \\ 0 & 0 & 0 & 0 \\ n_sm^2\mu_1 & 0 & n_sm^2\tau_2 & 0 \\ 0 & 0 & 0 & 0 \end{bmatrix},$$

where μ_1, μ_2, and τ_2 were defined before. Next, we derive

$$\boldsymbol{Q}_{(n_c,n_s,m)} = \sum_{i=1}^{n_c}\left(\sum_{j=1}^{n_s}\sum_{k=1}^{m}\boldsymbol{Z}_{ijk}\right)\left(\sum_{j=1}^{n_s}\sum_{k=1}^{m}\boldsymbol{Z}_{ijk}^T\right)$$

$$= \sum_{i=1}^{n_c} \begin{bmatrix} m^2n_s^2 & mn_s\sum_{j=1}^{n_s}\sum_{k=1}^m(W_{ijk}-\overline{W}) & m^2n_s^2\overline{X}_i & mn_s\sum_{j=1}^{n_s}\sum_{k=1}^m(W_{ijk}-\overline{W})X_{ijk} \\ mn_s\sum_{j=1}^{n_s}\sum_{k=1}^m(W_{ijk}-\overline{W}) & \{\sum_{j=1}^{n_s}\sum_{k=1}^m(W_{ijk}-\overline{W})\}^2 & mn_s\overline{X}_i\sum_{j=1}^{n_s}\sum_{k=1}^m(W_{ijk}-\overline{W}) & \sum_{j=1}^{n_s}\sum_{k=1}^m(W_{ijk}-\overline{W})\sum_{j=1}^{n_s}\sum_{k=1}^m(W_{ijk}-\overline{W})X_{ijk} \\ m^2n_s^2\overline{X}_i & mn_s\overline{X}_i\sum_{j=1}^{n_s}\sum_{k=1}^m(W_{ijk}-\overline{W}) & m^2n_s^2\overline{X}_i^2 & mn_s\overline{X}_i\sum_{j=1}^{n_s}\sum_{k=1}^m(W_{ijk}-\overline{W})X_{ijk} \\ mn_s\sum_{j=1}^{n_s}\sum_{k=1}^m(W_{ijk}-\overline{W})X_{ijk} & \sum_{j=1}^{n_s}\sum_{k=1}^m(W_{ijk}-\overline{W})\sum_{j=1}^{n_s}\sum_{k=1}^m(W_{ijk}-\overline{W})X_{ijk} & mn_s\overline{X}_i\sum_{j=1}^{n_s}\sum_{k=1}^m(W_{ijk}-\overline{W})X_{ijk} & \{\sum_{j=1}^{n_s}\sum_{k=1}^m(W_{ijk}-\overline{W})X_{ijk}\}^2 \end{bmatrix},$$

which suggests the limit that

$$\boldsymbol{Q} = \lim_{n_c \to \infty} \frac{1}{n_c} \boldsymbol{Q}_{(n_c, n_s, m)} = \begin{bmatrix} m^2 n_s^2 & 0 & m^2 n_s^2 \mu_1 & 0 \\ 0 & 0 & 0 & 0 \\ m^2 n_s^2 \mu_1 & 0 & m^2 n_s^2 \eta_2 & 0 \\ 0 & 0 & 0 & 0 \end{bmatrix}.$$

Then, similarly to the fist two scenarios, we have $\boldsymbol{U} = c\boldsymbol{S} + d\boldsymbol{T} + e\boldsymbol{Q} = \begin{bmatrix} \boldsymbol{A} & \boldsymbol{B} \\ \boldsymbol{C} & \boldsymbol{D} \end{bmatrix}$, where

$$\begin{aligned} \boldsymbol{A} &= \begin{bmatrix} n_s m(c + dm + e n_s m) & 0 \\ 0 & n_s m \sigma_w^2 c \end{bmatrix}, \\ \boldsymbol{B} &= \begin{bmatrix} n_s m(c + dm + e n_s m)\mu_1 & 0 \\ 0 & n_s m \sigma_w^2 c \mu_1 \end{bmatrix} = \mu_1 \boldsymbol{A} = \boldsymbol{C}, \\ \boldsymbol{D} &= \begin{bmatrix} n_s m(c\mu_2 + dm\tau_2 + e n_s m \eta_2) & 0 \\ 0 & n_s m \sigma_w^2 c \mu_2 \end{bmatrix}. \end{aligned}$$

By the same block matrix inversion formula, we can get

$$\lim_{n_c \to \infty} n_c \text{var}(\widehat{b}_4) = \lim_{n_c \to \infty} n_c \text{var}(\widehat{\beta}_4) = \frac{\sigma_{y|x}^2}{n_s m \sigma_w^2 c(\mu_2 - \mu_1^2)} = \frac{\sigma_{y|x}^2 \lambda_1}{n_s m \sigma_w^2 \sigma_x^2},$$

which is not related to either covariate-ICC, and only depends on the within-subcluster outcome-ICC, α_0. Finally, we summarize that when randomization is carried out at the participant level,

$$\sigma_{4,(1)}^2 = \lim_{n_c \to \infty} n_c n_s m \text{var}(\widehat{\beta}_4) = \frac{\sigma_{y|x}^2}{\overline{W}(1 - \overline{W})\sigma_x^2} \times \lambda_1.$$

A.4 Ordering statements

We next prove that the variances have a strict ordering such that $\sigma_{4,(3)}^2 \geq \sigma_{4,(2)}^2 \geq \sigma_{4,(1)}^2$. Recall that $\lambda_1 = 1 - \alpha_0$, $\lambda_2 = \lambda_1 + m(\alpha_0 - \alpha_1)$, and $\lambda_3 = \lambda_2 + n_s m \alpha_1$. Since $1 > \alpha_0 \geq \alpha_1 \geq 0$, it follows $\lambda_3 \geq \lambda_2 \geq \lambda_1 > 0$. We firstly focus on the latter inequality,

$$\sigma_{4,(2)}^2 = \frac{\sigma_{y|x}^2}{\overline{W}(1 - \overline{W})\sigma_x^2} \times \frac{m\lambda_1}{m - \{1 + (m-1)\rho_0\}\left(1 - \frac{\lambda_1}{\lambda_2}\right)}.$$

Since $1 + (m-1)\rho_0 \geq 1$ and $1 - \lambda_1/\lambda_2 \geq 0$, we have

$$\sigma_{4,(2)}^2 \geq \frac{\sigma_{y|x}^2}{\overline{W}(1 - \overline{W})\sigma_x^2} \times \frac{m\lambda_1}{m - 0} = \frac{\sigma_{y|x}^2}{\overline{W}(1 - \overline{W})\sigma_x^2} \times \lambda_1 = \sigma_{4,(1)}^2,$$

and the equality holds when $\lambda_1/\lambda_2 = 1$ or $\lambda_1 = \lambda_2$, which requires $\alpha_0 = \alpha_1 = 0$.

Next, we re-write the expressions of $\sigma^2_{4,(3)}$ and $\sigma^2_{4,(2)}$ as

$$\begin{aligned}
\sigma^2_{4,(3)} &= \frac{\sigma^2_{y|x}}{\overline{W}(1-\overline{W})\sigma^2_x} \\
&\quad \times \frac{n_s m \lambda_1}{n_s m + n_s\{1+(m-1)\rho_0\}\left(\frac{\lambda_1}{\lambda_2}-1\right) - \left[\{(n_s-1)m\rho_1 + 1 + (m-1)\rho_0\}\left(\frac{\lambda_1}{\lambda_2}-\frac{\lambda_1}{\lambda_3}\right)\right]}, \\
\sigma^2_{4,(2)} &= \frac{\sigma^2_{y|x}}{\overline{W}(1-\overline{W})\sigma^2_x} \times \frac{n_s m \lambda_1}{n_s m + n_s\{1+(m-1)\rho_0\}\left(\frac{\lambda_1}{\lambda_2}-1\right)}.
\end{aligned}$$

Since $(n_s-1)m\rho_1 + 1 + (m-1)\rho_0 \geq 1$ and $\lambda_1/\lambda_2 - \lambda_1/\lambda_3 \geq 0$, we have $\sigma^2_{4,(3)} \geq \sigma^2_{4,(2)}$, and the equality holds when $\lambda_2 = \lambda_3$, which further suggests $\alpha_1 = 0$.

Finally, it is straightforward that $\sigma^2_{4,(3)} = \sigma^2_{4,(2)} = \sigma^2_{4,(1)}$ when $\lambda_1 = \lambda_2 = \lambda_3$. Since $\lambda_1 = \lambda_2 = \lambda_3 = 1$ if and only if $\alpha_0 = \alpha_1 = 0$, we can say that the equality among the three variances holds when there is no residual clustering in a three-level design such that $\boldsymbol{R}_i = \boldsymbol{I}_{n_s m}$.

Web Appendix B Relationship between σ^2_4 and ICC parameters

B.1 Randomization at the cluster level

When randomization at the cluster level, we have

$$\sigma^2_{4,(3)} = \frac{\sigma^2_{y|x}}{\overline{W}(1-\overline{W})\sigma^2_x} \times \frac{n_s m}{\lambda_3^{-1}\zeta_3 + (n_s-1)\lambda_2^{-1}\zeta_2 + n_s(m-1)\lambda_1^{-1}\zeta_1},$$

where $\zeta_1 = 1-\rho_0$, $\zeta_2 = 1+(m-1)\rho_0 - m\rho_1$ and $\zeta_3 = 1+(m-1)\rho_0 + (n_s-1)m\rho_1$. Algebra gives

$$\sigma^2_{4,(3)} = \frac{\sigma^2_{y|x}}{\overline{W}(1-\overline{W})\sigma^2_x} \times \frac{n_s m}{\mathfrak{a}_{2,(3)} + \mathfrak{a}_{1,(3)}\rho_1 + \mathfrak{a}_{0,(3)}\rho_0},$$

where

$$\begin{aligned}
\mathfrak{a}_{2,(3)} &= \lambda_3^{-1} + (n_s-1)\lambda_2^{-1} + n_s(m-1)\lambda_1^{-1} \\
\mathfrak{a}_{1,(3)} &= (n_s-1)m(\lambda_3^{-1} - \lambda_2^{-1}) \\
\mathfrak{a}_{0,(3)} &= (m-1)\{\lambda_3^{-1} + (n_s-1)\lambda_2^{-1} - n_s\lambda_1^{-1}\}.
\end{aligned}$$

Since $\lambda_3 > \lambda_2 > \lambda_1 > 0$, we have $\mathfrak{a}_{1,(3)} < 0$. Recall $\lambda_1 = 1-\alpha_0$, $\lambda_2 = 1+(m-1)\alpha_0 - m\alpha_1$ and $\lambda_3 = 1+(m-1)\alpha_0 + (n_s-1)m\alpha_1$. Then for $\mathfrak{a}_{0,(3)}$, we have

$$\mathfrak{a}_{0,(3)} = \frac{1}{\lambda_3} + \frac{n_s-1}{\lambda_2} - \frac{n_s}{\lambda_1} < \frac{1}{\lambda_1} + \frac{n_s-1}{\lambda_1} - \frac{n_s}{\lambda_1} = 0.$$

Therefore, larger values of covariate-ICCs, ρ_0, ρ_1, are always associated with a larger $\sigma^2_{4,(3)}$ (smaller power). For the relationship between α_0, α_1 and $\sigma^2_{4,(3)}$, we have

$$\sigma^2_{4,(3)} = \frac{\sigma^2_{y|x} n_s m}{\overline{W}(1-\overline{W})\sigma^2_x} \times \Big[\zeta_3 \{1+(m-1)\alpha_0+(n_s-1)m\alpha_1\}^{-1} + (n_s-1)\zeta_2 \{1+(m-1)\alpha_0 - m\alpha_1\}^{-1} + n_s(m-1)\zeta_1(1-\alpha_0)^{-1}\Big]^{-1}.$$

Let

$$\mathfrak{b}(\alpha_0,\alpha_1) = \zeta_3 \{1+(m-1)\alpha_0+(n_s-1)m\alpha_1\}^{-1} + (n_s-1)\zeta_2 \{1+(m-1)\alpha_0 - m\alpha_1\}^{-1} + n_s(m-1)\zeta_1(1-\alpha_0)^{-1}.$$

Then the derivative of $\sigma^2_{4,(3)}$ with respect to α_0 is

$$\frac{\sigma^2_{y|x} n_s m}{\overline{W}(1-\overline{W})\sigma^2_x} \times \left[\frac{\zeta_3(m-1)}{\{1+(m-1)\alpha_0+(n_s-1)m\alpha_1\}^2} + \frac{(n_s-1)(m-1)\zeta_2}{\{1+(m-1)\alpha_0-m\alpha_1\}^2} - \frac{n_s(m-1)\zeta_1}{(1-\alpha_0)^2}\right]\mathfrak{b}(\alpha_0,\alpha_1)^{-2},$$

and the derivative of $\sigma^2_{4,(3)}$ with respect to α_1 is

$$\frac{\sigma^2_{y|x} n_s m}{\overline{W}(1-\overline{W})\sigma^2_x} \times \left[\frac{\zeta_3(n_s-1)m}{\{1+(m-1)\alpha_0+(n_s-1)m\alpha_1\}^2} - \frac{(n_s-1)m\zeta_2}{\{1+(m-1)\alpha_0-m\alpha_1\}^2}\right]\mathfrak{b}(\alpha_0,\alpha_1)^{-2}.$$

Thus, the relationship between the outcome-ICCs, α_0, α_1, with $\sigma^2_{4,(3)}$ is generally in-deterministic and numerically explored in Web Figure 1 in Web Appendix G. From the graphical exploration, Web Figure 1 shows that $\sigma^2_{4,(3)}$ is generally parabolic in α_0 and α_1 under the range of outcome-ICCs that are of general interest. This observation matches and generalizes that in Yang et al. (2020) with two-level data.

We give an example of the above relationship in the context of the STRIDE study (also see Section 4, Example 4.2, for additional contexts of the study). Suppose we have a cluster randomized trial with randomization carried out at the healthcare system level (clinics are subclusters nested in healthcare systems and participants are nested in clinics), and there is an interest in studying effect modifier by a binary covariate, self-rated health, on the continuous outcome, concern score for falling. The above results indicate that, if the degree of correlation between self-rated health of two participants coming from the same clinic (ρ_0), or from two different clinics (ρ_1) becomes larger, then the power of the treatment-by-covariate interaction test becomes smaller. This is mainly because a larger within-cluster correlation of self-rated health implies less per-participant information for estimating the interaction effect parameter. However, if the degree of correlation between the concern scores of two participants coming from the same clinic (α_0), or from two different clinics (α_1) increases (higher residual correlation), then the power of the treatment-by-covariate interaction test may first decrease and then increase. Intuitively, this relationship is not monotone because the interaction effect covariate by definition is a product of cluster-level covariate (higher residual correlation generally leads to reduced efficiency in estimating a cluster-level regression parameter)

and participant-level covariate (higher residual correlation generally leads to increased efficiency in estimating a participant-level regression parameter), and residual correlation differentially affects the "information content" for these two components underlying the interaction variable.

B.2 Randomization at the subcluster level

When randomization is carried out at the subcluster level, we have

$$\sigma^2_{4,(2)} = \frac{\sigma^2_{y|x}}{\overline{W}(1-\overline{W})\sigma^2_x} \times \frac{m}{m\lambda_1^{-1} - \{1+(m-1)\rho_0\}\left(\lambda_1^{-1} - \lambda_2^{-1}\right)}.$$

Larger values of the covariate-ICC, ρ_0, are always associated with a larger $\sigma^2_{4,(2)}$ (smaller power), because $\lambda_2^{-1} < \lambda_1^{-1}$. Define

$$\begin{aligned}\mathfrak{c}(\alpha_0,\alpha_1) = m(1-\alpha_0)^{-1} - \{1+(m-1)\rho_0\}(1-\alpha_0)^{-1} \\ + \{1+(m-1)\rho_0\}\{1+(m-1)\alpha_0 - m\alpha_1\}^{-1}.\end{aligned}$$

Then the derivative of $\sigma^2_{4,(2)}$ with respect to α_0 is

$$\frac{\sigma^2_{y|x}m}{\overline{W}(1-\overline{W})\sigma^2_x} \times \left[\frac{(m-1)(\rho_0-1)}{(1-\alpha_0)^2} + \frac{\{1+(m-1)\rho_0\}(m-1)}{\{1+(m-1)\alpha_0 - m\alpha_1\}^2}\right]\mathfrak{c}(\alpha_0,\alpha_1)^{-2}.$$

Setting the above derivative to zero and solve with respect to α_0, we will have quadratic equations on both sides of the equation, which indicates that the relationship between α_0 and $\sigma^2_{4,(2)}$ is rather complex and explored in Web Figure 2 in Web Appendix G. The derivative of $\sigma^2_{4,(2)}$ with respect to α_1 is

$$-\frac{\sigma^2_{y|x}m}{\overline{W}(1-\overline{W})\sigma^2_x} \times \frac{\{1+(m-1)\rho_0\}m}{\{1+(m-1)\alpha_0 - m\alpha_1\}^2} \times \mathfrak{c}(\alpha_0,\alpha_1)^{-2} < 0.$$

Thus, a larger value of between-subcluster outcome-ICC, α_1, is always associated with a smaller $\sigma^2_{4,(2)}$ (larger power).

We give an example of the above relationship in the context of the STRIDE study (also see Section 4, Example 4.2, for additional contexts of the study). Now suppose we have a subcluster randomized trial with randomization carried out at the clinic level (clinics are subclusters nested in healthcare systems and participants are nested in clinics), and there is an interest in studying effect modifier by a binary covariate, self-rated health, on the continuous outcome, concern score for falling. First, the power of the treatment-by-covariate interaction test is not affected at all by the degree of correlation between self-rated health of two participants coming from two different clinics (ρ_1). Intuitively, randomization at the subcluster level "randomizes out" the between-clinic covariate correlation ρ_1 and thus removes ρ_1 from the variance of the interaction effect estimator. Next, the above results indicate that, if the degree of correlation between self-rated health of two participants coming from the same clinic (ρ_0) becomes larger, then the power for the treatment-by-covariate interaction test becomes smaller. This is again because a larger within-subcluster correlation of self-rated health implies less per-participant information for estimating the interaction effect parameter. However, if the degree of correlation between the concern scores of two participants coming from

two different clinics (α_1) increases (higher residual correlation between subclusters), then the power of the interaction test increases. This is because the interaction effect parameter is now defined as a product of a subcluster-level covariate (higher residual correlation between subclusters generally leads to increased efficiency in estimating a subcluster-level regression parameter) and participant-level covariate (higher residual correlation generally leads to increased efficiency in estimating a participant-level regression parameter), and residual correlation between subclusters increases the "information content" for the two components underlying the interaction variable.

Finally, if the degree of correlation between the concern scores of two participants coming from the same clinic (α_0) increases (higher residual correlation within subclusters), then the power for the treatment-by-covariate interaction test may first decrease and then increase (non-monotonic relationship). Intuitively, this relationship is not monotone because the interaction effect covariate by definition is a product of subcluster-level covariate (higher residual correlation within subclusters generally leads to reduced efficiency in estimating a subcluster-level regression parameter) and participant-level covariate (higher residual correlation generally leads to increased efficiency in estimating a participant-level regression parameter), and residual correlation within subclusters can differentially affect the "information content" for these two components underlying the interaction variable.

B.3 Randomization at the participant level

The relationship between the different ICC parameters and the variance of the interaction effect estimator under an IRT is obvious based on the formula derived in Theorem 2.1 of the main manuscript. In the context of the STRIDE example, suppose now we have an IRT where participants are randomized. Since the randomization is conducted at the participant level, then the within-cluster correlation between self-rated health will be "randomized out" and thus the power of the treatment-by-covariate interaction test does not depend on either of the two covariate-ICCs (ρ_0 and ρ_1). Furthermore, now that the interaction variable by definition is a product of two participant-level covariates, and larger within-cluster residual correlations are expected to increase the efficiency for estimating the interaction effect parameter. Indeed, as the degree of correlation between the concern scores of two participants coming from the same clinic (α_0) increases, the power of the treatment-by-covariate interaction test increases. It happens that the power of the treatment-by-covariate interaction test does not depend on the degree of correlation between the concern scores of two participants coming from two different clinics (α_1), beyond dependence on α_0.

Web Appendix C A general version of Theorem 2.1 and its proof

C.1 A generalization of Theorem 2.1

To proceed, we define $\boldsymbol{\mu}_1 = \mathbb{E}(\boldsymbol{X}_{ijk}) = \lim_{n_c\to\infty}(n_c n_s m)^{-1}\sum_{i=1}^{n_c}\sum_{j=1}^{n_s}\sum_{k=1}^{m}\boldsymbol{X}_{ijk}$ as the mean covariate vector, and $\boldsymbol{M}_2 = \mathbb{E}(\boldsymbol{X}_{ijk}\boldsymbol{X}_{ijk}^T) = \lim_{n_c\to\infty}(n_c n_s m)^{-1}\sum_{i=1}^{n_c}\sum_{j=1}^{n_s}\sum_{k=1}^{m}\boldsymbol{X}_{ijk}\boldsymbol{X}_{ijk}^T$ as the second moment of the effect modifiers. This allows us to write $\boldsymbol{\Omega}_x = \text{diag}(\boldsymbol{M}_2 - \boldsymbol{\mu}_1\boldsymbol{\mu}_1^T) = \text{diag}(\sigma_{x_1}^2, \ldots, \sigma_{x_p}^2)$ as the diagonal matrix with the marginal variance for each effect modifier. We write $\boldsymbol{\Upsilon}_x = \boldsymbol{\Omega}_x^{-1/2}\left\{\mathbb{E}(\boldsymbol{X}_{ijk}\boldsymbol{X}_{ijk}^T) - \boldsymbol{\mu}_1\boldsymbol{\mu}_1^T\right\}\boldsymbol{\Omega}_x^{-1/2} = \boldsymbol{\Omega}_x^{-1/2}\left(\boldsymbol{M}_2 - \boldsymbol{\mu}_1\boldsymbol{\mu}_1^T\right)\boldsymbol{\Omega}_x^{-1/2}$ as the marginal correlation matrix between p effect modifiers (the diagonal elements are 1 by construction), and

define the two common covariate-ICC matrices,

$$\boldsymbol{\Gamma}_0 = \boldsymbol{\Omega}_x^{-1/2}\left\{\mathbb{E}(\boldsymbol{X}_{ijk}\boldsymbol{X}_{ijk'}^T) - \boldsymbol{\mu}_1\boldsymbol{\mu}_1^T\right\}\boldsymbol{\Omega}_x^{-1/2},\ \forall k \neq k',$$
$$\boldsymbol{\Gamma}_1 = \boldsymbol{\Omega}_x^{-1/2}\left\{\mathbb{E}(\boldsymbol{X}_{ijk}\boldsymbol{X}_{ij'k'}^T) - \boldsymbol{\mu}_1\boldsymbol{\mu}_1^T\right\}\boldsymbol{\Omega}_x^{-1/2},\ \forall j \neq j',\ k \neq k',$$

as the multivariate extensions of ρ_0 and ρ_1. In other words, each diagonal element of $\boldsymbol{\Gamma}_0$ and $\boldsymbol{\Gamma}_1$ is the within-subcluster and between-subcluster covariate-ICC, respectively. Each off-diagonal element of $\boldsymbol{\Gamma}_0$ and $\boldsymbol{\Gamma}_1$ is the within-subcluster and between-subcluster intraclass cross-correlations between two different effect modifiers. If we let $\boldsymbol{X}_i = (\boldsymbol{X}_{i11}^T, \ldots, \boldsymbol{X}_{i1m}^T, \ldots, \boldsymbol{X}_{in_s,1}^T, \ldots, \boldsymbol{X}_{in_s,m}^T)^T$, the marginal correlation matrix for $\boldsymbol{X}_i$ appears as a multivariate extension of (4) (or Equation (2.4) in the main manuscript), given by

$$\boldsymbol{L}_i = \boldsymbol{I}_{n_s m} \otimes (\boldsymbol{\Upsilon}_x - \boldsymbol{\Gamma}_0) + \boldsymbol{I}_{n_s} \otimes \boldsymbol{J}_m \otimes (\boldsymbol{\Gamma}_0 - \boldsymbol{\Gamma}_1) + \boldsymbol{J}_{n_s m} \otimes \boldsymbol{\Gamma}_1, \tag{5}$$

We call equation (5) the *nested block exchangeable correlation structure*, under which we prove the following multivariate extension of Theorem 2.1.

Theorem C.1.1 *Assuming the nested block exchangeable correlation structure for the collection of effect modifiers, the limit covariance matrix* $\boldsymbol{\Omega}_4$ *is an explicit function of the eigenvalues of outcome-ICC matrix as well as the covariate-ICC matrices.*
(a) When the randomization is carried out at the cluster level, the covariance matrix of the HTE estimator

$$\boldsymbol{\Omega}_{4,(3)} = \frac{\sigma_{y|x}^2}{\overline{W}(1-\overline{W})} \times \boldsymbol{\Omega}_x^{-1/2}\left\{\kappa_{2,(3)}\boldsymbol{\Upsilon}_x + \kappa_{1,(3)}\boldsymbol{\Gamma}_1 + \kappa_{0,(3)}\boldsymbol{\Gamma}_0\right\}^{-1}\boldsymbol{\Omega}_x^{-1/2},$$

where the linear coefficients are given by

$$\kappa_{2,(3)} = \frac{1}{\lambda_1} - \frac{\lambda_2-\lambda_1}{m\lambda_1\lambda_2} - \frac{\lambda_3-\lambda_2}{n_s m\lambda_2\lambda_3},\quad \kappa_{1,(3)} = -\frac{(n_s-1)(\lambda_3-\lambda_2)}{n_s\lambda_2\lambda_3},$$
$$\kappa_{0,(3)} = -\frac{(m-1)(\lambda_2-\lambda_1)}{m\lambda_1\lambda_2} - \frac{(m-1)(\lambda_3-\lambda_2)}{n_s m\lambda_2\lambda_3}.$$

(b) When randomization is carried out at the subcluster level, we have

$$\boldsymbol{\Omega}_{4,(2)} = \frac{\sigma_{y|x}^2}{\overline{W}(1-\overline{W})} \times \boldsymbol{\Omega}_x^{-1/2}\left\{\kappa_{2,(2)}\boldsymbol{\Upsilon}_x + \kappa_{0,(2)}\boldsymbol{\Gamma}_0\right\}^{-1}\boldsymbol{\Omega}_x^{-1/2},$$

where the linear coefficients are given by

$$\kappa_{2,(2)} = \frac{1}{\lambda_1} - \frac{\lambda_2-\lambda_1}{m\lambda_1\lambda_2},\qquad \kappa_{0,(2)} = -\frac{(m-1)(\lambda_2-\lambda_1)}{m\lambda_1\lambda_2}$$

(c) When randomization is carried out at the participant level, we have

$$\boldsymbol{\Omega}_{4,(1)} = \frac{\sigma_{y|x}^2}{\overline{W}(1-\overline{W})} \times \lambda_1\boldsymbol{\Omega}_x^{-1/2}\boldsymbol{\Upsilon}_x^{-1}\boldsymbol{\Omega}_x^{-1/2}$$

(d) The covariance matrices have Löwner ordering such that $\boldsymbol{\Omega}_{4,(3)} \succeq \boldsymbol{\Omega}_{4,(2)} \succeq \boldsymbol{\Omega}_{4,(1)}$*, with equality obtained in the absence of residual clustering (e.g.,* $\lambda_1 = \lambda_2 = \lambda_3 = 1$ *or* $\sigma_\gamma^2 = \sigma_u^2 = 0$*).*

Theorem C.1.1 provides a cascade of variance formulas for $\widehat{\boldsymbol{\beta}}_4$ to facilitate efficient sample size determination. It is clear that Theorem 2.1 in the main manuscript is a special case of Theorem C.1.1 when $p = 1$, in which case, $\boldsymbol{\Gamma}_0 = \rho_0$, $\boldsymbol{\Gamma}_1 = \rho_1$ and $\boldsymbol{\Upsilon}_x = 1$ and therefore $\boldsymbol{\Omega}_{4,(l)} = \sigma^2_{4,(l)}$ for $l \in \{1, 2, 3\}$. Therefore, the findings based on Theorem 2.1 are illustrative of those based on Theorem C.1.1. For example, the covariance matrix of $\widehat{\boldsymbol{\beta}}_4$ only depends on the covariate-ICC matrices defined within each randomization unit but not between randomization units. Specifically, $\boldsymbol{\Omega}_{4,(3)}$ depends on both $\boldsymbol{\Gamma}_1$ and $\boldsymbol{\Gamma}_0$, $\boldsymbol{\Omega}_{4,(2)}$ depends only on $\boldsymbol{\Gamma}_0$, whereas $\boldsymbol{\Omega}_{4,(1)}$ is independent of both $\boldsymbol{\Gamma}_1$ and $\boldsymbol{\Gamma}_0$. However, all three covariance matrices, $\boldsymbol{\Omega}_{4,(l)}$, depend on the marginal correlation matrix of the p effect modifiers, $\boldsymbol{\Upsilon}_x$. In Web Appendix C.6, we additionally provide simplified covariance expressions when the vector of effect modifiers is measured at the subcluster or cluster level.

C.2 Part (a)

For multivariate case when randomization is carried out at the cluster level. The reparameterized linear mixed analysis of covariance (LM-ANCOVA) model is then given by

$$Y_{ijk} = b_1 + b_2(W_{ijk} - \overline{W}) + \boldsymbol{b}_3^T \boldsymbol{X}_{ijk} + \boldsymbol{b}_4^T (W_{ijk} - \overline{W})\boldsymbol{X}_{ijk} + \gamma_i + u_{ij} + \epsilon_{ijk},$$

where $W_{ijk} = W_i$ under the current randomization scenario, $b_1 = \beta_1 + \beta_2\overline{W}$, $b_2 = \beta_2$, $\boldsymbol{b}_3 = \boldsymbol{\beta}_3 + \boldsymbol{\beta}_4\overline{W}$ and $\boldsymbol{b}_4 = \boldsymbol{\beta}_4$. Similarly, the total variance of outcome conditional on $\boldsymbol{X}_{ijk}$ is $\sigma^2_{y|x} = \sigma^2_\gamma + \sigma^2_u + \sigma^2_\epsilon$, and the within-subcluster outcome-ICC, $\alpha_0 = (\sigma^2_\gamma + \sigma^2_u)/\sigma^2_{y|x}$, with the between-subcluster outcome-ICC, $\alpha_1 = \sigma^2_\gamma/\sigma^2_{y|x}$.

The correlation structure for $\mathbf{Y}_i = (Y_{i11}, \ldots, Y_{i1m}, \ldots, Y_{in_s,1}, \ldots, Y_{1n_s,m})^T$ is nested exchangeable with matrix expression given as

$$\boldsymbol{R}_i = (1 - \alpha_0)\boldsymbol{I}_{n_s m} + (\alpha_0 - \alpha_1)\boldsymbol{I}_{n_s} \otimes \boldsymbol{J}_m + \alpha_1 \boldsymbol{J}_{n_s m},$$

where '$\otimes$', $\boldsymbol{I}_d$, and $\boldsymbol{J}_d$ are the same as those defined in the univariate case. Define the collection of design points $\boldsymbol{Z}_{ijk} = (1, (W_i - \overline{W}), \boldsymbol{X}^T_{ijk}, (W_i - \overline{W})\boldsymbol{X}^T_{ijk})^T$, and $\boldsymbol{Z}_i = (\boldsymbol{Z}_{i11}, \ldots, \boldsymbol{Z}_{in_s,m})^T$ as the design matrix for cluster i. Given values of the variance components, the best linear unbiased estimator of $\boldsymbol{b} = (b_1, b_2, \boldsymbol{b}_3^T, \boldsymbol{b}_4^T)^T$ is the Generalized Least Squares (GLS) estimator, given by $\widehat{\boldsymbol{b}} = \left(\sum_{i=1}^{n_c} \boldsymbol{Z}_i^T \boldsymbol{R}_i^{-1} \boldsymbol{Z}_i\right)^{-1} \left(\sum_{i=1}^{n_c} \boldsymbol{Z}_i^T \boldsymbol{R}_i^{-1} \mathbf{Y}_i\right)$.

As discussed previously, deriving the large-sample variance of $\sqrt{n_c}\widehat{\boldsymbol{b}}$ is equivalent to identifying the explicit expression of the limit variance matrix $\boldsymbol{\Sigma}_{(\infty, n_s, m)} = \sigma^2_{y|x}\boldsymbol{U}^{-1} = \sigma^2_{y|x}\left(\lim_{n_c\to\infty} n_c^{-1}\boldsymbol{U}_{(n_c,n_s,m)}\right)^{-1}$, where $\boldsymbol{U}_{(n_c,n_s,m)} = \sum_{i=1}^{n_c} \boldsymbol{Z}_i^T \boldsymbol{R}_i^{-1} \boldsymbol{Z}_i$. Specifically, $\boldsymbol{\Omega}_{4,(3)} = \lim_{n_c\to\infty} n_c n_s m \text{cov}(\widehat{\boldsymbol{b}}_4)$ and $\lim_{n_c\to\infty} n_c \text{cov}(\widehat{\boldsymbol{b}}_4)$ is the lower-right $p \times p$ block of $\boldsymbol{\Sigma}_{(\infty,n_s,m)}$.

Define $\lambda_1 = 1 - \alpha_0$, $\lambda_2 = 1 + (m-1)\alpha_0 - m\alpha_1$, and $\lambda_3 = 1 + (m-1)\alpha_0 + (n_s - 1)m\alpha_1$, then adopting the results in Li et al. (2018), we have

$$\boldsymbol{R}_i^{-1} = \frac{1}{\lambda_1}\boldsymbol{I}_{n_s m} - \frac{\lambda_2 - \lambda_1}{m\lambda_1\lambda_2}\boldsymbol{I}_{n_s} \otimes \boldsymbol{J}_m - \frac{\lambda_3 - \lambda_2}{n_s m \lambda_2 \lambda_3}\boldsymbol{J}_{n_s m} = c\boldsymbol{I}_{n_s m} + d\boldsymbol{I}_{n_s} \otimes \boldsymbol{J}_m + e\boldsymbol{J}_{n_s m},$$

where $c = 1/\lambda_1$, $d = -(\lambda_2 - \lambda_1)/(m\lambda_1\lambda_2)$, and $e = -(\lambda_3 - \lambda_2)/(n_s m \lambda_2\lambda_3)$. We further define $\overline{\boldsymbol{X}}_{ij} = m^{-1}\sum_{k=1}^m \boldsymbol{X}_{ijk}$ as the mean of covariate for each subcluster, and $\overline{\boldsymbol{X}}_i = (n_s m)^{-1}\sum_{j=1}^{n_s}\sum_{k=1}^m \boldsymbol{X}_{ijk}$

as the mean of covariate for each cluster, and based on the form of $\boldsymbol{R}_i^{-1}$, we can write $\boldsymbol{U}_{(n_c,n_s,m)} = c\boldsymbol{S}_{(n_c,n_s,m)} + d\boldsymbol{T}_{(n_c,n_s,m)} + e\boldsymbol{Q}_{(n_c,n_s,m)}$, where

$$\begin{aligned}
\boldsymbol{S}_{(n_c,n_s,m)} &= \sum_{i=1}^{n_c}\sum_{j=1}^{n_s}\sum_{k=1}^{m} \boldsymbol{Z}_{ijk}\boldsymbol{Z}_{ijk}^T \\
&= \sum_{i=1}^{n_c}\sum_{j=1}^{n_s}\sum_{k=1}^{m} \begin{bmatrix} 1 & W_i-\overline{W} & \boldsymbol{X}_{ijk}^T & (W_i-\overline{W})\boldsymbol{X}_{ijk}^T \\ W_i-\overline{W} & (W_i-\overline{W})^2 & (W_i-\overline{W})\boldsymbol{X}_{ijk}^T & (W_i-\overline{W})^2\boldsymbol{X}_{ijk}^T \\ \boldsymbol{X}_{ijk} & (W_i-\overline{W})\boldsymbol{X}_{ijk} & \boldsymbol{X}_{ijk}\boldsymbol{X}_{ijk}^T & (W_i-\overline{W})\boldsymbol{X}_{ijk}\boldsymbol{X}_{ijk}^T \\ (W_i-\overline{W})\boldsymbol{X}_{ijk} & (W_i-\overline{W})^2\boldsymbol{X}_{ijk} & (W_i-\overline{W})\boldsymbol{X}_{ijk}\boldsymbol{X}_{ijk}^T & (W_i-\overline{W})^2\boldsymbol{X}_{ijk}\boldsymbol{X}_{ijk}^T \end{bmatrix}, \\
\boldsymbol{T}_{(n_c,n_s,m)} &= \sum_{i=1}^{n_c}\sum_{j=1}^{n_s}\left(\sum_{k=1}^{m}\boldsymbol{Z}_{ijk}\right)\left(\sum_{k=1}^{m}\boldsymbol{Z}_{ijk}^T\right) \\
&= \sum_{i=1}^{n_c}\sum_{j=1}^{n_s} m^2 \begin{bmatrix} 1 & W_i-\overline{W} & \overline{\boldsymbol{X}}_{ij}^T & (W_i-\overline{W})\overline{\boldsymbol{X}}_{ij}^T \\ W_i-\overline{W} & (W_i-\overline{W})^2 & (W_i-\overline{W})\overline{\boldsymbol{X}}_{ij}^T & (W_i-\overline{W})^2\overline{\boldsymbol{X}}_{ij}^T \\ \overline{\boldsymbol{X}}_{ij} & (W_i-\overline{W})\overline{\boldsymbol{X}}_{ij} & \overline{\boldsymbol{X}}_{ij}\overline{\boldsymbol{X}}_{ij}^T & (W_i-\overline{W})\overline{\boldsymbol{X}}_{ij}\overline{\boldsymbol{X}}_{ij}^T \\ (W_i-\overline{W})\overline{\boldsymbol{X}}_{ij} & (W_i-\overline{W})^2\overline{\boldsymbol{X}}_{ij} & (W_i-\overline{W})\overline{\boldsymbol{X}}_{ij}\overline{\boldsymbol{X}}_{ij}^T & (W_i-\overline{W})^2\overline{\boldsymbol{X}}_{ij}\overline{\boldsymbol{X}}_{ij}^T \end{bmatrix}, \\
\boldsymbol{Q}_{(n_c,n_s,m)} &= \sum_{i=1}^{n_c}\left(\sum_{j=1}^{n_s}\sum_{k=1}^{m}\boldsymbol{Z}_{ijk}\right)\left(\sum_{j=1}^{n_s}\sum_{k=1}^{m}\boldsymbol{Z}_{ijk}^T\right) \\
&= \sum_{i=1}^{n_c}(n_s m)^2 \begin{bmatrix} 1 & W_i-\overline{W} & \overline{\boldsymbol{X}}_{i}^T & (W_i-\overline{W})\overline{\boldsymbol{X}}_{i}^T \\ W_i-\overline{W} & (W_i-\overline{W})^2 & (W_i-\overline{W})\overline{\boldsymbol{X}}_{i}^T & (W_i-\overline{W})^2\overline{\boldsymbol{X}}_{i}^T \\ \overline{\boldsymbol{X}}_{i} & (W_i-\overline{W})\overline{\boldsymbol{X}}_{i} & \overline{\boldsymbol{X}}_{i}\overline{\boldsymbol{X}}_{i}^T & (W_i-\overline{W})\overline{\boldsymbol{X}}_{i}\overline{\boldsymbol{X}}_{i}^T \\ (W_i-\overline{W})\overline{\boldsymbol{X}}_{i} & (W_i-\overline{W})^2\overline{\boldsymbol{X}}_{i} & (W_i-\overline{W})\overline{\boldsymbol{X}}_{i}\overline{\boldsymbol{X}}_{i}^T & (W_i-\overline{W})^2\overline{\boldsymbol{X}}_{i}\overline{\boldsymbol{X}}_{i}^T \end{bmatrix}.
\end{aligned}$$

To simplify the above matrices, we further define population-level parameters $\boldsymbol{\mu}_1 = \lim_{n_c\to\infty}(n_c n_s m)^{-1} \sum_{i=1}^{n_c}\sum_{j=1}^{n_s}\sum_{k=1}^{m}\boldsymbol{X}_{ijk}^{\otimes r}$ and $\boldsymbol{M}_2 = \lim_{n_c\to\infty}(n_c n_s m)^{-1} \sum_{i=1}^{n_c}\sum_{j=1}^{n_s}\sum_{k=1}^{m}\boldsymbol{X}_{ijk}\boldsymbol{X}_{ijk}^T$. Also define $\boldsymbol{\Upsilon}_2 = \lim_{n_c\to\infty}(n_c n_s)^{-1}\sum_{i=1}^{n_c}\sum_{j=1}^{n_s}\overline{\boldsymbol{X}}_{ij}\overline{\boldsymbol{X}}_{ij}^T$, and $\boldsymbol{H}_2 = \lim_{n_c\to\infty} n_c^{-1}\sum_{i=1}^{n_c}\overline{\boldsymbol{X}}_i\overline{\boldsymbol{X}}_i^T$. Denote $\sigma_w^2 = \mathrm{var}(W_i)$, we can obtain the following limits

$$\begin{aligned}
\boldsymbol{S} &= \lim_{n_c\to\infty}\frac{1}{n_c}\boldsymbol{S}_{(n_c,n_s,m)} = n_s m \begin{bmatrix} 1 & 0 & \boldsymbol{\mu}_1^T & \mathbf{0}_{p\times1}^T \\ 0 & \sigma_w^2 & \mathbf{0}_{p\times1}^T & \sigma_w^2\boldsymbol{\mu}_1^T \\ \boldsymbol{\mu}_1 & \mathbf{0}_{p\times1} & \boldsymbol{M}_2 & \mathbf{0}_{p\times p} \\ \mathbf{0}_{p\times1} & \sigma_w^2\boldsymbol{\mu}_1 & \mathbf{0}_{p\times p} & \sigma_w^2\boldsymbol{M}_2 \end{bmatrix}, \\
\boldsymbol{T} &= \lim_{n_c\to\infty}\frac{1}{n_c}\boldsymbol{T}_{(n_c,n_s,m)} = n_s m^2 \begin{bmatrix} 1 & 0 & \boldsymbol{\mu}_1^T & \mathbf{0}_{p\times1}^T \\ 0 & \sigma_w^2 & \mathbf{0}_{p\times1}^T & \sigma_w^2\boldsymbol{\mu}_1^T \\ \boldsymbol{\mu}_1 & \mathbf{0}_{p\times1} & \boldsymbol{\Upsilon}_2 & \mathbf{0}_{p\times p} \\ \mathbf{0}_{p\times1} & \sigma_w^2\boldsymbol{\mu}_1 & \mathbf{0}_{p\times p} & \sigma_w^2\boldsymbol{\Upsilon}_2 \end{bmatrix}, \\
\boldsymbol{Q} &= \lim_{n_c\to\infty}\frac{1}{n_c}\boldsymbol{Q}_{(n_c,n_s,m)} = n_s^2 m^2 \begin{bmatrix} 1 & 0 & \boldsymbol{\mu}_1^T & \mathbf{0}_{p\times1}^T \\ 0 & \sigma_w^2 & \mathbf{0}_{p\times1}^T & \sigma_w^2\boldsymbol{\mu}_1^T \\ \boldsymbol{\mu}_1 & \mathbf{0}_{p\times1} & \boldsymbol{H}_2 & \mathbf{0}_{p\times p} \\ \mathbf{0}_{p\times1} & \sigma_w^2\boldsymbol{\mu}_1 & \mathbf{0}_{p\times p} & \sigma_w^2\boldsymbol{H}_2 \end{bmatrix}.
\end{aligned}$$

Then, we write $\boldsymbol{U}$ in four blocks such that $\boldsymbol{U} = c\boldsymbol{S} + d\boldsymbol{T} + e\boldsymbol{Q} = \begin{bmatrix} \boldsymbol{A} & \boldsymbol{B} \\ \boldsymbol{C} & \boldsymbol{D} \end{bmatrix}$, where $\boldsymbol{C} = \boldsymbol{B}^T$ and

$$\begin{aligned}
\boldsymbol{A} &= n_s m(c + dm + en_s m) \begin{bmatrix} 1 & 0 \\ 0 & \sigma_w^2 \end{bmatrix}, \\
\boldsymbol{B} &= n_s m(c + dm + en_s m) \begin{bmatrix} \boldsymbol{\mu}_1^T & \boldsymbol{0}_{p\times 1}^T \\ \boldsymbol{0}_{p\times 1}^T & \sigma_w^2 \boldsymbol{\mu}_1^T \end{bmatrix} = \boldsymbol{A} \otimes \boldsymbol{\mu}_1^T, \\
\boldsymbol{D} &= n_s m \begin{bmatrix} (c\boldsymbol{M}_2 + dm\boldsymbol{\Upsilon}_2 + en_s m\boldsymbol{H}_2) & \boldsymbol{0}_{p\times p} \\ \boldsymbol{0}_{p\times p} & \sigma_w^2 (c\boldsymbol{M}_2 + dm\Upsilon_2 + en_s m\boldsymbol{H}_2) \end{bmatrix}.
\end{aligned}$$

Based on the block matrix inversion formula, we can derive the explicit form of the lower-right block in $\boldsymbol{U}^{-1}$, and therefore, the desired lower-right $p \times p$ block of $\boldsymbol{\Sigma}_{(\infty, n_s, m)} = \sigma_{y|x}^2 \boldsymbol{U}^{-1}$. Notice that each block matrix in $\boldsymbol{U}$ is now a diagonal matrix, which makes the derivation simple. Specifically, the lower-right block of $\boldsymbol{\Sigma}_{(\infty, n_s, m)}$ is $\sigma_{y|x}^2 (\boldsymbol{D} - \boldsymbol{C}\boldsymbol{A}^{-1}\boldsymbol{B})^{-1}$, and its lower-right $p \times p$ block is

$$\begin{aligned}
\lim_{n_c \to \infty} n_c \text{cov}(\widehat{\boldsymbol{b}}_4) &= \frac{\sigma_{y|x}^2}{n_s m \sigma_w^2} \left\{ c(\boldsymbol{M}_2 - \boldsymbol{\mu}_1\boldsymbol{\mu}_1^T) + dm(\boldsymbol{\Upsilon}_2 - \boldsymbol{\mu}_1\boldsymbol{\mu}_1^T) + en_s m(\boldsymbol{H}_2 - \boldsymbol{\mu}_1\boldsymbol{\mu}_1^T) \right\}^{-1} \\
&= \frac{\sigma_{y|x}^2}{n_s m \sigma_w^2} \left\{ \frac{1}{\lambda_1}(\boldsymbol{M}_2 - \boldsymbol{\mu}_1\boldsymbol{\mu}_1^T) - \frac{\lambda_2 - \lambda_1}{\lambda_1\lambda_2}(\boldsymbol{\Upsilon}_2 - \boldsymbol{\mu}_1\boldsymbol{\mu}_1^T) - \frac{\lambda_3 - \lambda_2}{\lambda_2\lambda_3}(\boldsymbol{H}_2 - \boldsymbol{\mu}_1\boldsymbol{\mu}_1^T) \right\}^{-1}
\end{aligned} \tag{6}$$

Define

$$\begin{aligned}
&\boldsymbol{\Gamma}_0 = \boldsymbol{\Omega}_x^{-1/2} \left\{ \mathbb{E}(\boldsymbol{X}_{ijk}\boldsymbol{X}_{ijk'}^T) - \boldsymbol{\mu}_1\boldsymbol{\mu}_1^T \right\} \boldsymbol{\Omega}_x^{-1/2}, \ \forall k \neq k', \\
&\boldsymbol{\Gamma}_1 = \boldsymbol{\Omega}_x^{-1/2} \left\{ \mathbb{E}(\boldsymbol{X}_{ijk}\boldsymbol{X}_{ij'k'}^T) - \boldsymbol{\mu}_1\boldsymbol{\mu}_1^T \right\} \boldsymbol{\Omega}_x^{-1/2}, \ \forall j \neq j', \ k \neq k', \\
&\boldsymbol{\Upsilon}_x = \boldsymbol{\Omega}_x^{-1/2} \left\{ \mathbb{E}(\boldsymbol{X}_{ijk}\boldsymbol{X}_{ijk}^T) - \boldsymbol{\mu}_1\boldsymbol{\mu}_1^T \right\} \boldsymbol{\Omega}_x^{-1/2} = \boldsymbol{\Omega}_x^{-1/2} \left(\boldsymbol{M}_2 - \boldsymbol{\mu}_1\boldsymbol{\mu}_1^T \right) \boldsymbol{\Omega}_x^{-1/2},
\end{aligned}$$

where $\boldsymbol{\Omega}_x = \text{diag}(\boldsymbol{M}_2 - \boldsymbol{\mu}_1\boldsymbol{\mu}_1^T)$. Among the above three matrices, $\boldsymbol{\Upsilon}_x$ is the marginal correlation matrix between p effect modifiers (the diagonal elements are 1 by construction), $\boldsymbol{\Gamma}_0$ and $\boldsymbol{\Gamma}_1$ are multivariate extensions of ρ_0 and ρ_1, respectively. The diagonal element of $\boldsymbol{\Gamma}_0$ and $\boldsymbol{\Gamma}_1$ are the covariate-ICC's of each covariate, and off-diagonal elements are the covariate-ICC's between different covariates. Observe that

$$\begin{aligned}
\boldsymbol{H}_2 &= \lim_{n_c \to \infty} \frac{1}{n_c (n_s m)^2} \sum_{i=1}^{n_c} \left(\sum_{j=1}^{n_s} \sum_{k=1}^{m} \boldsymbol{X}_{ijk} \right) \left(\sum_{j=1}^{n_s} \sum_{k=1}^{m} \boldsymbol{X}_{ijk}^T \right) \\
&= \lim_{n_c \to \infty} \frac{1}{n_c (n_s m)^2} \sum_{i=1}^{n_c} \left(\sum_{j=1}^{n_s} \sum_{k=1}^{m} \boldsymbol{X}_{ijk}\boldsymbol{X}_{ijk}^T + \sum_{j=1}^{n_s} \sum_{k \neq k'} \boldsymbol{X}_{ijk}\boldsymbol{X}_{ijk'}^T + \sum_{j \neq j'} \sum_{k \neq k'} \boldsymbol{X}_{ijk}\boldsymbol{X}_{ij'k'}^T \right) \\
&= \frac{1}{n_s m} \boldsymbol{M}_2 + \frac{n_c n_s m(m-1)}{n_c (n_s m)^2} \left\{ \boldsymbol{\mu}_1\boldsymbol{\mu}_1^T + \boldsymbol{\Omega}_x^{1/2} \boldsymbol{\Gamma}_0 \boldsymbol{\Omega}_x^{1/2} \right\} + \frac{n_c n_s (n_s - 1) m^2}{n_c (n_s m)^2} \left\{ \boldsymbol{\mu}_1\boldsymbol{\mu}_1^T + \boldsymbol{\Omega}_x^{1/2} \boldsymbol{\Gamma}_1 \boldsymbol{\Omega}_x^{1/2} \right\} \\
&= \frac{1}{n_s m} \left\{ \boldsymbol{M}_2 - \boldsymbol{\mu}_1\boldsymbol{\mu}_1^T + mn_s \boldsymbol{\mu}_1\boldsymbol{\mu}_1^T + (m-1)\boldsymbol{\Omega}_x^{1/2} \boldsymbol{\Gamma}_0 \boldsymbol{\Omega}_x^{1/2} + m(n_s - 1)\boldsymbol{\Omega}_x^{1/2} \boldsymbol{\Gamma}_1 \boldsymbol{\Omega}_x^{1/2} \right\}.
\end{aligned}$$

This gives

$$\boldsymbol{H}_2 - \boldsymbol{\mu}_1\boldsymbol{\mu}_1^T = \frac{1}{n_s m}\left\{\boldsymbol{M}_2 - \boldsymbol{\mu}_1\boldsymbol{\mu}_1^T + (m-1)\boldsymbol{\Omega}_x^{1/2}\boldsymbol{\Gamma}_0\boldsymbol{\Omega}_x^{1/2} + m(n_s-1)\boldsymbol{\Omega}_x^{1/2}\boldsymbol{\Gamma}_1\boldsymbol{\Omega}_x^{1/2}\right\}.$$

Similarly, we derive

$$\begin{aligned}\boldsymbol{\Upsilon}_2 &= \lim_{n_c\to\infty}\frac{1}{n_c n_s m^2}\sum_{i=1}^{n_c}\sum_{j=1}^{n_s}\left(\sum_{k=1}^{m}\boldsymbol{X}_{ijk}\boldsymbol{X}_{ijk}^T + \sum_{k\neq k'}\boldsymbol{X}_{ijk}\boldsymbol{X}_{ijk'}^T\right)\\ &= \frac{1}{m}\boldsymbol{M}_2 + \frac{m-1}{m}\left\{\boldsymbol{\mu}_1\boldsymbol{\mu}^T + \boldsymbol{\Omega}_x^{1/2}\boldsymbol{\Gamma}_0\boldsymbol{\Omega}_x^{1/2}\right\},\end{aligned}$$

which allows us to write

$$\boldsymbol{\Upsilon}_2 - \boldsymbol{\mu}_1\boldsymbol{\mu}_1^T = \frac{1}{m}\left\{\boldsymbol{M}_2 - \boldsymbol{\mu}_1\boldsymbol{\mu}_1^T + (m-1)\boldsymbol{\Omega}_x^{1/2}\boldsymbol{\Gamma}_0\boldsymbol{\Omega}_x^{1/2}\right\}.$$

Plugging all results above into the large-sample variance expression (6), we can obtain

$$\begin{aligned}\lim_{n_c\to\infty} n_c\text{cov}(\widehat{\boldsymbol{b}}_4) &= \lim_{n_c\to\infty} n_c\text{cov}(\widehat{\boldsymbol{\beta}}_4)\\ &= \frac{\sigma_{y|x}^2}{n_s m\sigma_w^2}\left\{\frac{1}{\lambda_1}(\boldsymbol{M}_2 - \boldsymbol{\mu}_1\boldsymbol{\mu}_1^T) - \frac{\lambda_2-\lambda_1}{\lambda_1\lambda_2}(\boldsymbol{\Upsilon}_2 - \boldsymbol{\mu}_1\boldsymbol{\mu}_1^T) - \frac{\lambda_3-\lambda_2}{\lambda_2\lambda_3}(\boldsymbol{H}_2 - \boldsymbol{\mu}_1\boldsymbol{\mu}_1^T)\right\}^{-1}\\ &= \frac{\sigma_{y|x}^2}{n_s m\sigma_w^2}\boldsymbol{\Omega}_x^{-1/2}\left\{\kappa_{2,(3)}\boldsymbol{\Upsilon}_x + \kappa_{1,(3)}\boldsymbol{\Gamma}_1 + \kappa_{0,(3)}\boldsymbol{\Gamma}_0\right\}^{-1}\boldsymbol{\Omega}_x^{-1/2},\end{aligned}$$

where

$$\begin{aligned}\kappa_{2,(3)} &= \frac{1}{\lambda_1} - \frac{\lambda_2-\lambda_1}{m\lambda_1\lambda_2} - \frac{\lambda_3-\lambda_2}{n_s m\lambda_2\lambda_3}, \quad \kappa_{1,(3)} = -\frac{\lambda_3-\lambda_2}{\lambda_2\lambda_3}\times\frac{n_s-1}{n_s},\\ \kappa_{0,(3)} &= -\frac{\lambda_2-\lambda_1}{\lambda_1\lambda_2}\times\frac{m-1}{m} - \frac{\lambda_3-\lambda_2}{\lambda_2\lambda_3}\times\frac{m-1}{n_s m}.\end{aligned}$$

We can thus conclude that

$$\boldsymbol{\Omega}_{4,(3)} = \lim_{n_c\to\infty} n_c n_s m\text{cov}(\widehat{\boldsymbol{\beta}}_4) = \frac{\sigma_{y|x}^2}{\overline{W}(1-\overline{W})}\boldsymbol{\Omega}_x^{-1/2}\left\{\kappa_{2,(3)}\boldsymbol{\Upsilon}_x + \kappa_{1,(3)}\boldsymbol{\Gamma}_1 + \kappa_{0,(3)}\boldsymbol{\Gamma}_0\right\}^{-1}\boldsymbol{\Omega}_x^{-1/2},$$

which reaches the asymptotic variance when the randomization is carried out at the cluster level.

C.3 Part (b)

We then consider the univariate case when randomization is carried out at the subcluster level, where $W_{ijk} = W_{ij}$. Using the same reparametrization and procedures, we aim at the lower-right element of the limit matrix $\sigma_{y|x}^2\boldsymbol{U}^{-1} = \sigma_{y|x}^2(\lim_{n_c\to\infty} n_c^{-1}\boldsymbol{U}_{(n_c,n_s,m)})^{-1}$, where $\boldsymbol{U}_{(n_c,n_s,m)} = \sum_{i=1}^{n_c}\boldsymbol{Z}_i^T\boldsymbol{R}_i^{-1}\boldsymbol{Z}_i$. Define the collection of design points as $\boldsymbol{Z}_{ijk} = (1, (W_{ij}-\overline{W}), \boldsymbol{X}_{ijk}^T, (W_{ij}-\overline{W})\boldsymbol{X}_{ijk}^T)^T$, we pursue the component matrices referred in $\boldsymbol{U}_{(n_c,n_s,m)} = c\boldsymbol{S}_{(n_c,n_s,m)} + d\boldsymbol{T}_{(n_c,n_s,m)} + e\boldsymbol{Q}_{(n_c,n_s,m)}$ and $\boldsymbol{U} =$

$c\boldsymbol{S} + d\boldsymbol{T} + e\boldsymbol{Q}$.

$$\begin{aligned}
\boldsymbol{S}_{(n_c,n_s,m)} &= \sum_{i=1}^{n_c}\sum_{j=1}^{n_s}\sum_{k=1}^{m} \boldsymbol{Z}_{ijk}\boldsymbol{Z}_{ijk}^T \\
&= \sum_{i=1}^{n_c}\sum_{j=1}^{n_s}\sum_{k=1}^{m} \begin{bmatrix} 1 & W_{ij}-\overline{W} & \boldsymbol{X}_{ijk}^T & (W_{ij}-\overline{W})\boldsymbol{X}_{ijk}^T \\ W_{ij}-\overline{W} & (W_{ij}-\overline{W})^2 & (W_{ij}-\overline{W})\boldsymbol{X}_{ijk}^T & (W_{ij}-\overline{W})^2\boldsymbol{X}_{ijk}^T \\ \boldsymbol{X}_{ijk} & (W_{ij}-\overline{W})\boldsymbol{X}_{ijk} & \boldsymbol{X}_{ijk}\boldsymbol{X}_{ijk}^T & (W_{ij}-\overline{W})\boldsymbol{X}_{ijk}\boldsymbol{X}_{ijk}^T \\ (W_{ij}-\overline{W})\boldsymbol{X}_{ijk} & (W_{ij}-\overline{W})^2\boldsymbol{X}_{ijk} & (W_{ij}-\overline{W})\boldsymbol{X}_{ijk}\boldsymbol{X}_{ijk}^T & (W_{ij}-\overline{W})^2\boldsymbol{X}_{ijk}\boldsymbol{X}_{ijk}^T \end{bmatrix},
\end{aligned}$$

which implies an $\boldsymbol{S}$ matrix that is identical to what is presented in Part (a). Actually, we find that the limit $\boldsymbol{S}$ will be the same no matter at which level the randomization is carried out. We further express

$$\begin{aligned}
\boldsymbol{T}_{(n_c,n_s,m)} &= \sum_{i=1}^{n_c}\sum_{j=1}^{n_s}\left(\sum_{k=1}^{m}\boldsymbol{Z}_{ijk}\right)\left(\sum_{k=1}^{m}\boldsymbol{Z}_{ijk}^T\right) \\
&= \sum_{i=1}^{n_c}\sum_{j=1}^{n_s} m^2 \begin{bmatrix} 1 & W_{ij}-\overline{W} & \overline{\boldsymbol{X}}_{ij}^T & (W_{ij}-\overline{W})\overline{\boldsymbol{X}}_{ij}^T \\ W_{ij}-\overline{W} & (W_{ij}-\overline{W})^2 & (W_{ij}-\overline{W})\overline{\boldsymbol{X}}_{ij}^T & (W_{ij}-\overline{W})^2\overline{\boldsymbol{X}}_{ij}^T \\ \overline{\boldsymbol{X}}_{ij} & (W_{ij}-\overline{W})\overline{\boldsymbol{X}}_{ij} & \overline{\boldsymbol{X}}_{ij}\overline{\boldsymbol{X}}_{ij}^T & (W_{ij}-\overline{W})\overline{\boldsymbol{X}}_{ij}\overline{\boldsymbol{X}}_{ij}^T \\ (W_{ij}-\overline{W})\overline{\boldsymbol{X}}_{ij} & (W_{ij}-\overline{W})^2\overline{\boldsymbol{X}}_{ij} & (W_{ij}-\overline{W})\overline{\boldsymbol{X}}_{ij}\overline{\boldsymbol{X}}_{ij}^T & (W_{ij}-\overline{W})^2\overline{\boldsymbol{X}}_{ij}\overline{\boldsymbol{X}}_{ij}^T \end{bmatrix}.
\end{aligned}$$

We find that the correponding limit, $\boldsymbol{T}$, will also has the same form as in the cluster-level randomization case in Part (a). And, we finally express

$$\begin{aligned}
\boldsymbol{Q}_{(n_c,n_s,m)} &= \sum_{i=1}^{n_c}\left(\sum_{j=1}^{n_s}\sum_{k=1}^{m}\boldsymbol{Z}_{ijk}\right)\left(\sum_{j=1}^{n_s}\sum_{k=1}^{m}\boldsymbol{Z}_{ijk}^T\right) \\
&= \sum_{i=1}^{n_c} m^2 \left[\begin{matrix} n_s^2 & n_s\sum_{j=1}^{n_s}(W_{ij}-\overline{W}) & \cdots \\ n_s\sum_{j=1}^{n_s}(W_{ij}-\overline{W}) & \{\sum_{j=1}^{n_s}(W_{ij}-\overline{W})\}^2 & \cdots \\ n_s\sum_{j=1}^{n_s}\overline{\boldsymbol{X}}_{ij} & \sum_{j=1}^{n_s}(W_{ij}-\overline{W})\sum_{j=1}^{n_s}\overline{\boldsymbol{X}}_{ij} & \cdots \\ n_s\sum_{j=1}^{n_s}(W_{ij}-\overline{W})\overline{\boldsymbol{X}}_{ij} & \sum_{j=1}^{n_s}(W_{ij}-\overline{W})\sum_{j=1}^{n_s}(W_{ij}-\overline{W})\overline{\boldsymbol{X}}_{ij} & \cdots \end{matrix}\right. \\
&\qquad \left.\begin{matrix} \cdots & n_s\sum_{j=1}^{n_s}\overline{\boldsymbol{X}}_{ij}^T & n_s\sum_{j=1}^{n_s}(W_{ij}-\overline{W})\overline{\boldsymbol{X}}_{ij}^T \\ \cdots & \sum_{j=1}^{n_s}(W_{ij}-\overline{W})\sum_{j=1}^{n_s}\overline{\boldsymbol{X}}_{ij}^T & \sum_{j=1}^{n_s}(W_{ij}-\overline{W})\sum_{j=1}^{n_s}(W_{ij}-\overline{W})\overline{\boldsymbol{X}}_{ij}^T \\ \cdots & \sum_{j=1}^{n_s}\overline{\boldsymbol{X}}_{ij}\sum_{j=1}^{n_s}\overline{\boldsymbol{X}}_{ij}^T & \sum_{j=1}^{n_s}\overline{\boldsymbol{X}}_{ij}\sum_{j=1}^{n_s}(W_{ij}-\overline{W})\overline{\boldsymbol{X}}_{ij}^T \\ \cdots & \sum_{j=1}^{n_s}\overline{\boldsymbol{X}}_{ij}\sum_{j=1}^{n_s}(W_{ij}-\overline{W})\overline{\boldsymbol{X}}_{ij}^T & \sum_{j=1}^{n_s}(W_{ij}-\overline{W})\overline{\boldsymbol{X}}_{ij}\sum_{j=1}^{n_s}(W_{ij}-\overline{W})\overline{\boldsymbol{X}}_{ij}^T \end{matrix}\right].
\end{aligned}$$

Similar to the univariate case, due to the subcluster-level randomization is performed within each cluster, it satisfies $\sum_{j=1}^{n_s}(W_{ij}-\overline{W})=0$, therefore,

$$
\begin{aligned}
\frac{1}{n_c}\sum_{i=1}^{n_c}\left\{\sum_{j=1}^{n_s}(W_{ij}-\overline{W})\right\}^2 \quad &\to \quad \mathbb{E}\left[\left\{\sum_{j=1}^{n_s}(W_{ij}-\overline{W})\right\}^2\right]=0\\
&= \quad \mathbb{E}\left\{\sum_{j=1}^{n_s}(W_{ij}-\overline{W})^2+\sum_{j\neq j'}(W_{ij}-\overline{W})(W_{ij'}-\overline{W})\right\}\\
&= \quad \mathbb{E}\left\{\sum_{j=1}^{n_s}(W_{ij}-\overline{W})^2\right\}+\mathbb{E}\left\{\sum_{j\neq j'}(W_{ij}-\overline{W})(W_{ij'}-\overline{W})\right\}
\end{aligned}
$$

$$
\frac{1}{n_c}\sum_{i=1}^{n_c}\left[\sum_{j=1}^{n_s}(W_{ij}-\overline{W})\sum_{j=1}^{n_s}(W_{ij}-\overline{W})\boldsymbol{X}_{ij}\right]\to\mathbb{E}\left[\sum_{j=1}^{n_s}(W_{ij}-\overline{W})\sum_{j=1}^{n_s}(W_{ij}-\overline{W})\boldsymbol{X}_{ij}\right]=\mathbf{0}_{p\times 1}
$$

These help to return the limit

$$
\boldsymbol{Q}=\lim_{n_c\to\infty}\frac{1}{n_c}\boldsymbol{Q}_{(n_c,n_s,m)}=m^2n_s^2\begin{bmatrix}1 & 0 & \boldsymbol{\mu}_1^T & \mathbf{0}_{p\times 1}^T\\ 0 & 0 & \mathbf{0}_{p\times 1}^T & \mathbf{0}_{p\times 1}^T\\ \boldsymbol{\mu}_1 & \mathbf{0}_{p\times 1} & \boldsymbol{H}_2 & \mathbf{0}_{p\times p}\\ \mathbf{0}_{p\times 1} & \mathbf{0}_{p\times 1} & \mathbf{0}_{p\times p} & \mathbf{0}_{p\times p}\end{bmatrix}.
$$

Then, it comes to $\boldsymbol{U}=c\boldsymbol{S}+d\boldsymbol{T}+e\boldsymbol{Q}=\begin{bmatrix}\boldsymbol{A} & \boldsymbol{B}\\ \boldsymbol{C} & \boldsymbol{D}\end{bmatrix}$, where

$$
\begin{aligned}
\boldsymbol{A}&=\begin{bmatrix}n_sm(c+dm+en_sm) & 0\\ 0 & n_sm\sigma_w^2(c+dm)\end{bmatrix},\\
\boldsymbol{B}&=\begin{bmatrix}n_sm(c+dm+en_sm)\boldsymbol{\mu}_1^T & \mathbf{0}_{p\times 1}^T\\ \mathbf{0}_{p\times 1}^T & n_sm\sigma_w^2(c+dm)\boldsymbol{\mu}_1^T\end{bmatrix}=\boldsymbol{A}\otimes\boldsymbol{\mu}_1^T=\boldsymbol{C}^T,\\
\boldsymbol{D}&=\begin{bmatrix}n_sm(c\boldsymbol{M}_2+dm\boldsymbol{\Upsilon}_2+en_sm\boldsymbol{H}_2) & \mathbf{0}_{p\times p}\\ \mathbf{0}_{p\times p} & n_sm\sigma_w^2(c\boldsymbol{M}_2+dm\boldsymbol{\Upsilon}_2)\end{bmatrix}.
\end{aligned}
$$

Since the lower-right block of $\sigma_{y|x}^2\boldsymbol{U}^{-1}$ is $\sigma_{y|x}^2(\boldsymbol{D}-\boldsymbol{C}\boldsymbol{A}^{-1}\boldsymbol{B})^{-1}$, its lower-right element becomes

$$
\begin{aligned}
\lim_{n_c\to\infty}n_c\mathrm{cov}(\widehat{\boldsymbol{b}}_4)=\lim_{n_c\to\infty}n_c\mathrm{cov}(\widehat{\boldsymbol{\beta}}_4)&=\frac{\sigma_{y|x}^2}{n_sm\sigma_w^2}\left\{\frac{1}{\lambda_1}(\boldsymbol{M}_2-\boldsymbol{\mu}_1\boldsymbol{\mu}_1^T)-\frac{\lambda_2-\lambda_1}{\lambda_1\lambda_2}(\boldsymbol{\Upsilon}_2-\boldsymbol{\mu}_1\boldsymbol{\mu}_1^T)\right\}^{-1}\\
&=\frac{\sigma_{y|x}^2}{n_sm\sigma_w^2}\boldsymbol{\Omega}_x^{-1/2}\left\{\kappa_{2,(2)}\boldsymbol{\Upsilon}_x+\kappa_{0,(2)}\boldsymbol{\Gamma}_0\right\}^{-1}\boldsymbol{\Omega}_x^{-1/2},
\end{aligned}
$$

where

$$
\kappa_{2,(2)}=\frac{1}{\lambda_1}-\frac{\lambda_2-\lambda_1}{m\lambda_1\lambda_2},\quad \kappa_{0,(2)}=-\frac{\lambda_2-\lambda_1}{\lambda_1\lambda_2}\times\frac{m-1}{m}.
$$

And we can finally obtain

$$\boldsymbol{\Omega}_{4,(2)} = \lim_{n_c \to \infty} n_c n_s m \text{cov}(\widehat{\boldsymbol{\beta}}_4) = \frac{\sigma^2_{y|x}}{\overline{W}(1-\overline{W})} \boldsymbol{\Omega}_x^{-1/2} \left\{ \kappa_{2,(2)} \boldsymbol{\Upsilon}_x + \kappa_{0,(2)} \boldsymbol{\Gamma}_0 \right\}^{-1} \boldsymbol{\Omega}_x^{-1/2}.$$

C.4 Part (c)

Finally, we consider the multivariate case when randomization is carried out at the participant level. We proceed in the same strategies and define the collection of design points as $\boldsymbol{Z}_{ijk} = (1, (W_{ijk} - \overline{W}), \boldsymbol{X}_{ijk}^T, (W_{ijk} - \overline{W})\boldsymbol{X}_{ijk}^T)^T$. With the same target, we derive each necessary matrix components $\boldsymbol{S}_{(n_c,n_s,m)}$, $\boldsymbol{T}_{(n_c,n_s,m)}$, $\boldsymbol{Q}_{(n_c,n_s,m)}$, and their corresponding limits. First,

$$\begin{aligned}
\boldsymbol{S}_{(n_c,n_s,m)} &= \sum_{i=1}^{n_c} \sum_{j=1}^{n_s} \sum_{k=1}^{m} \boldsymbol{Z}_{ijk} \boldsymbol{Z}_{ijk}^T \\
&= \sum_{i=1}^{n_c} \sum_{j=1}^{n_s} \sum_{k=1}^{m} \begin{bmatrix} 1 & W_{ijk} - \overline{W} & \boldsymbol{X}_{ijk}^T & (W_{ijk} - \overline{W})\boldsymbol{X}_{ijk}^T \\ W_{ijk} - \overline{W} & (W_{ijk} - \overline{W})^2 & (W_{ijk} - \overline{W})\boldsymbol{X}_{ijk}^T & (W_{ijk} - \overline{W})^2\boldsymbol{X}_{ijk}^T \\ X_{ijk} & (W_{ijk} - \overline{W})\boldsymbol{X}_{ijk} & \boldsymbol{X}_{ijk}\boldsymbol{X}_{ijk}^T & (W_{ijk} - \overline{W})\boldsymbol{X}_{ijk}\boldsymbol{X}_{ijk}^T \\ (W_{ijk} - \overline{W})\boldsymbol{X}_{ijk} & (W_{ijk} - \overline{W})^2\boldsymbol{X}_{ijk} & (W_{ijk} - \overline{W})\boldsymbol{X}_{ijk}\boldsymbol{X}_{ijk}^T & (W_{ijk} - \overline{W})^2\boldsymbol{X}_{ijk}\boldsymbol{X}_{ijk}^T \end{bmatrix}.
\end{aligned}$$

Note that the limit matrix $\boldsymbol{S}$ will be totally the same under the three randomization scenarios. Then,

$$\begin{aligned}
\boldsymbol{T}_{(n_c,n_s,m)} &= \sum_{i=1}^{n_c} \sum_{j=1}^{n_s} \left(\sum_{k=1}^{m} \boldsymbol{Z}_{ijk} \right) \left(\sum_{k=1}^{m} \boldsymbol{Z}_{ijk}^T \right) \\
&= \sum_{i=1}^{n_c} \sum_{j=1}^{n_s} \left[\begin{matrix} m^2 & m\sum_{k=1}^m (W_{ijk} - \overline{W}) & \cdots \\ m\sum_{k=1}^m (W_{ijk} - \overline{W}) & \{\sum_{k=1}^m (W_{ijk} - \overline{W})\}^2 & \cdots \\ m^2 \overline{\boldsymbol{X}}_{ij} & m\overline{\boldsymbol{X}}_{ij} \sum_{k=1}^m (W_{ijk} - \overline{W}) & \cdots \\ m\sum_{k=1}^m (W_{ijk} - \overline{W})\boldsymbol{X}_{ijk} & \sum_{k=1}^m (W_{ijk} - \overline{W}) \sum_{k=1}^m (W_{ijk} - \overline{W})\boldsymbol{X}_{ijk} & \cdots \end{matrix} \right. \\
&\qquad \left. \begin{matrix} \cdots & m^2 \overline{\boldsymbol{X}}_{ij}^T & m\sum_{k=1}^m (W_{ijk} - \overline{W})\overline{\boldsymbol{X}}_{ij}^T \\ \cdots & m\overline{\boldsymbol{X}}_{ij}^T \sum_{k=1}^m (W_{ijk} - \overline{W}) & \sum_{k=1}^m (W_{ijk} - \overline{W}) \sum_{k=1}^m (W_{ijk} - \overline{W})\boldsymbol{X}_{ijk}^T \\ \cdots & m^2 \overline{\boldsymbol{X}}_{ij} \overline{\boldsymbol{X}}_{ij}^T & m\overline{\boldsymbol{X}}_{ij} \sum_{k=1}^m (W_{ijk} - \overline{W})\boldsymbol{X}_{ijk}^T \\ \cdots & m\overline{\boldsymbol{X}}_{ij} \sum_{k=1}^m (W_{ijk} - \overline{W})\boldsymbol{X}_{ijk}^T & \sum_{k=1}^m (W_{ijk} - \overline{W})\boldsymbol{X}_{ijk} \sum_{k=1}^m (W_{ijk} - \overline{W})\boldsymbol{X}_{ijk}^T \end{matrix} \right].
\end{aligned}$$

Because of the participant-level randomization is conducted within each subcluster, we have $\sum_{k=1}^m (W_{ijk} - \overline{W}) = 0$. This will lead to many zeros in the matrix above, similar to the derivation in the subcluster-level randomization scenario. We therefore have

$$\boldsymbol{T} = \lim_{n_c \to \infty} \frac{1}{n_c} \boldsymbol{T}_{(n_c,n_s,m)} = \begin{bmatrix} n_s m^2 & 0 & n_s m^2 \boldsymbol{\mu}_1^T & \mathbf{0}_{p\times 1}^T \\ 0 & 0 & \mathbf{0}_{p\times 1}^T & \mathbf{0}_{p\times 1}^T \\ n_s m^2 \boldsymbol{\mu}_1 & \mathbf{0}_{p\times 1} & n_s m^2 \boldsymbol{\Upsilon}_2 & \mathbf{0}_{p\times p} \\ \mathbf{0}_{p\times 1} & \mathbf{0}_{p\times 1}^T & \mathbf{0}_{p\times p} & \mathbf{0}_{p\times p} \end{bmatrix},$$

where $\boldsymbol{\mu}_1$ and $\boldsymbol{\Upsilon}_2$ were defined previously. Next, we derive

$$\boldsymbol{Q}_{(n_c,n_s,m)} = \sum_{i=1}^{n_c} \left(\sum_{j=1}^{n_s} \sum_{k=1}^{m} \boldsymbol{Z}_{ijk} \right) \left(\sum_{j=1}^{n_s} \sum_{k=1}^{m} \boldsymbol{Z}_{ijk}^T \right)$$

$$= \sum_{i=1}^{n_c} \left[\begin{matrix} m^2 n_s^2 & m n_s \sum_{j=1}^{n_s} \sum_{k=1}^{m} (W_{ijk} - \overline{W}) & \cdots \\ m n_s \sum_{j=1}^{n_s} \sum_{k=1}^{m} (W_{ijk} - \overline{W}) & \{\sum_{j=1}^{n_s} \sum_{k=1}^{m} (W_{ijk} - \overline{W})\}^2 & \cdots \\ m^2 n_s^2 \overline{\boldsymbol{X}}_i & m n_s \overline{\boldsymbol{X}}_i \sum_{j=1}^{n_s} \sum_{k=1}^{m} (W_{ijk} - \overline{W}) & \cdots \\ m n_s \sum_{j=1}^{n_s} \sum_{k=1}^{m} (W_{ijk} - \overline{W}) \boldsymbol{X}_{ijk} & \sum_{j=1}^{n_s} \sum_{k=1}^{m} (W_{ijk} - \overline{W}) \sum_{j=1}^{n_s} \sum_{k=1}^{m} (W_{ijk} - \overline{W}) \boldsymbol{X}_{ijk} & \cdots \end{matrix} \right.$$

$$\left. \begin{matrix} \cdots & m^2 n_s^2 \overline{\boldsymbol{X}}_i^T & m n_s \sum_{j=1}^{n_s} \sum_{k=1}^{m} (W_{ijk} - \overline{W}) \boldsymbol{X}_{ijk}^T \\ \cdots & m n_s \overline{\boldsymbol{X}}_i^T \sum_{j=1}^{n_s} \sum_{k=1}^{m} (W_{ijk} - \overline{W}) & \sum_{j=1}^{n_s} \sum_{k=1}^{m} (W_{ijk} - \overline{W}) \sum_{j=1}^{n_s} \sum_{k=1}^{m} (W_{ijk} - \overline{W}) \boldsymbol{X}_{ijk}^T \\ \cdots & m^2 n_s^2 \overline{\boldsymbol{X}}_i \overline{\boldsymbol{X}}_i^T & m n_s \overline{\boldsymbol{X}}_i \sum_{j=1}^{n_s} \sum_{k=1}^{m} (W_{ijk} - \overline{W}) \boldsymbol{X}_{ijk}^T \\ \cdots & m n_s \overline{\boldsymbol{X}}_i \sum_{j=1}^{n_s} \sum_{k=1}^{m} (W_{ijk} - \overline{W}) \boldsymbol{X}_{ijk}^T & \sum_{j=1}^{n_s} \sum_{k=1}^{m} (W_{ijk} - \overline{W}) \boldsymbol{X}_{ijk} \sum_{j=1}^{n_s} \sum_{k=1}^{m} (W_{ijk} - \overline{W}) \boldsymbol{X}_{ijk}^T \end{matrix} \right],$$

which suggests the limit

$$\boldsymbol{Q} = \lim_{n_c \to \infty} \frac{1}{n_c} \boldsymbol{Q}_{(n_c,n_s,m)} = \begin{bmatrix} m^2 n_s^2 & 0 & m^2 n_s^2 \boldsymbol{\mu}_1^T & \boldsymbol{0}_{p\times 1}^T \\ 0 & 0 & \boldsymbol{0}_{p\times 1}^T & \boldsymbol{0}_{p\times 1}^T \\ m^2 n_s^2 \boldsymbol{\mu}_1 & \boldsymbol{0}_{p\times 1} & m^2 n_s^2 \boldsymbol{H}_2 & \boldsymbol{0}_{p\times p} \\ \boldsymbol{0}_{p\times 1} & \boldsymbol{0}_{p\times 1} & \boldsymbol{0}_{p\times p} & \boldsymbol{0}_{p\times p} \end{bmatrix}.$$

Then, similarly to the fist two scenarios, we have $\boldsymbol{U} = c\boldsymbol{S} + d\boldsymbol{T} + e\boldsymbol{Q} = \begin{bmatrix} \boldsymbol{A} & \boldsymbol{B} \\ \boldsymbol{C} & \boldsymbol{D} \end{bmatrix}$, where

$$\begin{aligned} \boldsymbol{A} &= \begin{bmatrix} n_s m(c + dm + e n_s m) & 0 \\ 0 & n_s m \sigma_w^2 c \end{bmatrix}, \\ \boldsymbol{B} &= \begin{bmatrix} n_s m(c + dm + e n_s m)\boldsymbol{\mu}_1 & \boldsymbol{0}_{p\times 1}^T \\ \boldsymbol{0}_{p\times 1}^T & n_s m \sigma_w^2 c \boldsymbol{\mu}_1^T \end{bmatrix} = \boldsymbol{A} \otimes \boldsymbol{\mu}_1^T = \boldsymbol{C}^T, \\ \boldsymbol{D} &= \begin{bmatrix} n_s m(c\boldsymbol{M}_2 + dm\boldsymbol{\Upsilon}_2 + e n_s m \boldsymbol{H}_2) & \boldsymbol{0}_{p\times p} \\ \boldsymbol{0}_{p\times p} & n_s m \sigma_w^2 c \boldsymbol{M}_2 \end{bmatrix}. \end{aligned}$$

By the same block matrix inversion formula, we can get

$$\lim_{n_c \to \infty} n_c \text{cov}(\widehat{\boldsymbol{b}}_4) = \lim_{n_c \to \infty} n_c \text{cov}(\widehat{\boldsymbol{\beta}}_4) = \frac{\sigma_{y|x}^2}{n_s m \sigma_w^2} \left\{ c(\boldsymbol{M}_2 - \boldsymbol{\mu}_1 \boldsymbol{\mu}_1^T) \right\}^{-1} = \frac{\sigma_{y|x}^2 \lambda_1}{n_s m \sigma_w^2} \boldsymbol{\Omega}_x^{-1/2} \boldsymbol{\Upsilon}_x^{-1} \boldsymbol{\Omega}_x^{-1/2}.$$

Finally, we summarize that when randomization is carried out at the participant level,

$$\boldsymbol{\Omega}_{4,(1)} = \lim_{n_c \to \infty} n_c n_s m \text{cov}(\widehat{\boldsymbol{\beta}}_4) = \frac{\sigma_{y|x}^2 \lambda_1}{\overline{W}(1 - \overline{W})} \times \boldsymbol{\Omega}_x^{-1/2} \boldsymbol{\Upsilon}_x^{-1} \boldsymbol{\Omega}_x^{-1/2}.$$

C.5 Ordering statements

We now show that the covariance matrices have a strict ordering such that $\boldsymbol{\Omega}_{4,(3)} - \boldsymbol{\Omega}_{4,(2)}$ and $\boldsymbol{\Omega}_{4,(2)} - \boldsymbol{\Omega}_{4,(1)}$ are both non-negative definite (or equivalently, the covariance matrices have a Löwner ordering such that $\boldsymbol{\Omega}_{4,(3)} \succeq \boldsymbol{\Omega}_{4,(2)} \succeq \boldsymbol{\Omega}_{4,(1)}$), and equal to zero when $\lambda_1 = \lambda_2 = \lambda_3 = 1$. We focus

on the first statement, and the second statement will follow accordingly.

To show that $\mathbf{\Omega}_{4,(3)} - \mathbf{\Omega}_{4,(2)}$ is non-negative definite, it is equivalent to show that $\mathbf{\Omega}_{4,(2)}^{-1} - \mathbf{\Omega}_{4,(3)}^{-1}$ is non-negative definite. From previous derivations, we have

$$\begin{aligned}\mathbf{\Omega}_{4,(2)}^{-1} - \mathbf{\Omega}_{4,(3)}^{-1} &= \frac{\sigma_w^2}{\sigma_{y|x}^2}\mathbf{\Omega}_x^{1/2}\left\{\kappa_{2,(2)}\mathbf{\Upsilon}_x + \kappa_{0,(2)}\mathbf{\Gamma}_0\right\}\mathbf{\Omega}_x^{1/2} \\ &\quad - \frac{\sigma_w^2}{\sigma_{y|x}^2}\mathbf{\Omega}_x^{1/2}\left\{\kappa_{2,(3)}\mathbf{\Upsilon}_x + \kappa_{1,(3)}\mathbf{\Gamma}_1 + \kappa_{0,(3)}\mathbf{\Gamma}_0\right\}\mathbf{\Omega}_x^{1/2} \\ &= \frac{\sigma_w^2}{\sigma_{y|x}^2} \times \frac{\lambda_3 - \lambda_2}{\lambda_2\lambda_3} \times \mathbf{\Omega}_x^{1/2}\left\{\frac{1}{n_s m}\mathbf{\Upsilon}_x + \frac{n_s - 1}{n_s}\mathbf{\Gamma}_1 + \frac{m-1}{n_s m}\mathbf{\Gamma}_0\right\}\mathbf{\Omega}_x^{1/2}.\end{aligned}$$

Recall that $\lambda_1 = 1 - \alpha_0$, $\lambda_2 = \lambda_1 + m(\alpha_0 - \alpha_1)$, and $\lambda_3 = \lambda_2 + n_s m \alpha_1$. Since $1 > \alpha_0 \geq \alpha_1 \geq 0$, it follows $\lambda_3 \geq \lambda_2 \geq \lambda_1 > 0$, which further implies that $\mathbf{\Omega}_{4,(2)}^{-1} - \mathbf{\Omega}_{4,(3)}^{-1}$ is non-negative definite. Similarly, we have

$$\begin{aligned}\mathbf{\Omega}_{4,(1)}^{-1} - \mathbf{\Omega}_{4,(2)}^{-1} &= \frac{\sigma_w^2}{\sigma_{y|x}^2}\mathbf{\Omega}_x^{1/2}\left\{\lambda_1^{-1}\mathbf{\Upsilon}_x\right\}\mathbf{\Omega}_x^{1/2} - \frac{\sigma_w^2}{\sigma_{y|x}^2}\mathbf{\Omega}_x^{1/2}\left\{\kappa_{2,(2)}\mathbf{\Upsilon}_x + \kappa_{0,(2)}\mathbf{\Gamma}_0\right\}\mathbf{\Omega}_x^{1/2} \\ &= \frac{\sigma_w^2}{\sigma_{y|x}^2} \times \frac{\lambda_2 - \lambda_1}{\lambda_1\lambda_2} \times \mathbf{\Omega}_x^{1/2}\left\{\frac{1}{m}\mathbf{\Upsilon}_x + \frac{m-1}{m}\mathbf{\Gamma}_0\right\}\mathbf{\Omega}_x^{1/2},\end{aligned}$$

which is non-negative definite following the same arguments. $\mathbf{\Omega}_{4,(3)} - \mathbf{\Omega}_{4,(2)}$ and $\mathbf{\Omega}_{4,(2)} - \mathbf{\Omega}_{4,(1)}$ are equal to zero when $\lambda_1 = \lambda_2 = \lambda_3 = 1$, which means there is no residual clustering in a three-level design.

C.6 Covariance expressions when the vector of effect modifiers is measured at the subcluster or cluster level

As in Section 2.1 of the main manuscript, we can further simplify the covariance expressions when the vector of effect modifiers is measured at the subcluster or cluster level. Specifically, when the effect modifier is measured at the subcluster level, then $\mathbf{\Gamma}_0 = \mathbf{\Upsilon}_x$, and we obtain

$$\begin{aligned}\mathbf{\Omega}_{4,(3)} &= \frac{\sigma_{y|x}^2}{\overline{W}(1-\overline{W})} \times \mathbf{\Omega}_x^{-1/2}\left\{\kappa_{2,(3)}^*\mathbf{\Upsilon}_x + \kappa_{1,(3)}^*\mathbf{\Gamma}_1\right\}^{-1}\mathbf{\Omega}_x^{-1/2}, \\ \mathbf{\Omega}_{4,(2)} &= \frac{\sigma_{y|x}^2}{\overline{W}(1-\overline{W})} \times \lambda_2\mathbf{\Omega}_x^{-1/2}\mathbf{\Upsilon}_x^{-1}\mathbf{\Omega}_x^{-1/2},\end{aligned} \tag{7}$$

where the linear coefficients are given by

$$\kappa_{2,(3)}^* = \frac{1}{\lambda_2} - \frac{\lambda_3 - \lambda_2}{n_s\lambda_2\lambda_3}, \quad \kappa_{1,(3)}^* = -\frac{(n_s - 1)(\lambda_3 - \lambda_2)}{n_s\lambda_2\lambda_3}.$$

When the vector of effect modifiers is measured at the cluster level, we have $\mathbf{\Gamma}_0 = \mathbf{\Gamma}_1 = \mathbf{\Upsilon}_x$, and

$$\mathbf{\Omega}_{4,(3)} = \frac{\sigma_{y|x}^2}{\overline{W}(1-\overline{W})} \times \lambda_3\mathbf{\Omega}_x^{-1/2}\mathbf{\Upsilon}_x^{-1}\mathbf{\Omega}_x^{-1/2},$$

but $\mathbf{\Omega}_{4,(2)}$ remains identical to (7). In both cases, $\mathbf{\Omega}_{4,(1)}$ remains the same as in Theorem 2.2 of the main manuscript. Finally, if there is no residual clustering in a three-level design such that

$\alpha_0 = \alpha_1 = 0$, then

$$\mathbf{\Omega}_{4,(3)} = \mathbf{\Omega}_{4,(2)} = \mathbf{\Omega}_{4,(1)} = \frac{\sigma^2_{y|x}}{\overline{W}(1-\overline{W})} \times \mathbf{\Omega}_x^{-1/2}\mathbf{\Upsilon}_x^{-1}\mathbf{\Omega}_x^{-1/2},$$

which equivalent to the covariance matrix of the interaction effect parameter estimator in a simple ANCOVA model without any residual clustering (Yang et al., 2020). Under the further assumption that the p effect modifiers are mutually independent, we have $\mathbf{\Upsilon}_x = \boldsymbol{I}_p$ and

$$\mathbf{\Omega}_{4,(3)} = \mathbf{\Omega}_{4,(2)} = \mathbf{\Omega}_{4,(1)} = \frac{\sigma^2_{y|x}}{\overline{W}(1-\overline{W})}\mathbf{\Omega}_x^{-1},$$

in which case the variances $\widetilde{\sigma}^2_{4,(3)}$, $\widetilde{\sigma}^2_{4,(2)}$, $\widetilde{\sigma}^2_{4,(1)}$ with a univariate effect modifier (defined in Section 2.1 of the main manuscript) are directly applicable to each of the p effect modifiers.

Web Appendix D Variance expression for HTE analysis with multivariate effect modifiers allowing for between- and within-cluster effects

D.1 Participant-level effect modifier

The results in Theorem 2 can be applied to address the HTE analysis with a univariate effect modifier but allowing for differential effects of the covariate aggregated at each clustering level (or sometimes called the contextual effects in CRTs; also see Raudenbush (1997) and Begg and Parides (2003)). We assume the effect modifier is global mean centered without loss of generality ($\mu_1 = 0$), and provide a derivation of such results to support the generic discussion in Section 2.3 of the main text. Recall that the contextual HTE model is considered as

$$\begin{aligned} Y_{ijk} &= \beta_1 + \beta_2 W_{ijk} + \beta_{31}(X_{ijk} - \overline{X}_{ij}) + \beta_{32}(\overline{X}_{ij} - \overline{X}_i) + \beta_{33}\overline{X}_i + \beta_{41}W_{ijk}(X_{ijk} - \overline{X}_{ij}) \\ &\quad + \beta_{42}W_{ijk}(\overline{X}_{ij} - \overline{X}_i) + \beta_{43}W_{ijk}\overline{X}_i + \gamma_i + u_{ij} + \epsilon_{ijk}. \end{aligned}$$

in which case the null hypothesis of no HTE is given by $H_0 : \beta_{41} = \beta_{42} = \beta_{43} = 0$. When it is assumed that $\beta_{31} = \beta_{32} = \beta_{33}$ and $\beta_{41} = \beta_{42} = \beta_{43}$, this model reduces to the univariate HTE model assuming homogeneous covariate effects at each clustering level.

Here we compute $\mathbf{\Gamma}_0$, $\mathbf{\Gamma}_1$, $\mathbf{\Upsilon}_x$, and $\mathbf{\Omega}_x$ with $\boldsymbol{X}_{ijk} = (X_{ijk} - \overline{X}_{ij}, \overline{X}_{ij} - \overline{X}_i, \overline{X}_i)^T$ and $\mathbb{E}(X_{ijk}) = 0$. We thus have $\mathbb{E}(\boldsymbol{X}_{ijk}) = \boldsymbol{\mu}_1 = \mathbf{0}_{3\times 1}$. Recall,

$$\begin{aligned} \mathbf{\Gamma}_0 &= \mathbf{\Omega}_x^{-1/2}\mathbb{E}(\boldsymbol{X}_{ijk}\boldsymbol{X}_{ijk'}^T)\mathbf{\Omega}_x^{-1/2}, \ \forall k \neq k', \\ \mathbf{\Gamma}_1 &= \mathbf{\Omega}_x^{-1/2}\mathbb{E}(\boldsymbol{X}_{ijk}\boldsymbol{X}_{ij'k'}^T)\mathbf{\Omega}_x^{-1/2}, \ \forall j \neq j', \ k \neq k', \\ \mathbf{\Upsilon}_x &= \mathbf{\Omega}_x^{-1/2}\mathbb{E}(\boldsymbol{X}_{ijk}\boldsymbol{X}_{ijk}^T)\mathbf{\Omega}_x^{-1/2} = \mathbf{\Omega}_x^{-1/2}\boldsymbol{M}_2\mathbf{\Omega}_x^{-1/2}, \end{aligned}$$

where $\mathbf{\Omega}_x = \text{diag}(\boldsymbol{M}_2)$, with $\boldsymbol{M}_2 = \lim_{n_c\to\infty}(n_c n_s m)^{-1}\sum_{i=1}^{n_c}\sum_{j=1}^{n_s}\sum_{k=1}^{m}\boldsymbol{X}_{ijk}\boldsymbol{X}_{ijk}^T$. We then

establish the following

$$\mathbb{E}(X_{ijk}^2) = \sigma_x^2, \quad \mathbb{E}(\overline{X}_{ij}^2) = \frac{\sigma_x^2}{m}\{1 + (m-1)\rho_0\}, \quad \mathbb{E}(\overline{X}_i^2) = \frac{\sigma_x^2}{n_s m}\{1 + (m-1)\rho_0 + (n_s - 1)m\rho_1\},$$
$$\mathbb{E}(\overline{X}_{ij}X_{ijk}) = \frac{\sigma_x^2}{m}\{1 + (m-1)\rho_0\}, \quad \mathbb{E}(\overline{X}_i X_{ijk}) = \frac{\sigma_x^2}{n_s m}\left\{1 + (m-1)\rho_0 + (n_s - 1)m\rho_1\right\},$$
$$\mathbb{E}(\overline{X}_i \overline{X}_{ij}) = \frac{\sigma_x^2}{n_s m}\{1 + (m-1)\rho_0 + (n_s - 1)m\rho_1\}.$$

We first compute $\boldsymbol{\Omega}_x = \text{diag}(\boldsymbol{M}_2)$, where

$$\begin{aligned}
\boldsymbol{M}_2 &= \mathbb{E}(\boldsymbol{X}_{ijk}\boldsymbol{X}_{ijk}^T) \\
&= \mathbb{E}\begin{bmatrix} (X_{ijk} - \overline{X}_{ij})^2 & (X_{ijk} - \overline{X}_{ij})(\overline{X}_{ij} - \overline{X}_i) & (X_{ijk} - \overline{X}_{ij})\overline{X}_i \\ (X_{ijk} - \overline{X}_{ij})(\overline{X}_{ij} - \overline{X}_i) & (\overline{X}_{ij} - \overline{X}_i)^2 & (\overline{X}_{ij} - \overline{X}_i)\overline{X}_i \\ (X_{ijk} - \overline{X}_{ij})\overline{X}_i & (\overline{X}_{ij} - \overline{X}_i)\overline{X}_i & \overline{X}_i^2 \end{bmatrix} \\
&= \frac{\sigma_x^2}{n_s m}\begin{bmatrix} n_s(m-1)\zeta_1 & (n_s-1)\zeta_2 & 0 \\ (n_s-1)\zeta_2 & (n_s-1)\zeta_2 & 0 \\ 0 & 0 & \zeta_3 \end{bmatrix},
\end{aligned}$$

and

$$\boldsymbol{\Omega}_x = \text{diag}(\boldsymbol{M}_2) = \frac{\sigma_x^2}{n_s m}\begin{bmatrix} n_s(m-1)\zeta_1 & 0 & 0 \\ 0 & (n_s-1)\zeta_2 & 0 \\ 0 & 0 & \zeta_3 \end{bmatrix}.$$

We then compute

$$\boldsymbol{\Upsilon}_x = \boldsymbol{\Omega}_x^{-1/2}\boldsymbol{M}_2\boldsymbol{\Omega}_x^{-1/2} = \begin{bmatrix} 1 & \sqrt{\dfrac{(n_s-1)\zeta_2}{n_s(m-1)\zeta_1}} & 0 \\ \sqrt{\dfrac{(n_s-1)\zeta_2}{n_s(m-1)\zeta_1}} & 1 & 0 \\ 0 & 0 & 1 \end{bmatrix}.$$

We proceed to compute $\boldsymbol{\Gamma}_0$, where

$$\begin{aligned}
\mathbb{E}(\boldsymbol{X}_{ijk}\boldsymbol{X}_{ijk'}^T) &= \mathbb{E}\begin{bmatrix} (X_{ijk} - \overline{X}_{ij})(X_{ijk'} - \overline{X}_{ij}) & (X_{ijk} - \overline{X}_{ij})(\overline{X}_{ij} - \overline{X}_i) & (X_{ijk} - \overline{X}_{ij})\overline{X}_i \\ (X_{ijk'} - \overline{X}_{ij})(\overline{X}_{ij} - \overline{X}_i) & (\overline{X}_{ij} - \overline{X}_i)^2 & (\overline{X}_{ij} - \overline{X}_i)\overline{X}_i \\ (X_{ijk'} - \overline{X}_{ij})\overline{X}_i & (\overline{X}_{ij} - \overline{X}_i)\overline{X}_i & \overline{X}_i^2 \end{bmatrix} \\
&= \frac{\sigma_x^2}{n_s m}\begin{bmatrix} -n_s\zeta_1 & (n_s-1)\zeta_2 & 0 \\ (n_s-1)\zeta_2 & (n_s-1)\zeta_2 & 0 \\ 0 & 0 & \zeta_3 \end{bmatrix},
\end{aligned}$$

then

$$\mathbf{\Gamma}_0 = \mathbf{\Omega}_x^{-1/2}\mathbb{E}(\boldsymbol{X}_{ijk}\boldsymbol{X}_{ijk'}^T)\mathbf{\Omega}_x^{-1/2} = \begin{bmatrix} -\frac{1}{m-1} & \sqrt{\frac{(n_s-1)\zeta_2}{n_s(m-1)\zeta_1}} & 0 \\ \sqrt{\frac{(n_s-1)\zeta_2}{n_s(m-1)\zeta_1}} & 1 & 0 \\ 0 & 0 & 1 \end{bmatrix}.$$

Lastly, we compute $\mathbf{\Gamma}_1$, where

$$\begin{aligned} \mathbb{E}(\boldsymbol{X}_{ijk}\boldsymbol{X}_{ij'k'}^T) &= \mathbb{E}\begin{bmatrix} (X_{ijk}-\overline{X}_{ij})(X_{ij'k'}-\overline{X}_{ij'}) & (X_{ijk}-\overline{X}_{ij})(\overline{X}_{ij'}-\overline{X}_i) & (X_{ijk}-\overline{X}_{ij})\overline{X}_i \\ (X_{ij'k'}-\overline{X}_{ij'})(\overline{X}_{ij}-\overline{X}_i) & (\overline{X}_{ij}-\overline{X}_i)(\overline{X}_{ij'}-\overline{X}_i) & (\overline{X}_{ij}-\overline{X}_i)\overline{X}_i \\ (X_{ij'k'}-\overline{X}_{ij'})\overline{X}_i & (\overline{X}_{ij'}-\overline{X}_i)\overline{X}_i & \overline{X}_i^2 \end{bmatrix} \\ &= \frac{\sigma_x^2}{n_s m}\begin{bmatrix} 0 & 0 & 0 \\ 0 & -\zeta_2 & 0 \\ 0 & 0 & \zeta_3 \end{bmatrix}. \end{aligned}$$

Then

$$\mathbf{\Gamma}_1 = \mathbf{\Omega}_x^{-1/2}\mathbb{E}(\boldsymbol{X}_{ijk}\boldsymbol{X}_{ij'k'}^T)\mathbf{\Omega}_x^{-1/2} = \begin{bmatrix} 0 & 0 & 0 \\ 0 & -\frac{1}{n_s-1} & 0 \\ 0 & 0 & 1 \end{bmatrix}.$$

Based on the above derivations, it is worth noting that the key design expressions for such a decomposed multivariate effect modifier are relatively simple. In fact, if the number of subclusters n_s and the subcluster size m are both large, then we can further obtain the following approximate expressions for sample size determination

$$\mathbf{\Omega}_x \approx \sigma_x^2 \begin{bmatrix} 1-\rho_0 & 0 & 0 \\ 0 & \rho_0-\rho_1 & 0 \\ 0 & 0 & \rho_1 \end{bmatrix},$$

$$\mathbf{\Upsilon}_x \approx \begin{bmatrix} 1 & \sqrt{\frac{\rho_0-\rho_1}{1-\rho_0}} & 0 \\ \sqrt{\frac{\rho_0-\rho_1}{1-\rho_0}} & 1 & 0 \\ 0 & 0 & 1 \end{bmatrix}, \quad \mathbf{\Gamma}_0 \approx \begin{bmatrix} 0 & \sqrt{\frac{\rho_0-\rho_1}{1-\rho_0}} & 0 \\ \sqrt{\frac{\rho_0-\rho_1}{1-\rho_0}} & 1 & 0 \\ 0 & 0 & 1 \end{bmatrix}, \quad \mathbf{\Gamma}_1 \approx \begin{bmatrix} 0 & 0 & 0 \\ 0 & 0 & 0 \\ 0 & 0 & 1 \end{bmatrix}.$$

D.2 Subcluster-level effect modifier

If the effect modifier is measured at the subcluster level and denoted by X_{ij}, recall that the contextual HTE model is given by

$$Y_{ijk} = \beta_1 + \beta_2 W_{ijk} + \beta_{31}(X_{ij}-\overline{X}_i) + \beta_{32}\overline{X}_i + \beta_{41}W_{ijk}(X_{ij}-\overline{X}_i) + \beta_{42}W_{ijk}\overline{X}_i + \gamma_i + u_{ij} + \epsilon_{ijk}.$$

in which case the null hypothesis of no HTE is given by $H_0 : \beta_{41} = \beta_{42} = 0$. We derive explicit expression for the key matrices, $\boldsymbol{\Gamma}_0$, $\boldsymbol{\Gamma}_1$, $\boldsymbol{\Upsilon}_x$, and $\boldsymbol{\Omega}_x$ with such a subcluster-level effect modifier (where $\rho_0 = 1$ by definition) based on the above model. Here we have $\boldsymbol{X}_{ij} = (X_{ij} - \overline{X}_i, \overline{X}_i)^T$ and assume $\mathbb{E}(X_{ij}) = 0$ without loss of generality (otherwise one can always mean center X_{ij}). We thus have $\mathbb{E}(\boldsymbol{X}_{ij}) = \boldsymbol{\mu}_1 = \mathbf{0}_{2\times 1}$. Redefine,

$$\begin{aligned}\boldsymbol{\Gamma}_0 &= \boldsymbol{\Upsilon}_x = \boldsymbol{\Omega}_x^{-1/2}\mathbb{E}(\boldsymbol{X}_{ij}\boldsymbol{X}_{ij}^T)\boldsymbol{\Omega}_x^{-1/2},\\ \boldsymbol{\Gamma}_1 &= \boldsymbol{\Omega}_x^{-1/2}\mathbb{E}(\boldsymbol{X}_{ij}\boldsymbol{X}_{ij'}^T)\boldsymbol{\Omega}_x^{-1/2},\ \forall j \neq j',\end{aligned}$$

where $\boldsymbol{\Omega}_x = \text{diag}(\boldsymbol{M}_2)$, with $\boldsymbol{M}_2 = \lim_{n_c\to\infty}(n_c n_s)^{-1}\sum_{i=1}^{n_c}\sum_{j=1}^{n_s}\boldsymbol{X}_{ij}\boldsymbol{X}_{ij}^T$. We then establish the following

$$\begin{aligned}\mathbb{E}(X_{ij}^2) &= \sigma_x^2,\\ \mathbb{E}(\overline{X}_i^2) &= \frac{\sigma_x^2}{n_s}\{1 + (n_s - 1)\rho_1\},\\ \mathbb{E}(\overline{X}_i X_{ij}) &= \frac{\sigma_x^2}{n_s}\{1 + (n_s - 1)\rho_1\}.\end{aligned}$$

We first compute $\boldsymbol{\Omega}_x = \text{diag}(\boldsymbol{M}_2)$, where

$$\boldsymbol{M}_2 = \mathbb{E}(\boldsymbol{X}_{ij}\boldsymbol{X}_{ij}^T) = \mathbb{E}\begin{bmatrix}(X_{ij} - \overline{X}_i)^2 & (X_{ij} - \overline{X}_i)\overline{X}_i\\ (X_{ij} - \overline{X}_i)\overline{X}_i & \overline{X}_i^2\end{bmatrix} = \frac{\sigma_x^2}{n_s}\begin{bmatrix}(n_s - 1)(1 - \rho_1) & 0\\ 0 & 1 + (n_s - 1)\rho_1\end{bmatrix},$$

and therefore

$$\boldsymbol{\Omega}_x = \text{diag}(\boldsymbol{M}_2) = \frac{\sigma_x^2}{n_s}\begin{bmatrix}(n_s - 1)(1 - \rho_1) & 0\\ 0 & 1 + (n_s - 1)\rho_1\end{bmatrix}.$$

We then compute

$$\boldsymbol{\Gamma}_0 = \boldsymbol{\Upsilon}_x = \boldsymbol{\Omega}_x^{-1/2}\boldsymbol{M}_2\boldsymbol{\Omega}_x^{-1/2} = \begin{bmatrix}1 & 0\\ 0 & 1\end{bmatrix}.$$

We proceed to compute $\boldsymbol{\Gamma}_1$, where

$$\mathbb{E}(\boldsymbol{X}_{ij}\boldsymbol{X}_{ij'}^T) = \mathbb{E}\begin{bmatrix}(X_{ij} - \overline{X}_i)(X_{ij'} - \overline{X}_i) & (X_{ij} - \overline{X}_i)\overline{X}_i\\ (X_{ij'} - \overline{X}_i)\overline{X}_i & \overline{X}_i^2\end{bmatrix} = \frac{\sigma_x^2}{n_s}\begin{bmatrix}\rho_1 - 1 & 0\\ 0 & 1 + (n_s - 1)\rho_1\end{bmatrix}.$$

Then

$$\boldsymbol{\Gamma}_1 = \boldsymbol{\Omega}_x^{-1/2}\mathbb{E}(\boldsymbol{X}_{ij}\boldsymbol{X}_{ij'}^T)\boldsymbol{\Omega}_x^{-1/2} = \begin{bmatrix}-\dfrac{1}{n_s - 1} & 0\\ 0 & 1\end{bmatrix}.$$

Finally, when the number of subclusters n_s is large, an approximate sample size procedure can

use the following approximate and yet simpler matrices:

$$\boldsymbol{\Omega}_x \approx \sigma_x^2 \begin{bmatrix} 1-\rho_1 & 0 \\ 0 & \rho_1 \end{bmatrix}, \quad \boldsymbol{\Upsilon}_x = \boldsymbol{\Gamma}_0 \approx \begin{bmatrix} 1 & 0 \\ 0 & 1 \end{bmatrix}, \quad \boldsymbol{\Gamma}_1 \approx \begin{bmatrix} 0 & 0 \\ 0 & 1 \end{bmatrix},$$

in which case we have from Theorem 2(b)

$$\boldsymbol{\Omega}_{4,(2)} \approx \frac{\sigma^2_{y|x}\lambda_2}{\overline{W}(1-\overline{W})}\boldsymbol{\Omega}_x^{-1} = \frac{\sigma^2_{y|x}\lambda_2}{\overline{W}(1-\overline{W})\sigma_x^2} \begin{bmatrix} \frac{1}{1-\rho_1} & 0 \\ 0 & \frac{1}{\rho_1} \end{bmatrix}$$

Web Appendix E Variance expressions for covariate-adjusted average treatment effect estimators (Theorem 2.2)

E.1 Univariate effect modifier

E.1.1 Cluster-level randomization

When considering the randomization at the cluster level, we could use the following model to analyze individual outcomes,

$$Y_{ijk} = \beta_1 + \beta_2 W_i + \beta_3 X_{ijk} + \beta_4 W_i X_{ijk} + \gamma_i + u_{ij} + \epsilon_{ijk}.$$

The upper-left block of $\sigma^2_{y|x}\boldsymbol{U}^{-1}$ is given by $\sigma^2_{y|x}(\boldsymbol{A} - \boldsymbol{B}\boldsymbol{D}^{-1}\boldsymbol{C})^{-1}$, and the lower-right entry of it becomes

$$\lim_{n_c\to\infty} n_c \text{var}(\widehat{b}_2) = \lim_{n_c\to\infty} n_c \text{var}(\widehat{\beta}_2) = \frac{\sigma^2_{y|x}(c\mu_2 + dm\tau_2 + en_s m\eta_2)}{n_s m\sigma_w^2(c + dm + en_s m)\{c(\mu_2 - \mu_1^2) + dm(\tau_2 - \mu_1^2) + en_s m(\eta_2 - \mu_1^2)\}}.$$

The lower-left block of $\sigma^2_{y|x}\boldsymbol{U}^{-1}$ is given by $-\sigma^2_{y|x}\boldsymbol{D}^{-1}\boldsymbol{C}(\boldsymbol{A} - \boldsymbol{B}\boldsymbol{D}^{-1}\boldsymbol{C})^{-1}$, which will be the same as the upper-right block in this case. The lower-right element can then be given as

$$\lim_{n_c\to\infty} n_c \text{cov}(\widehat{b}_2, \widehat{b}_4) = \lim_{n_c\to\infty} n_c \text{cov}(\widehat{\beta}_2, \widehat{\beta}_4) = -\frac{\sigma^2_{y|x}\mu_1}{n_s m\sigma_w^2\{c(\mu_2 - \mu_1^2) + dm(\tau_2 - \mu_1^2) + en_s m(\eta_2 - \mu_1^2)\}}$$
$$= -\lim_{n_c\to\infty} n_c \mu_1 \text{var}(\widehat{\beta}_4)$$

Finally, the limit variance associated with the average treatment effect estimator is given by

$$\begin{aligned}\lim_{n_c\to\infty} n_c \text{var}(\widehat{\beta}_2+\mu_1\widehat{\beta}_4) &= \lim_{n_c\to\infty} n_c \text{var}(\widehat{\beta}_2) + \lim_{n_c\to\infty} n_c\mu_1^2 \text{var}(\widehat{\beta}_4) + 2\lim_{n_c\to\infty} n_c\mu_1 \text{cov}(\widehat{\beta}_2,\widehat{\beta}_4)\\ &= \frac{\sigma^2_{y|x}(c\mu_2+dm\tau_2+en_s m\eta_2)}{n_s m\sigma_w^2(c+dm+en_s m)\{c(\mu_2-\mu_1^2)+dm(\tau_2-\mu_1^2)+en_s m(\eta_2-\mu_1^2)\}}\\ &\quad -\frac{\sigma^2_{y|x}\mu_1^2}{n_s m\sigma_w^2\{c(\mu_2-\mu_1^2)+dm(\tau_2-\mu_1^2)+en_s m(\eta_2-\mu_1^2)\}}\\ &= \frac{\sigma^2_{y|x}}{n_s m\sigma_w^2}\times\frac{1}{c+dm+en_s m}\\ &= \frac{\sigma^2_{y|x}}{n_s m\sigma_w^2}\times\lambda_3\end{aligned}$$

E.1.2 Subcluster-level randomization

When considering the randomization at the subcluster level, we could use the following model to analyze individual outcomes,

$$Y_{ijk}=\beta_1+\beta_2W_{ij}+\beta_3X_{ijk}+\beta_4W_{ij}X_{ijk}+\gamma_i+u_{ij}+\epsilon_{ijk}.$$

The upper-left block of $\sigma^2_{y|x}\boldsymbol{U}^{-1}$ is $\sigma^2_{y|x}(\boldsymbol{A}-\boldsymbol{B}\boldsymbol{D}^{-1}\boldsymbol{C})^{-1}$:

$$\lim_{n_c\to\infty} n_c \text{var}(\widehat{b}_2)=\lim_{n_c\to\infty} n_c \text{var}(\widehat{\beta}_2)=\frac{\sigma^2_{y|x}(c\mu_2+dm\tau_2)}{n_s m\sigma_w^2(c+dm)\{c(\mu_2-\mu_1^2)+dm(\tau_2-\mu_1^2)\}}$$

The lower-left block of $\sigma^2_{y|x}\boldsymbol{U}^{-1}$ is $-\sigma^2_{y|x}\boldsymbol{D}^{-1}\boldsymbol{C}(\boldsymbol{A}-\boldsymbol{B}\boldsymbol{D}^{-1}\boldsymbol{C})^{-1}$:

$$\lim_{n_c\to\infty} n_c \text{cov}(\widehat{b}_2,\widehat{b}_4)=\lim_{n_c\to\infty} n_c \text{cov}(\widehat{\beta}_2,\widehat{\beta}_4)=-\frac{\sigma^2_{y|x}\mu_1}{n_s m\sigma_w^2\{c(\mu_2-\mu_1^2)+dm(\tau_2-\mu_1^2)\}}$$

Finally, the scaled variance for the average treatment effect is

$$\begin{aligned}\lim_{n_c\to\infty} n_c \text{var}(\widehat{\beta}_2+\mu_1\widehat{\beta}_4) &= \lim_{n_c\to\infty} n_c \text{var}(\widehat{\beta}_2) + \lim_{n_c\to\infty} n_c\mu_1^2 \text{var}(\widehat{\beta}_4) + 2\lim_{n_c\to\infty} n_c\mu_1 \text{cov}(\widehat{\beta}_2,\widehat{\beta}_4)\\ &= \frac{\sigma^2_{y|x}(c\mu_2+dm\tau_2)}{n_s m\sigma_w^2(c+dm)\{c(\mu_2-\mu_1^2)+dm(\tau_2-\mu_1^2)\}}-\frac{\sigma^2_{y|x}\mu_1^2}{n_s m\sigma_w^2\{c(\mu_2-\mu_1^2)+dm(\tau_2-\mu_1^2)\}}\\ &= \frac{\sigma^2_{y|x}}{n_s m\sigma_w^2}\times\frac{1}{c+dm}\\ &= \frac{\sigma^2_{y|x}}{n_s m\sigma_w^2}\times\lambda_2\end{aligned}$$

E.1.3 Participant-level randomization

When considering the randomization at the participant level, we could use the following model to analyze individual outcomes,

$$Y_{ijk}=\beta_1+\beta_2W_{ijk}+\beta_3X_{ijk}+\beta_4W_{ijk}X_{ijk}+\gamma_i+u_{ij}+\epsilon_{ijk}.$$

The upper-left block of $\sigma^2_{y|x}\boldsymbol{U}^{-1}$ is $\sigma^2_{y|x}(\boldsymbol{A}-\boldsymbol{B}\boldsymbol{D}^{-1}\boldsymbol{C})^{-1}$:

$$\lim_{n_c\to\infty} n_c \text{var}(\widehat{b}_2) = \lim_{n_c\to\infty} n_c \text{var}(\widehat{\beta}_2) = \frac{\sigma^2_{y|x}\mu_2}{n_s m \sigma^2_w c(\mu_2-\mu_1^2)}$$

The lower-left block of $\sigma^2_{y|x}\boldsymbol{U}^{-1}$ is $-\sigma^2_{y|x}\boldsymbol{D}^{-1}\boldsymbol{C}(\boldsymbol{A}-\boldsymbol{B}\boldsymbol{D}^{-1}\boldsymbol{C})^{-1}$:

$$\lim_{n_c\to\infty} n_c \text{cov}(\widehat{b}_2,\widehat{b}_4) = \lim_{n_c\to\infty} n_c \text{cov}(\widehat{\beta}_2,\widehat{\beta}_4) = -\frac{\sigma^2_{y|x}\mu_1}{n_s m \sigma^2_w c(\mu_2-\mu_1^2)}$$

Finally, the scaled variance for the average treatment effect is

$$\begin{aligned}
\lim_{n_c\to\infty} n_c \text{var}(\widehat{\beta}_2+\mu_1\widehat{\beta}_4) &= \lim_{n_c\to\infty} n_c \text{var}(\widehat{\beta}_2) + \lim_{n_c\to\infty} n_c\mu_1^2 \text{var}(\widehat{\beta}_4) + 2\lim_{n_c\to\infty} n_c\mu_1 \text{cov}(\widehat{\beta}_2,\widehat{\beta}_4) \\
&= \frac{\sigma^2_{y|x}\mu_2}{n_s m \sigma^2_w c(\mu_2-\mu_1^2)} - \frac{\sigma^2_{y|x}\mu_1^2}{n_s m \sigma^2_w c(\mu_2-\mu_1^2)} \\
&= \frac{\sigma^2_{y|x}}{n_s m \sigma^2_w} \times \frac{1}{c} \\
&= \frac{\sigma^2_{y|x}}{n_s m \sigma^2_w} \times \lambda_1
\end{aligned}$$

E.2 Multivariate effect modifiers

E.2.1 Cluster-level randomization

When considering the randomization at the cluster level, we could use the following model to analyze individual outcomes,

$$Y_{ijk} = \beta_1 + \beta_2 W_i + \boldsymbol{\beta}_3^T \boldsymbol{X}_{ijk} + \boldsymbol{\beta}_4^T W_i \boldsymbol{X}_{ijk} + \gamma_i + u_{ij} + \epsilon_{ijk}.$$

The upper-left block of $\sigma^2_{y|x}\boldsymbol{U}^{-1}$ is given by $\sigma^2_{y|x}(\boldsymbol{A}-\boldsymbol{B}\boldsymbol{D}^{-1}\boldsymbol{C})^{-1}$, and the lower-right entry of it becomes

$$\begin{aligned}
\lim_{n_c\to\infty} n_c \text{var}(\widehat{b}_2) &= \lim_{n_c\to\infty} n_c \text{var}(\widehat{\beta}_2) \\
&= \frac{\sigma^2_{y|x}}{n_s m \sigma^2_w (c+dm+en_s m)} \times \frac{1}{1-(c+dm+en_s m)\boldsymbol{\mu}_1^T(c\boldsymbol{M}_2+dm\boldsymbol{\Upsilon}_2+en_s m\boldsymbol{H}_2)^{-1}\boldsymbol{\mu}_1}.
\end{aligned}$$

The lower-left block of $\sigma^2_{y|x}\boldsymbol{U}^{-1}$ is given by $-\sigma^2_{y|x}\boldsymbol{D}^{-1}\boldsymbol{C}(\boldsymbol{A}-\boldsymbol{B}\boldsymbol{D}^{-1}\boldsymbol{C})^{-1}$, which will be the same as the upper-right block in this case. The lower-right element can then be given as

$$\begin{aligned}
\lim_{n_c\to\infty} n_c \text{cov}(\widehat{b}_2,\widehat{\boldsymbol{b}}_4) &= \lim_{n_c\to\infty} n_c \text{cov}(\widehat{\beta}_2,\widehat{\boldsymbol{\beta}}_4) \\
&= -\frac{\sigma^2_{y|x}}{n_s m \sigma^2_w} \times \frac{(c\boldsymbol{M}_2+dm\boldsymbol{\Upsilon}_2+en_s m\boldsymbol{H}_2)^{-1}\boldsymbol{\mu}_1}{1-(c+dm+en_s m)\boldsymbol{\mu}_1^T(c\boldsymbol{M}_2+dm\boldsymbol{\Upsilon}_2+en_s m\boldsymbol{H}_2)^{-1}\boldsymbol{\mu}_1}.
\end{aligned}$$

The lower-right block of $\sigma^2_{y|x}\boldsymbol{U}^{-1}$ is $\sigma^2_{y|x}(\boldsymbol{D}-\boldsymbol{C}\boldsymbol{A}^{-1}\boldsymbol{B})^{-1}$, and its lower-right $p\times p$ block is

$$\begin{aligned}\lim_{n_c\to\infty} n_c\text{cov}(\widehat{\boldsymbol{b}}_4) &= \lim_{n_c\to\infty} n_c\text{cov}(\widehat{\boldsymbol{\beta}}_4)\\ &= \frac{\sigma^2_{y|x}}{n_s m\sigma^2_w}\left\{c\boldsymbol{M}_2 + dm\boldsymbol{\Upsilon}_2 + en_s m\boldsymbol{H}_2 - (c+dm+en_s m)\boldsymbol{\mu}_1\boldsymbol{\mu}_1^T\right\}^{-1}\end{aligned}$$

Finally, the limit variance associated with the average treatment effect estimator is given by

$$\begin{aligned}&\lim_{n_c\to\infty} n_c\text{var}(\widehat{\beta}_2+\widehat{\boldsymbol{\beta}}_4^T\boldsymbol{\mu}_1) = \lim_{n_c\to\infty} n_c\text{var}(\widehat{\beta}_2) + \lim_{n_c\to\infty} n_c\boldsymbol{\mu}_1^T\text{cov}(\widehat{\boldsymbol{\beta}}_4)\boldsymbol{\mu}_1 + 2\lim_{n_c\to\infty} n_c\boldsymbol{\mu}_1^T\text{cov}(\widehat{\beta}_2,\widehat{\boldsymbol{\beta}}_4)\\
=&\frac{\sigma^2_{y|x}}{n_s m\sigma^2_w(c+dm+en_s m)}\times\frac{1}{1-(c+dm+en_s m)\boldsymbol{\mu}_1^T(c\boldsymbol{M}_2+dm\boldsymbol{\Upsilon}_2+en_s m\boldsymbol{H}_2)^{-1}\boldsymbol{\mu}_1}\\
&+\frac{\sigma^2_{y|x}}{n_s m\sigma^2_w}\times\boldsymbol{\mu}_1^T\left\{c\boldsymbol{M}_2+dm\boldsymbol{\Upsilon}_2+en_s m\boldsymbol{H}_2-(c+dm+en_s m)\boldsymbol{\mu}_1\boldsymbol{\mu}_1^T\right\}^{-1}\boldsymbol{\mu}_1\\
&-\frac{2\sigma^2_{y|x}}{n_s m\sigma^2_w}\times\frac{\boldsymbol{\mu}_1^T(c\boldsymbol{M}_2+dm\boldsymbol{\Upsilon}_2+en_s m\boldsymbol{H}_2)^{-1}\boldsymbol{\mu}_1}{1-(c+dm+en_s m)\boldsymbol{\mu}_1^T(c\boldsymbol{M}_2+dm\boldsymbol{\Upsilon}_2+en_s m\boldsymbol{H}_2)^{-1}\boldsymbol{\mu}_1}\\
=&\frac{\sigma^2_{y|x}}{n_s m\sigma^2_w}\times\frac{1}{c+dm+en_s m}\\
=&\frac{\sigma^2_{y|x}}{n_s m\sigma^2_w}\times\lambda_3\end{aligned}$$

E.2.2 Subcluster-level randomization

When considering the randomization at the subcluster level, we could use the following model to analyze individual outcomes,

$$Y_{ijk} = \beta_1 + \beta_2 W_{ij} + \boldsymbol{\beta}_3^T\boldsymbol{X}_{ijk} + \boldsymbol{\beta}_4^T W_{ij}\boldsymbol{X}_{ijk} + \gamma_i + u_{ij} + \epsilon_{ijk}.$$

The upper-left block of $\sigma^2_{y|x}\boldsymbol{U}^{-1}$ is given by $\sigma^2_{y|x}(\boldsymbol{A}-\boldsymbol{B}\boldsymbol{D}^{-1}\boldsymbol{C})^{-1}$, and the lower-right entry of it becomes

$$n_c\text{var}(\widehat{b}_2) = n_c\text{var}(\widehat{\beta}_2) = \frac{\sigma^2_{y|x}}{n_s m\sigma^2_w(c+dm)}\times\frac{1}{1-(c+dm)\boldsymbol{\mu}_1^T(c\boldsymbol{M}_2+dm\boldsymbol{\Upsilon}_2)^{-1}\boldsymbol{\mu}_1}.$$

The lower-left block of $\sigma^2_{y|x}\boldsymbol{U}^{-1}$ is given by $-\sigma^2_{y|x}\boldsymbol{D}^{-1}\boldsymbol{C}(\boldsymbol{A}-\boldsymbol{B}\boldsymbol{D}^{-1}\boldsymbol{C})^{-1}$, which will be the same as the upper-right block in this case. The lower-right element can then be given as

$$n_c\text{cov}(\widehat{b}_2,\widehat{\boldsymbol{b}}_4) = n_c\text{cov}(\widehat{\beta}_2,\widehat{\boldsymbol{\beta}}_4) = -\frac{\sigma^2_{y|x}}{n_s m\sigma^2_w}\times\frac{(c\boldsymbol{M}_2+dm\boldsymbol{\Upsilon}_2)^{-1}\boldsymbol{\mu}_1}{1-(c+dm)\boldsymbol{\mu}_1^T(c\boldsymbol{M}_2+dm\boldsymbol{\Upsilon}_2)^{-1}\boldsymbol{\mu}_1}.$$

The lower-right block of $\sigma^2_{y|x}\boldsymbol{U}^{-1}$ is $\sigma^2_{y|x}(\boldsymbol{D}-\boldsymbol{C}\boldsymbol{A}^{-1}\boldsymbol{B})^{-1}$, and its lower-right $p\times p$ block is

$$n_c\text{cov}(\widehat{\boldsymbol{b}}_4) = n_c\text{cov}(\widehat{\boldsymbol{\beta}}_4) = \frac{\sigma^2_{y|x}}{n_s m\sigma^2_w}\left\{c\boldsymbol{M}_2+dm\boldsymbol{\Upsilon}_2-(c+dm)\boldsymbol{\mu}_1\boldsymbol{\mu}_1^T\right\}^{-1}$$

Finally, the scaled variance for the average treatment effect is given by

$$
\begin{aligned}
&n_c \mathrm{var}(\widehat{\beta}_2 + \widehat{\boldsymbol{\beta}}_4^T \boldsymbol{\mu}_1) = n_c \mathrm{var}(\widehat{\beta}_2) + n_c \boldsymbol{\mu}_1^T \mathrm{cov}(\widehat{\boldsymbol{\beta}}_4) \boldsymbol{\mu}_1 + 2 n_c \boldsymbol{\mu}_1^T \mathrm{cov}(\widehat{\beta}_2, \widehat{\boldsymbol{\beta}}_4) \\
=& \frac{\sigma_{y|x}^2}{n_s m \sigma_w^2 (c + dm)} \times \frac{1}{1 - (c+dm)\boldsymbol{\mu}_1^T (c\boldsymbol{M}_2 + dm\boldsymbol{\Upsilon}_2)^{-1} \boldsymbol{\mu}_1} \\
&+ \frac{\sigma_{y|x}^2}{n_s m \sigma_w^2} \times \boldsymbol{\mu}_1^T \left\{ c\boldsymbol{M}_2 + dm\boldsymbol{\Upsilon}_2 - (c+dm)\boldsymbol{\mu}_1 \boldsymbol{\mu}_1^T \right\}^{-1} \boldsymbol{\mu}_1 \\
&- \frac{2\sigma_{y|x}^2}{n_s m \sigma_w^2} \times \frac{\boldsymbol{\mu}_1^T (c\boldsymbol{M}_2 + dm\boldsymbol{\Upsilon}_2)^{-1} \boldsymbol{\mu}_1}{1 - (c+dm)\boldsymbol{\mu}_1^T (c\boldsymbol{M}_2 + dm\boldsymbol{\Upsilon}_2)^{-1} \boldsymbol{\mu}_1} \\
=& \frac{\sigma_{y|x}^2}{n_s m \sigma_w^2} \times \frac{1}{c + dm} \\
=& \frac{\sigma_{y|x}^2}{n_s m \sigma_w^2} \times \lambda_2
\end{aligned}
$$

E.2.3 Participant-level randomization

When considering the randomization at the participant level, we could use the following model to analyze individual outcomes,

$$Y_{ijk} = \beta_1 + \beta_2 W_{ijk} + \boldsymbol{\beta}_3^T \boldsymbol{X}_{ijk} + \boldsymbol{\beta}_4^T W_{ijk} \boldsymbol{X}_{ijk} + \gamma_i + u_{ij} + \epsilon_{ijk}.$$

The upper-left block of $\sigma_{y|x}^2 \boldsymbol{U}^{-1}$ is given by $\sigma_{y|x}^2 (\boldsymbol{A} - \boldsymbol{B}\boldsymbol{D}^{-1}\boldsymbol{C})^{-1}$, and the lower-right entry of it becomes

$$n_c \mathrm{var}(\widehat{b}_2) = n_c \mathrm{var}(\widehat{\beta}_2) = \frac{\sigma_{y|x}^2}{n_s m \sigma_w^2 c} \times \frac{1}{1 - \boldsymbol{\mu}_1^T \boldsymbol{M}_2^{-1} \boldsymbol{\mu}_1}.$$

The lower-left block of $\sigma_{y|x}^2 \boldsymbol{U}^{-1}$ is given by $-\sigma_{y|x}^2 \boldsymbol{D}^{-1}\boldsymbol{C}(\boldsymbol{A} - \boldsymbol{B}\boldsymbol{D}^{-1}\boldsymbol{C})^{-1}$, which will be the same as the upper-right block in this case. The lower-right element can then be given as

$$n_c \mathrm{cov}(\widehat{b}_2, \widehat{\boldsymbol{b}}_4) = n_c \mathrm{cov}(\widehat{\beta}_2, \widehat{\boldsymbol{\beta}}_4) = -\frac{\sigma_{y|x}^2}{n_s m \sigma_w^2 c} \times \frac{\boldsymbol{M}_2^{-1} \boldsymbol{\mu}_1}{1 - \boldsymbol{\mu}_1^T \boldsymbol{M}_2^{-1} \boldsymbol{\mu}_1}.$$

The lower-right block of $\sigma_{y|x}^2 \boldsymbol{U}^{-1}$ is $\sigma_{y|x}^2 (\boldsymbol{D} - \boldsymbol{C}\boldsymbol{A}^{-1}\boldsymbol{B})^{-1}$, and its lower-right $p \times p$ block is

$$n_c \mathrm{cov}(\widehat{\boldsymbol{b}}_4) = n_c \mathrm{cov}(\widehat{\boldsymbol{\beta}}_4) = \frac{\sigma_{y|x}^2}{n_s m \sigma_w^2 c} \left\{ \boldsymbol{M}_2 - \boldsymbol{\mu}_1 \boldsymbol{\mu}_1^T \right\}^{-1}$$

Finally, the scaled variance for the average treatment effect is given by

$$
\begin{aligned}
& n_c \text{var}(\widehat{\beta}_2 + \widehat{\boldsymbol{\beta}}_4^T \boldsymbol{\mu}_1) = n_c \text{var}(\widehat{\beta}_2) + n_c \boldsymbol{\mu}_1^T \text{cov}(\widehat{\boldsymbol{\beta}}_4) \boldsymbol{\mu}_1 + 2 n_c \boldsymbol{\mu}_1^T \text{cov}(\widehat{\beta}_2, \widehat{\boldsymbol{\beta}}_4) \\
=& \frac{\sigma^2_{y|x}}{n_s m \sigma^2_w c} \times \frac{1}{1 - \boldsymbol{\mu}_1^T \boldsymbol{M}_2^{-1} \boldsymbol{\mu}_1} + \frac{\sigma^2_{y|x}}{n_s m \sigma^2_w c} \times \boldsymbol{\mu}_1^T \left\{ \boldsymbol{M}_2 - \boldsymbol{\mu}_1 \boldsymbol{\mu}_1^T \right\}^{-1} \boldsymbol{\mu}_1 \\
& - \frac{2\sigma^2_{y|x}}{n_s m \sigma^2_w c} \times \frac{\boldsymbol{\mu}_1^T \boldsymbol{M}_2^{-1} \boldsymbol{\mu}_1}{1 - \boldsymbol{\mu}_1^T \boldsymbol{M}_2^{-1} \boldsymbol{\mu}_1} \\
=& \frac{\sigma^2_{y|x}}{n_s m \sigma^2_w} \times \frac{1}{c} \\
=& \frac{\sigma^2_{y|x}}{n_s m \sigma^2_w} \times \lambda_1
\end{aligned}
$$

Web Appendix F Additional technical details for the numerical studies

F.1 Approximate marginal variance and marginal outcome-ICC parameters in the unadjusted linear mixed model

Recall the conditional outcome model with univariate participant-level effect modifier X_{ijk} is defined as

$$
Y_{ijk} = \beta_1 + \beta_2 W_i + \beta_3 X_{ijk} + \beta_4 W_i X_{ijk} + \gamma_i + u_{ij} + \epsilon_{ijk},
$$

where $\gamma_i \sim \mathcal{N}(0, \sigma^2_\gamma)$, $u_{ij} \sim \mathcal{N}(0, \sigma^2_u)$, $\epsilon_{ijk} \sim \mathcal{N}(0, \sigma^2_\epsilon)$, and assume random effects are mutually independent.

In our simulation study, we assume $X_{ijk} = \mu + a_i + b_{ij} + c_{ijk}$, where $a_i \sim \mathcal{N}(0, \sigma^2_a)$, $b_{ij} \sim \mathcal{N}(0, \sigma^2_b)$, and $c_{ijk} \sim \mathcal{N}(0, \sigma^2_c)$. The marginal variance of X_{ijk} is therefore $\sigma^2_x = \sigma^2_a + \sigma^2_b + \sigma^2_c$, and the marginal within-subcluster covariate-ICC is $\rho_0 = (\sigma^2_a + \sigma^2_b)/\sigma^2_x$, the marginal between-subcluster covariate-ICC is $\rho_1 = \sigma^2_a/\sigma^2_x$. In this case, we can expand the original conditional outcome model as

$$
\begin{aligned}
Y_{ijk} &= \beta_1 + \beta_2 W_i + \beta_3(\mu + a_i + b_{ij} + c_{ijk}) + \beta_4 W_i(\mu + a_i + b_{ij} + c_{ijk}) + \gamma_i + u_{ij} + \epsilon_{ijk} \\
&= (\beta_1 + \beta_3 \mu) + (\beta_2 + \beta_4 \mu) W_i + (\beta_3 a_i + \beta_4 W_i a_i + \gamma_i) + (\beta_3 b_{ij} + \beta_4 W_i b_{ij} + u_{ij}) \\
&\quad + (\beta_3 c_{ijk} + \beta_4 W_i c_{ijk} + \epsilon_{ijk}) \\
&= \xi_1 + \xi_2 W_i + A_i + B_{ij} + C_{ijk}
\end{aligned}
$$

where $\xi_1 = \beta_1 + \beta_3 \mu$, $\xi_2 = \beta_2 + \beta_4 \mu$, $A_i = \beta_3 a_i + \beta_4 W_i a_i + \gamma_i$, $B_{ij} = \beta_3 b_{ij} + \beta_4 W_i b_{ij} + u_{ij}$, and $C_{ijk} = \beta_3 c_{ijk} + \beta_4 W_i c_{ijk} + \epsilon_{ijk}$.

In what follows, we assume $\mu = 0$, which suggests $\xi_1 = \beta_1$ and $\xi_2 = \beta_2$. In other words, we can interpret the main effect of W_i as the average treatment effect. Let $W_i \sim \text{Bernoulli}(\overline{W})$, since

$W_i = W_i^2$ and the random intercepts are all independent, we have

$$(A_i|W_i) \sim \mathcal{N}\left(0, (\beta_3^2 + \beta_4^2 W_i + 2\beta_3\beta_4 W_i)\sigma_a^2 + \sigma_\gamma^2\right),$$
$$(B_{ij}|W_i) \sim \mathcal{N}\left(0, (\beta_3^2 + \beta_4^2 W_i + 2\beta_3\beta_4 W_i)\sigma_b^2 + \sigma_u^2\right),$$
$$(C_{ijk}|W_i) \sim \mathcal{N}\left(0, (\beta_3^2 + \beta_4^2 W_i + 2\beta_3\beta_4 W_i)\sigma_c^2 + \sigma_\epsilon^2\right).$$

By the law of total variance,

$$\begin{aligned}
\sigma_A^2 &= \text{var}(A_i) = \mathbb{E}_W\{\text{Var}(A_i|W_i)\} + \text{var}_W\{\mathbb{E}(A_i|W_i)\} \\
&= \mathbb{E}_W\left\{(\beta_3^2 + \beta_4^2 W_i + 2\beta_3\beta_4 W_i)\sigma_a^2 + \sigma_\gamma^2\right\} \\
&= (\beta_3^2 + \beta_4^2\overline{W} + 2\beta_3\beta_4\overline{W})\sigma_a^2 + \sigma_\gamma^2, \\
\sigma_B^2 &= \text{var}(B_{ij}) = \mathbb{E}_W\{\text{var}(B_{ij}|W_i)\} + \text{var}_W\{\mathbb{E}(B_{ij}|W_i)\} \\
&= \mathbb{E}_W\left\{(\beta_3^2 + \beta_4^2 W_i + 2\beta_3\beta_4 W_i)\sigma_b^2 + \sigma_u^2\right\} \\
&= (\beta_3^2 + \beta_4^2\overline{W} + 2\beta_3\beta_4\overline{W})\sigma_b^2 + \sigma_u^2, \\
\sigma_C^2 &= \text{var}(C_{ijk}) = \mathbb{E}_W\{\text{Var}(C_{ijk}|W_i)\} + \text{var}_W\{\mathbb{E}(C_{ijk}|W_i)\} \\
&= \mathbb{E}_W\left\{(\beta_3^2 + \beta_4^2 W_i + 2\beta_3\beta_4 W_i)\sigma_c^2 + \sigma_\epsilon^2\right\} \\
&= (\beta_3^2 + \beta_4^2\overline{W} + 2\beta_3\beta_4\overline{W})\sigma_c^2 + \sigma_\epsilon^2, \\
\sigma_{A,B} &= \text{cov}(A_i, B_{ij}) = \mathbb{E}_W\{\text{cov}(A_i, B_{ij}|W_i)\} + \text{cov}_W\{\mathbb{E}(A_i|W_i), \mathbb{E}(B_{ij}|W_i)\} \\
&= 0 = \sigma_{A,C} = \sigma_{B,C}.
\end{aligned}$$

Define the unadjusted variance $\sigma_y^2 = \text{var}(A_i + B_{ij} + C_{ijk}) = \sigma_A^2 + \sigma_B^2 + \sigma_C^2$, and write $Q = \beta_3^2 + \beta_4^2\overline{W} + 2\beta_3\beta_4\overline{W}$, then the unadjusted within-subcluster outcome-ICC is

$$\begin{aligned}
\widetilde{\alpha}_0 = \frac{\sigma_A^2 + \sigma_B^2}{\sigma_y^2} &= \frac{\sigma_\gamma^2 + \sigma_u^2 + Q(\sigma_a^2 + \sigma_b^2)}{\sigma_\gamma^2 + \sigma_u^2 + \sigma_\epsilon^2 + Q(\sigma_a^2 + \sigma_b^2 + \sigma_c^2)} \\
&= \frac{\sigma_{y|x}^2}{\sigma_{y|x}^2 + Q\sigma_x^2} \times \frac{\sigma_\gamma^2 + \sigma_u^2}{\sigma_{y|x}^2} + \frac{Q\sigma_x^2}{\sigma_{y|x}^2 + Q\sigma_x^2} \times \frac{\sigma_a^2 + \sigma_b^2}{\sigma_x^2} \\
&= \frac{\sigma_{y|x}^2}{\sigma_{y|x}^2 + Q\sigma_x^2}\alpha_0 + \frac{Q\sigma_x^2}{\sigma_{y|x}^2 + Q\sigma_x^2}\rho_0 \\
&= w\alpha_0 + (1-w)\rho_0,
\end{aligned}$$

and the unadjusted between-subcluster outcome-ICC is

$$\begin{aligned}
\widetilde{\alpha}_1 = \frac{\sigma_A^2}{\sigma_y^2} &= \frac{\sigma_\gamma^2 + Q\sigma_a^2}{\sigma_\gamma^2 + \sigma_u^2 + \sigma_\epsilon^2 + Q(\sigma_a^2 + \sigma_b^2 + \sigma_c^2)} \\
&= \frac{\sigma_{y|x}^2}{\sigma_{y|x}^2 + Q\sigma_x^2} \times \frac{\sigma_\gamma^2}{\sigma_{y|x}^2} + \frac{Q\sigma_x^2}{\sigma_{y|x}^2 + Q\sigma_x^2} \times \frac{\sigma_a^2}{\sigma_x^2} \\
&= \frac{\sigma_{y|x}^2}{\sigma_{y|x}^2 + Q\sigma_x^2}\alpha_1 + \frac{Q\sigma_x^2}{\sigma_{y|x}^2 + Q\sigma_x^2}\rho_1 \\
&= w\alpha_1 + (1-w)\rho_1,
\end{aligned}$$

where $w = \sigma^2_{y|x}/(\sigma^2_{y|x} + Q\sigma^2_x)$.

F.2 Comparison with the sample size method in Dong et al. (2018)

In Table 1 of the main manuscript, we compared the sample size estimated from our proposed method with those obtained using the method of Dong et al. (2018) (referred to as nonrandom varying slope model in their article), who also assumed randomization at the cluster level. With a participant-level effect modifier, the formula in Dong et al. (2018) depends on the R_1^2, which is the proportion of variance at the participant level that is explained by the effect modifier X_{ijk}. Resuming the above notation, to ensure a common basis for sample size estimate comparisons, we can use their definition and approximate

$$R_1^2 \approx 1 - \sigma_\epsilon^2/\sigma_C^2 = 1 - \frac{\sigma_\epsilon^2}{(\beta_3^2 + \beta_4^2\overline{W} + 2\beta_3\beta_4\overline{W})\sigma_c^2 + \sigma_\epsilon^2} = 1 - \frac{\sigma_\epsilon^2}{Q\sigma_c^2 + \sigma_\epsilon^2}.$$

We also notice that the outcome-ICC parameters are defined slightly differently from our paper: their unconditional level-3 ICC (ICC_3) is approximated by $\widetilde{\alpha}_1$ and their level-2 ICC (ICC_2) is $\widetilde{\alpha}_0 - \widetilde{\alpha}_1$. Finally, their standardized effect size is given by $\delta = \beta_4/\sigma_y^2 = \beta_4/(\sigma_A^2 + \sigma_B^2 + \sigma_C^2)$. In each scenario, we compute the above transformed design parameters and plug in the `PowerUp!-Moderator` software (in Microsoft Excel) on https://www.causalevaluation.org/power-analysis.html to determine the required sample size $\widehat{n}_c^*$ in Table 1. A screenshot of the first scenario is given below.

Model CRA3-1NC: Power Calculator for Three-Level Cluster Random Assignment Design — Treatment at Level 3 and Continuous Moderator at Level 1 (Nonrandomly varying moderator slope model)

Assumptions		Comments
Alpha Level (α)	0.050	Probability of a Type I error
Two-tailed or One-tailed Test?	2.000	
Effect Size Difference	0.095	Effect Size Difference regarding standardized coefficient
Rho_3 (ICC_3)	0.019	Proportion of variance among Level 3 units (V3/(V1 + V2 + V3))
Rho_2 (ICC_2)	0.009	Proportion of variance among Level 2 units (V2/(V1 + V2 + V3))
P	0.500	Proportion of Level 3 units randomized to treatment: K_T / (K_T + K_C)
R_1^2	0.084	Proportion of variance in Level 1 outcome explained by Level 1 covariates
g_1^*	0.000	Number of Level 1 covariates excluding the moderator
n (Average Sample Size for Level 1)	20.000	Mean number of Level 1 units per Level 2 unit (harmonic mean recommended)
J (Average Sample Size for Level 2)	4.000	Mean number of Level 2 units per Level 3 unit (harmonic mean recommended)
K (Sample Size [# of Level 3 units])	40.000	Number of Level 3 units
Noncentrality Parameter	2.848	Automatically computed from the above assumptions
Power (1-β)	**0.812**	Statistical power (1-probability of a Type II error)

Note: The parameters in yellow cells need to be specified. The power will be calculated automatically.

Translating the formula in the `PowerUp!-Moderator` software (nonrandom varying slope model)

using our notation, the power of the HTE test is approximated by (Harrison and Brady, 2004)

$$\text{power} = 1 - \lambda \approx 1 - \Phi_t\left(t_{\alpha/2,\text{DoF}}; \text{DoF}, |\delta|/\sqrt{\text{var}^*(\widehat{\delta})}\right), \tag{8}$$

$$= 1 - \Phi_t\left(t_{\alpha/2,\text{DoF}}; \text{DoF}, |\beta_4|/\sqrt{\text{var}^*(\widehat{\beta}_4)}\right) \tag{9}$$

where $t_{\alpha/2,\text{DoF}}$ is the upper $\alpha/2$th quantile of the central t-distribution with specified degrees of freedom (DoF) and $\Phi_t(t; \text{DoF}, \Lambda)$ is the cumulative t-distribution function with DoF degrees of freedom and noncentrality parameter Λ. In Dong et al. (2018), we DoF is given by $\text{DoF} = n_c n_s m - n_c n_s - n_c - 2$, which often exceeds 30, and hence the above power formula can be well approximated by the standard normal distribution (as we have opted for using our method). The variance of the intervention effect, $\text{var}^*(\widehat{\beta}_4)$, is an implicit function of the sample size and correlation parameters, and is given in their paper by

$$\begin{aligned}
\text{var}^*(\widehat{\beta}_4) &= \frac{\sigma_y^2(1-R_1^2)\left\{1-\widetilde{\alpha}_1-(\widetilde{\alpha}_0-\widetilde{\alpha}_1)\right\}}{n_c n_s m \sigma_X^2 \overline{W}(1-\overline{W})} \\
&= (\sigma_{y|x}^2 + Q\sigma_x^2)\left\{1 - \frac{\sigma_{y|x}^2}{\sigma_{y|x}^2 + Q\sigma_x^2}\alpha_0 - \frac{Q\sigma_x^2}{\sigma_{y|x}^2 + Q\sigma_x^2}\rho_0\right\}\left\{\frac{\sigma_\epsilon^2}{Q\sigma_c^2+\sigma_\epsilon^2}\right\} \Big/ \left\{n_c n_s m \sigma_X^2 \overline{W}(1-\overline{W})\right\} \\
&= \frac{\sigma_{y|x}^2(1-\alpha_0)}{n_c n_s m \overline{W}(1-\overline{W})\sigma_X^2},
\end{aligned}$$

which turns out to be independent of the between-subcluster outcome-ICC, α_1, as well as the covariate-ICCs, ρ_0 and ρ_1. In fact, their implied variance formula of the interaction effect parameter (interpreted using our notation) coincides with $\sigma_{4,(1)}^2/(n_c n_s m)$, which is the variance expression derived in Theorem 1 when the randomization is carried out at the participant level (level 1). In fact, this explains why the column under $\widehat{n}_c^*$ in Table 1 of the main manuscript and and column under $\widehat{n}_c$ in Web Table 6 have identical elements. Due to the linear ordering statements proved in Theorem 1, $\text{var}^*(\widehat{\beta}_4)$ is no larger than that derived when randomization is carried out at the highest level and therefore their intrinsic difference in magnitude underlies the tendency to under-estimate the required sample size using the Dong et al. (2018) method (given the same level of effect size of HTE).

Web Appendix G Additional Simulation results for cluster-level, subcluster-level and participant-level randomization

Web Table 1: Comparison between the predicted and Monte Carlo variances (under the null $\mathcal{H}_0$ and the alternative $\mathcal{H}_1$ respectively) for the covariate-adjusted average treatment effect estimator in the CRT simulations. The predicted and Monte Carlo variances were both multiplied by 1000. The purpose of this Table is to check whether the predicted variance (Pred. Var) of the average treatment effect estimator based on Theorem 2.2 is still close to the Monte Carlo variance (MC Var) by simulations, despite the use of the t-distribution for hypothesis testing. Each scenario below matches that in Table 3 of the main manuscript.

Design Parameters							Performance metrics		
m	n_s	α_0	α_1	ρ_0	ρ_1	$\widehat{n}_c$	MC Var ($\mathcal{H}_0$)	MC Var ($\mathcal{H}_1$)	Pred. Var
20	4	0.015	0.010	0.150	0.100	22	4.323	4.323	4.284
20	4	0.015	0.010	0.300	0.150	22	4.332	4.332	4.284
20	4	0.015	0.010	0.500	0.300	22	4.354	4.354	4.284
20	4	0.100	0.050	0.150	0.100	60	4.930	4.930	4.917
20	4	0.100	0.050	0.300	0.150	60	4.931	4.931	4.917
20	4	0.100	0.050	0.500	0.300	60	4.935	4.935	4.917
20	8	0.015	0.010	0.150	0.100	16	4.231	4.231	4.195
20	8	0.015	0.010	0.300	0.150	16	4.239	4.239	4.195
20	8	0.015	0.010	0.500	0.300	16	4.261	4.261	4.195
20	8	0.100	0.050	0.150	0.100	52	4.763	4.763	4.760
20	8	0.100	0.050	0.300	0.150	52	4.763	4.763	4.760
20	8	0.100	0.050	0.500	0.300	52	4.766	4.766	4.760
50	4	0.015	0.010	0.150	0.100	16	4.073	4.073	4.044
50	4	0.015	0.010	0.300	0.150	16	4.080	4.081	4.044
50	4	0.015	0.010	0.500	0.300	16	4.100	4.100	4.044
50	4	0.100	0.050	0.150	0.100	56	4.782	4.781	4.786
50	4	0.100	0.050	0.300	0.150	56	4.782	4.782	4.786
50	4	0.100	0.050	0.500	0.300	56	4.783	4.785	4.786
50	8	0.015	0.010	0.150	0.100	14	3.765	3.765	3.739
50	8	0.015	0.010	0.300	0.150	14	3.770	3.770	3.739
50	8	0.015	0.010	0.500	0.300	14	3.785	3.785	3.739
50	8	0.100	0.050	0.150	0.100	48	4.881	4.879	4.875
50	8	0.100	0.050	0.300	0.150	48	4.877	4.875	4.875
50	8	0.100	0.050	0.500	0.300	48	4.882	4.882	4.875

Web Table 2: Estimated required number of clusters $\widehat{n}_c$ for the unadjusted average treatment effect test based on the Cunningham and Johnson (2016) formula, empirical type I error (Emp. Size), empirical power (Emp. Power), as well as predicted power (Pred. Power) for the unadjusted average treatment effect test, when randomization is at the cluster level. For studying power, the average treatment effect size is fixed at $\Delta_{\text{ATE}} = 0.2$.

Design Parameters									Performance Characteristics		
m	n_s	α_0	α_1	ρ_0	ρ_1	$\widetilde{\alpha}_0$	$\widetilde{\alpha}_1$	$\widehat{n}_c$	Emp. Size	Emp. Power	Pred. Power
20	4	0.015	0.010	0.150	0.100	0.028	0.019	32	0.054	0.816	0.816
20	4	0.015	0.010	0.300	0.150	0.042	0.023	38	0.052	0.813	0.812
20	4	0.015	0.010	0.500	0.300	0.062	0.038	52	0.043	0.818	0.814
20	4	0.100	0.050	0.150	0.100	0.105	0.055	72	0.047	0.806	0.811
20	4	0.100	0.050	0.300	0.150	0.119	0.060	78	0.052	0.813	0.809
20	4	0.100	0.050	0.500	0.300	0.138	0.074	90	0.050	0.801	0.802
20	8	0.015	0.010	0.150	0.100	0.028	0.019	26	0.051	0.826	0.824
20	8	0.015	0.010	0.300	0.150	0.042	0.023	30	0.048	0.796	0.805
20	8	0.015	0.010	0.500	0.300	0.062	0.038	44	0.053	0.821	0.814
20	8	0.100	0.050	0.150	0.100	0.105	0.055	60	0.046	0.801	0.801
20	8	0.100	0.050	0.300	0.150	0.119	0.060	66	0.053	0.803	0.806
20	8	0.100	0.050	0.500	0.300	0.138	0.074	78	0.051	0.797	0.801
50	4	0.015	0.010	0.150	0.100	0.028	0.019	26	0.052	0.829	0.825
50	4	0.015	0.010	0.300	0.150	0.042	0.023	32	0.048	0.819	0.818
50	4	0.015	0.010	0.500	0.300	0.062	0.038	46	0.050	0.817	0.817
50	4	0.100	0.050	0.150	0.100	0.105	0.055	66	0.051	0.815	0.810
50	4	0.100	0.050	0.300	0.150	0.119	0.060	72	0.041	0.816	0.808
50	4	0.100	0.050	0.500	0.300	0.138	0.074	84	0.049	0.799	0.800
50	8	0.015	0.010	0.150	0.100	0.028	0.019	22	0.047	0.814	0.812
50	8	0.015	0.010	0.300	0.150	0.042	0.023	28	0.054	0.816	0.822
50	8	0.015	0.010	0.500	0.300	0.062	0.038	40	0.048	0.803	0.805
50	8	0.100	0.050	0.150	0.100	0.105	0.055	58	0.051	0.803	0.807
50	8	0.100	0.050	0.300	0.150	0.119	0.060	64	0.054	0.807	0.812
50	8	0.100	0.050	0.500	0.300	0.138	0.074	76	0.052	0.815	0.805

Web Table 3: Estimated required number of clusters $\widehat{n}_c$ for the HTE test based on the proposed formula, empirical type I error (Emp. Size), empirical power (Emp. Power), as well as predicted power (Pred. Power) for the HTE, test when randomization is at the subcluster level. For studying power, the HTE effect size is fixed at $\Delta_{\text{HTE}} = 0.1$.

Design Parameters							Performance Characteristics		
m	n_s	α_0	α_1	ρ_0	ρ_1	$\widehat{n}_c$	Emp. Size	Emp. Power	Pred. Power
20	4	0.015	0.010	0.150	0.100	40	0.051	0.802	0.806
20	4	0.015	0.010	0.300	0.150	40	0.051	0.792	0.801
20	4	0.015	0.010	0.500	0.300	42	0.050	0.798	0.813
20	4	0.100	0.050	0.150	0.100	40	0.050	0.801	0.807
20	4	0.100	0.050	0.300	0.150	44	0.049	0.805	0.810
20	4	0.100	0.050	0.500	0.300	50	0.050	0.796	0.809
20	8	0.015	0.010	0.150	0.100	20	0.051	0.794	0.806
20	8	0.015	0.010	0.300	0.150	20	0.052	0.785	0.801
20	8	0.015	0.010	0.500	0.300	22	0.052	0.811	0.830
20	8	0.100	0.050	0.150	0.100	20	0.055	0.797	0.807
20	8	0.100	0.050	0.300	0.150	22	0.049	0.804	0.810
20	8	0.100	0.050	0.500	0.300	26	0.057	0.814	0.824
50	4	0.015	0.010	0.150	0.100	18	0.054	0.844	0.844
50	4	0.015	0.010	0.300	0.150	18	0.054	0.821	0.833
50	4	0.015	0.010	0.500	0.300	18	0.054	0.793	0.817
50	4	0.100	0.050	0.150	0.100	18	0.050	0.842	0.842
50	4	0.100	0.050	0.300	0.150	20	0.054	0.822	0.832
50	4	0.100	0.050	0.500	0.300	24	0.047	0.821	0.823
50	8	0.015	0.010	0.150	0.100	10	0.057	0.871	0.879
50	8	0.015	0.010	0.300	0.150	10	0.055	0.853	0.869
50	8	0.015	0.010	0.500	0.300	10	0.056	0.826	0.855
50	8	0.100	0.050	0.150	0.100	10	0.051	0.872	0.877
50	8	0.100	0.050	0.300	0.150	10	0.055	0.822	0.832
50	8	0.100	0.050	0.500	0.300	12	0.050	0.816	0.823

Web Table 4: Estimated required number of clusters $\widehat{n}_c$ for the covariate-adjusted average treatment effect test based on the proposed formula, empirical type I error (Emp. Size), empirical power (Emp. Power), as well as predicted power (Pred. Power) for the covariate-adjusted average treatment effect test, when randomization is at the subcluster level. For studying power, the average treatment effect size is fixed at $\Delta_{\text{ATE}} = 0.1$.

Design Parameters							Performance Characteristics		
m	n_s	α_0	α_1	ρ_0	ρ_1	$\widehat{n}_c$	Emp. Size	Emp. Power	Pred. Power
20	4	0.015	0.010	0.150	0.100	44	0.053	0.806	0.813
20	4	0.015	0.010	0.300	0.150	44	0.053	0.806	0.813
20	4	0.015	0.010	0.500	0.300	44	0.053	0.806	0.813
20	4	0.100	0.050	0.150	0.100	76	0.055	0.791	0.807
20	4	0.100	0.050	0.300	0.150	76	0.055	0.791	0.807
20	4	0.100	0.050	0.500	0.300	76	0.054	0.792	0.807
20	8	0.015	0.010	0.150	0.100	22	0.061	0.806	0.813
20	8	0.015	0.010	0.300	0.150	22	0.061	0.805	0.813
20	8	0.015	0.010	0.500	0.300	22	0.063	0.804	0.813
20	8	0.100	0.050	0.150	0.100	38	0.051	0.804	0.807
20	8	0.100	0.050	0.300	0.150	38	0.051	0.803	0.807
20	8	0.100	0.050	0.500	0.300	38	0.051	0.802	0.807
50	4	0.015	0.010	0.150	0.100	20	0.054	0.817	0.812
50	4	0.015	0.010	0.300	0.150	20	0.056	0.815	0.812
50	4	0.015	0.010	0.500	0.300	20	0.057	0.815	0.812
50	4	0.100	0.050	0.150	0.100	54	0.046	0.800	0.805
50	4	0.100	0.050	0.300	0.150	54	0.047	0.797	0.805
50	4	0.100	0.050	0.500	0.300	54	0.047	0.800	0.805
50	8	0.015	0.010	0.150	0.100	10	0.058	0.805	0.812
50	8	0.015	0.010	0.300	0.150	10	0.061	0.804	0.812
50	8	0.015	0.010	0.500	0.300	10	0.065	0.801	0.812
50	8	0.100	0.050	0.150	0.100	28	0.053	0.814	0.819
50	8	0.100	0.050	0.300	0.150	28	0.055	0.813	0.819
50	8	0.100	0.050	0.500	0.300	28	0.056	0.813	0.819

Web Table 5: Estimated required number of clusters $\widehat{n}_c$ for the unadjusted average treatment effect test based on the Cunningham and Johnson (2016) formula, empirical type I error (Emp. Size), empirical power (Emp. Power), as well as predicted power (Pred. Power) for the unadjusted average treatment effect test, when randomization is at the subcluster level. For studying power, the average treatment effect size is fixed at $\Delta_{\text{ATE}} = 0.1$.

Design Parameters									Performance Characteristics		
m	n_s	α_0	α_1	ρ_0	ρ_1	$\widetilde{\alpha}_0$	$\widetilde{\alpha}_1$	$\widehat{n}_c$	Emp. Size	Emp. Power	Pred. Power
20	4	0.015	0.010	0.150	0.100	0.028	0.019	52	0.046	0.806	0.813
20	4	0.015	0.010	0.300	0.150	0.042	0.023	60	0.044	0.830	0.813
20	4	0.015	0.010	0.500	0.300	0.062	0.038	62	0.055	0.801	0.804
20	4	0.100	0.050	0.150	0.100	0.105	0.055	84	0.053	0.797	0.808
20	4	0.100	0.050	0.300	0.150	0.119	0.060	90	0.052	0.799	0.800
20	4	0.100	0.050	0.500	0.300	0.138	0.074	94	0.052	0.805	0.803
20	8	0.015	0.010	0.150	0.100	0.028	0.019	26	0.051	0.811	0.813
20	8	0.015	0.010	0.300	0.150	0.042	0.023	30	0.059	0.815	0.813
20	8	0.015	0.010	0.500	0.300	0.062	0.038	32	0.056	0.821	0.816
20	8	0.100	0.050	0.150	0.100	0.105	0.055	42	0.057	0.803	0.808
20	8	0.100	0.050	0.300	0.150	0.119	0.060	46	0.050	0.811	0.809
20	8	0.100	0.050	0.500	0.300	0.138	0.074	48	0.050	0.818	0.811
50	4	0.015	0.010	0.150	0.100	0.028	0.019	26	0.055	0.809	0.816
50	4	0.015	0.010	0.300	0.150	0.042	0.023	34	0.052	0.806	0.811
50	4	0.015	0.010	0.500	0.300	0.062	0.038	38	0.055	0.816	0.811
50	4	0.100	0.050	0.150	0.100	0.105	0.055	60	0.050	0.808	0.807
50	4	0.100	0.050	0.300	0.150	0.119	0.060	68	0.047	0.804	0.805
50	4	0.100	0.050	0.500	0.300	0.138	0.074	72	0.054	0.804	0.806
50	8	0.015	0.010	0.150	0.100	0.028	0.019	14	0.051	0.834	0.843
50	8	0.015	0.010	0.300	0.150	0.042	0.023	18	0.057	0.824	0.832
50	8	0.015	0.010	0.500	0.300	0.062	0.038	20	0.056	0.823	0.831
50	8	0.100	0.050	0.150	0.100	0.105	0.055	30	0.055	0.808	0.807
50	8	0.100	0.050	0.300	0.150	0.119	0.060	34	0.054	0.802	0.805
50	8	0.100	0.050	0.500	0.300	0.138	0.074	36	0.058	0.800	0.806

Web Table 6: Estimated required number of clusters $\widehat{n}_c$ for the HTE test based on the proposed formula, empirical type I error (Emp. Size), empirical power (Emp. Power), as well as predicted power (Pred. Power) for the HTE, test when randomization is at the participant level. For studying power, the HTE effect size is fixed at $\Delta_{\text{HTE}} = 0.1$.

Design Parameters							Performance Characteristics		
m	n_s	α_0	α_1	ρ_0	ρ_1	$\widehat{n}_c$	Emp. Size	Emp. Power	Pred. Power
20	4	0.015	0.010	0.150	0.100	40	0.047	0.808	0.813
20	4	0.015	0.010	0.300	0.150	40	0.048	0.810	0.813
20	4	0.015	0.010	0.500	0.300	40	0.048	0.806	0.813
20	4	0.100	0.050	0.150	0.100	36	0.050	0.801	0.807
20	4	0.100	0.050	0.300	0.150	36	0.050	0.798	0.807
20	4	0.100	0.050	0.500	0.300	36	0.048	0.798	0.807
20	8	0.015	0.010	0.150	0.100	20	0.050	0.806	0.813
20	8	0.015	0.010	0.300	0.150	20	0.048	0.798	0.813
20	8	0.015	0.010	0.500	0.300	20	0.048	0.792	0.813
20	8	0.100	0.050	0.150	0.100	18	0.053	0.792	0.807
20	8	0.100	0.050	0.300	0.150	18	0.052	0.797	0.807
20	8	0.100	0.050	0.500	0.300	18	0.051	0.799	0.807
50	4	0.015	0.010	0.150	0.100	16	0.053	0.817	0.813
50	4	0.015	0.010	0.300	0.150	16	0.054	0.809	0.813
50	4	0.015	0.010	0.500	0.300	16	0.052	0.798	0.813
50	4	0.100	0.050	0.150	0.100	16	0.054	0.844	0.846
50	4	0.100	0.050	0.300	0.150	16	0.053	0.840	0.846
50	4	0.100	0.050	0.500	0.300	16	0.052	0.827	0.846
50	8	0.015	0.010	0.150	0.100	8	0.060	0.798	0.813
50	8	0.015	0.010	0.300	0.150	8	0.060	0.793	0.813
50	8	0.015	0.010	0.500	0.300	8	0.057	0.780	0.813
50	8	0.100	0.050	0.150	0.100	8	0.060	0.828	0.846
50	8	0.100	0.050	0.300	0.150	8	0.060	0.822	0.846
50	8	0.100	0.050	0.500	0.300	8	0.059	0.812	0.846

Web Table 7: Estimated required number of clusters $\widehat{n}_c$ for the covariate-adjusted average treatment effect test based on the proposed formula, empirical type I error (Emp. Size), empirical power (Emp. Power), as well as predicted power (Pred. Power) for the covariate-adjusted average treatment effect test, when randomization is at the participant level. For studying power, the average treatment effect size is fixed at $\Delta_{\text{ATE}} = 0.1$.

Design Parameters							Performance Characteristics		
m	n_s	α_0	α_1	ρ_0	ρ_1	$\widehat{n}_c$	Emp. Size	Emp. Power	Pred. Power
20	4	0.015	0.010	0.150	0.100	40	0.046	0.817	0.813
20	4	0.015	0.010	0.300	0.150	40	0.046	0.818	0.813
20	4	0.015	0.010	0.500	0.300	40	0.048	0.817	0.813
20	4	0.100	0.050	0.150	0.100	36	0.053	0.809	0.807
20	4	0.100	0.050	0.300	0.150	36	0.054	0.808	0.807
20	4	0.100	0.050	0.500	0.300	36	0.054	0.806	0.807
20	8	0.015	0.010	0.150	0.100	20	0.056	0.809	0.813
20	8	0.015	0.010	0.300	0.150	20	0.056	0.811	0.813
20	8	0.015	0.010	0.500	0.300	20	0.057	0.808	0.813
20	8	0.100	0.050	0.150	0.100	18	0.052	0.813	0.807
20	8	0.100	0.050	0.300	0.150	18	0.052	0.811	0.807
20	8	0.100	0.050	0.500	0.300	18	0.055	0.811	0.807
50	4	0.015	0.010	0.150	0.100	16	0.056	0.811	0.813
50	4	0.015	0.010	0.300	0.150	16	0.055	0.810	0.813
50	4	0.015	0.010	0.500	0.300	16	0.056	0.806	0.813
50	4	0.100	0.050	0.150	0.100	16	0.055	0.841	0.846
50	4	0.100	0.050	0.300	0.150	16	0.056	0.839	0.846
50	4	0.100	0.050	0.500	0.300	16	0.057	0.833	0.846
50	8	0.015	0.010	0.150	0.100	8	0.053	0.809	0.813
50	8	0.015	0.010	0.300	0.150	8	0.054	0.807	0.813
50	8	0.015	0.010	0.500	0.300	8	0.060	0.802	0.813
50	8	0.100	0.050	0.150	0.100	8	0.053	0.841	0.846
50	8	0.100	0.050	0.300	0.150	8	0.055	0.839	0.846
50	8	0.100	0.050	0.500	0.300	8	0.061	0.835	0.846

Web Table 8: Estimated required number of clusters $\widehat{n}_c$ for the unadjusted average treatment effect test based on the Cunningham and Johnson (2016) formula, empirical type I error (Emp. Size), empirical power (Emp. Power), as well as predicted power (Pred. Power) for the unadjusted average treatment effect test, when randomization is at the participant level. For studying power, the average treatment effect size is fixed at $\Delta_{\text{ATE}} = 0.1$.

Design Parameters									Performance Characteristics		
m	n_s	α_0	α_1	ρ_0	ρ_1	$\widetilde{\alpha}_0$	$\widetilde{\alpha}_1$	$\widehat{n}_c$	Emp. Size	Emp. Power	Pred. Power
20	4	0.015	0.010	0.150	0.100	0.028	0.019	44	0.051	0.807	0.816
20	4	0.015	0.010	0.300	0.150	0.042	0.023	42	0.051	0.803	0.804
20	4	0.015	0.010	0.500	0.300	0.062	0.038	42	0.051	0.808	0.812
20	4	0.100	0.050	0.150	0.100	0.105	0.055	40	0.051	0.810	0.811
20	4	0.100	0.050	0.300	0.150	0.119	0.060	40	0.051	0.819	0.817
20	4	0.100	0.050	0.500	0.300	0.138	0.074	38	0.051	0.801	0.806
20	8	0.015	0.010	0.150	0.100	0.028	0.019	22	0.051	0.815	0.816
20	8	0.015	0.010	0.300	0.150	0.042	0.023	22	0.052	0.819	0.822
20	8	0.015	0.010	0.500	0.300	0.062	0.038	22	0.053	0.825	0.829
20	8	0.100	0.050	0.150	0.100	0.105	0.055	20	0.057	0.808	0.811
20	8	0.100	0.050	0.300	0.150	0.119	0.060	20	0.058	0.813	0.817
20	8	0.100	0.050	0.500	0.300	0.138	0.074	20	0.059	0.819	0.826
50	4	0.015	0.010	0.150	0.100	0.028	0.019	18	0.053	0.817	0.825
50	4	0.015	0.010	0.300	0.150	0.042	0.023	18	0.055	0.821	0.830
50	4	0.015	0.010	0.500	0.300	0.062	0.038	18	0.058	0.827	0.838
50	4	0.100	0.050	0.150	0.100	0.105	0.055	16	0.049	0.813	0.811
50	4	0.100	0.050	0.300	0.150	0.119	0.060	16	0.052	0.818	0.817
50	4	0.100	0.050	0.500	0.300	0.138	0.074	16	0.056	0.824	0.826
50	8	0.015	0.010	0.150	0.100	0.028	0.019	10	0.056	0.862	0.862
50	8	0.015	0.010	0.300	0.150	0.042	0.023	10	0.056	0.863	0.867
50	8	0.015	0.010	0.500	0.300	0.062	0.038	10	0.061	0.865	0.874
50	8	0.100	0.050	0.150	0.100	0.105	0.055	8	0.053	0.803	0.811
50	8	0.100	0.050	0.300	0.150	0.119	0.060	8	0.055	0.807	0.817
50	8	0.100	0.050	0.500	0.300	0.138	0.074	8	0.061	0.814	0.826

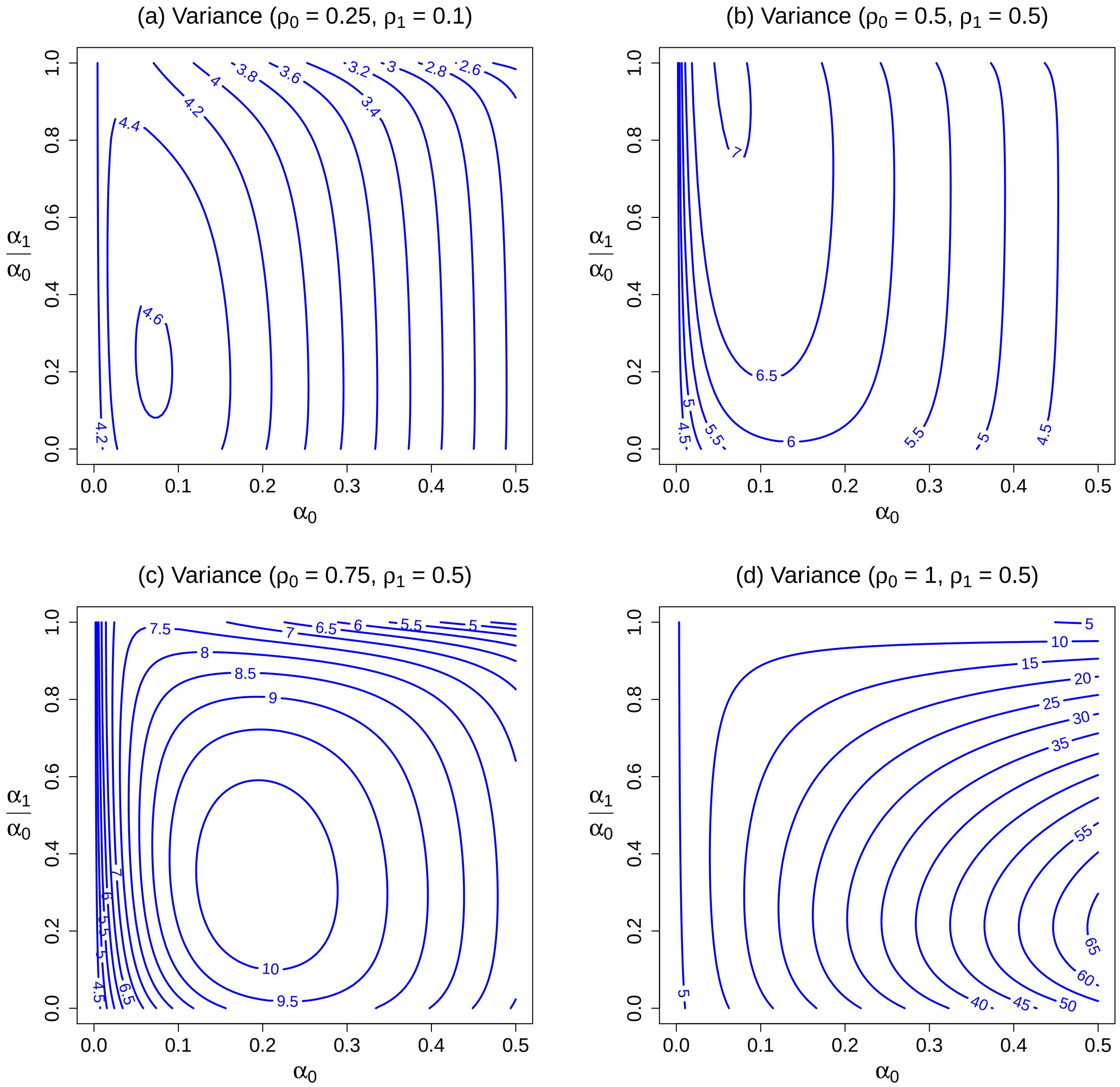

Web Figure 1: Contour of $\sigma^2_{4,(3)}$ (when randomization is carried out at the cluster level) as a function of outcome-ICCs, α_0 and α_1/α_0, for different values of covariate-ICCs. We purposely consider $\alpha_0 \in [0, 0.5]$ to graphically explore the relationship over a larger parameter space, even though outcome-ICCs in three-level design rarely exceed 0.2.

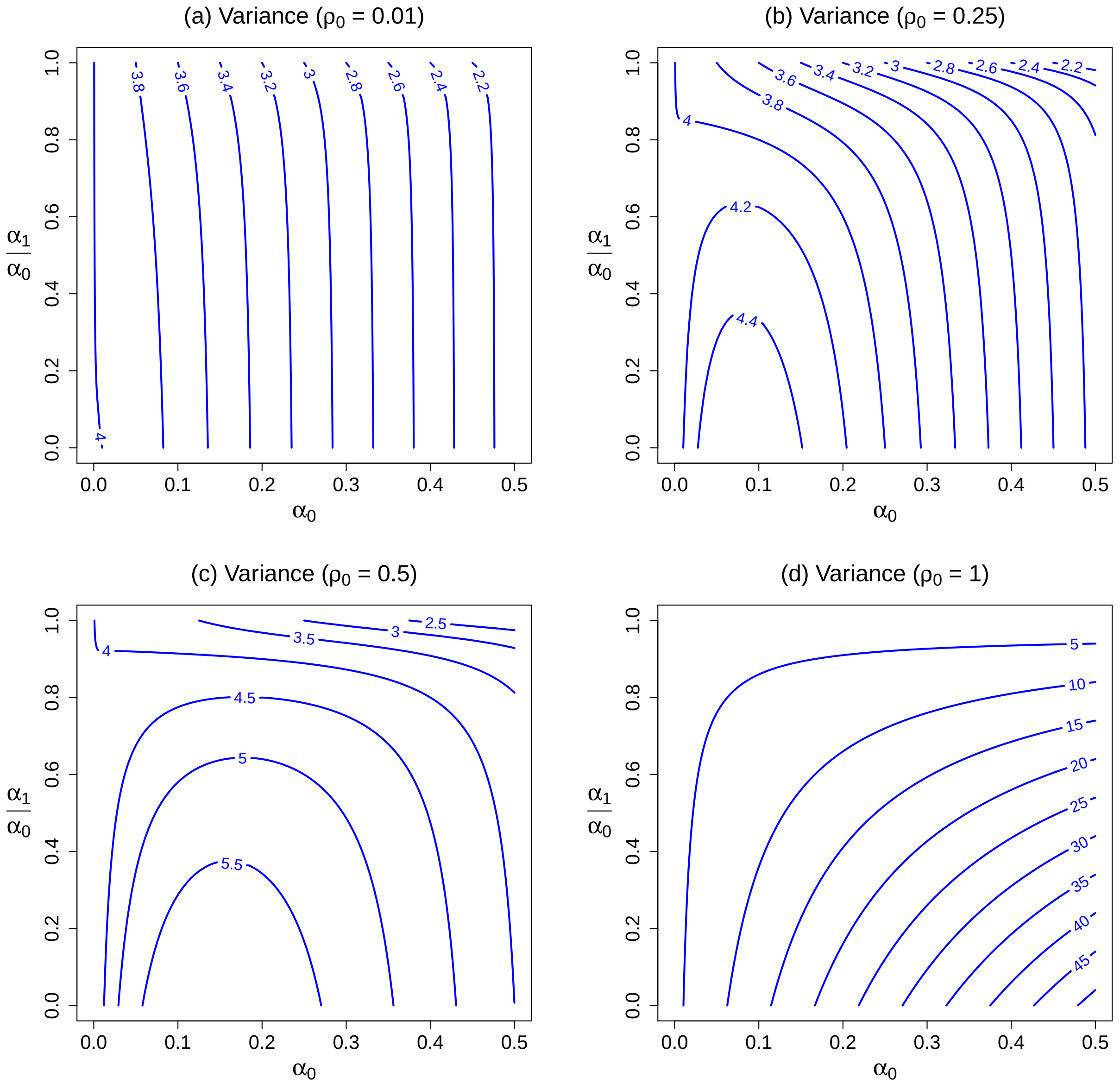

Web Figure 2: Contour of $\sigma^2_{4,(2)}$ (when randomization is carried out at the subcluster level) as a function of outcome-ICCs, α_0 and α_1/α_0, for different values of covariate-ICCs. We purposely consider $\alpha_0 \in [0, 0.5]$ to graphically explore the relationship over a larger parameter space, even though outcome-ICCs in three-level design rarely exceed 0.2.

References

Begg, M. D. and Parides, M. K. (2003). Separation of individual-level and cluster-level covariate effects in regression analysis of correlated data. *Statistics in Medicine* **22,** 2591–2602.

Cunningham, T. and Johnson, R. (2016). Design effects for sample size computation in three-level designs. *Statistical Methods in Medical Research* **25,** 505–519.

Dong, N., Kelcey, B., and Spybrook, J. (2018). Power analyses for moderator effects in three-level cluster randomized trials. *The Journal of Experimental Education* **86,** 489–514.

Harrison, D. A. and Brady, A. R. (2004). Sample size and power calculations using the noncentral t-distribution. *The Stata Journal* **4,** 142–153.

Li, F., Turner, E., and Preisser, J. (2018). Sample size determination for gee analyses of stepped wedge cluster randomized trials. *Biometrics* **74,** 1450–1458.

Raudenbush, S. (1997). Statistical analysis and optimal design for cluster randomized trials. *Psychological Methods* **2,** 173.

Yang, S., Li, F., Starks, M., et al. (2020). Sample size requirements for detecting treatment effect heterogeneity in cluster randomized trials. *Statistics in Medicine* **39,** 4218–4237.
